# Identification of Novel *β*‐Lactam Derivatives as Proteasome Inhibitors for Antitumor Therapy

**DOI:** 10.1002/ardp.70136

**Published:** 2025-11-21

**Authors:** Yu Cao, Gaoya Xu, Lixin Gao, Jingjing Sun, Lu Zhang, Qiao Tong, Limin Kong, Jiankang Zhang, Yubo Zhou, Li Liao, Liping Fu, Jianjun Xi

**Affiliations:** ^1^ Department of Pharmaceutical Preparation Hangzhou Xixi Hospital Hangzhou China; ^2^ State Key Laboratory of Chemical Biology, Shanghai Institute of Materia Medica Chinese Academy of Sciences Shanghai China; ^3^ Department of Clinical Pharmacy, the First Affiliated Hospital, School of Medicine Zhejiang University Hangzhou China; ^4^ Key Laboratory of Novel Targets and Drug Study for Neural Repair of Zhejiang Province, School of Medicine Hangzhou City University Hangzhou China; ^5^ Zhongshan Institute for Drug Discovery, Shanghai Institute of Materia Medica Chinese Academy of Sciences Zhongshan China; ^6^ Department of Pharmacy Hangzhou Children's Hospital Hangzhou China; ^7^ Department of Pharmacy Shaoxing TCM Hospital Affiliated to Zhejiang Chinese Medical University Shaoxing China

**Keywords:** antitumor, proteasome inhibitors, structure–activity relationships, *β*‐lactam

## Abstract

A series of dipeptidic proteasome inhibitors with *β*‐lactam as the *C*‐terminus was designed and synthesized. Biochemical evaluations of their effects on chymotrypsin‐like (CT‐L) activity revealed that some of them had inhibitory activity against the proteasome, with IC_50_ values in the micromolar range. Based on the enzymatic results, structure–activity relationships (SAR) were discussed in detail. Some potent compounds were further selected for antiproliferative assays toward multiple cancer cell lines, with compounds **66** and **78** demonstrating activity against RS4;11 cells and IC_50_ values of less than 1 μM. Additionally, cellular mechanistic studies indicated that these effects were linked to their ability to inhibit proteasome signal pathways and to induce apoptosis in RS4;11 cells, as demonstrated by flow cytometry. Collectively, these results illustrate that compound **66** is a potent proteasome inhibitor with significant potential as an antitumor agent.

## Graphical abstract

A series of dipeptidic proteasome inhibitors with *β*‐lactam as the *C*‐terminus was designed and synthesized. Compound **66** exhibited excellent proteasome inhibitory activities and displayed potent antiproliferative activities against RS4;11 cells.

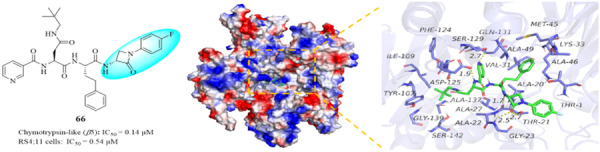

## 1 Introduction

The 26S proteasome is a sophisticated multi‐catalytic protease complex that operates in an ATP‐dependent manner, playing a vital role in the ubiquitin‐dependent conversion of cellular proteins [1]. It is composed of a 20S core particle flanked by two 19S regulatory subunits. The two 19S regulatory caps are equipped with numerous ATPase active sites and ubiquitin binding domains, which facilitate the recognition and unfolding of ubiquitinated proteins [2, 3]. The 20S core catalytic complex consists of two outer *α* rings and two inner *β* rings, with each ring containing seven *α* and *β*‐type subunits, respectively. Each *β* ring contains three proteolytically active *β* subunits exhibiting different substrate preferences: chymotrypsin‐like (CT‐L) on the *β*5 subunit, trypsin‐like (T‐L) on the *β*2 subunit, and caspase‐like (C‐L) on the *β*1 subunit [4, 5]. The CT‐L activity serves as the rate‐limiting step in the degradation of intracellular proteins, including those that contribute to tumor growth and survival [6, 7]. Consequently, the active sites of the 26S proteasome have been identified as potential therapeutic targets for a variety of diseases, including cancers [8, 9, 10, 11].

**Figure 1:**
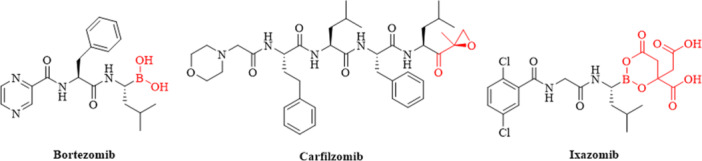
PIs in clinics.

For malignant tumor cells to expand, they rely on a markedly elevated rate of protein turnover. Besides rapid cell proliferation, tumor cells also feature a plethora of chromosomal and molecular abnormalities, which result in the generation of numerous distinct protein isoforms [12, 13, 14]. Collectively, these factors result in dysregulated protein expression, thereby imposing a significant burden on the ubiquitin‐proteasome system (UPS) [15]. Such considerations lay the groundwork for therapeutic strategies that target the UPS, particularly through the use of proteasome inhibitors (PIs) [16, 17]. It has been demonstrated that PIs can surmount resistance to specific chemotherapeutic drugs. PIs bortezomib, carfizomib, and ixazomib have been used in the treatment of hematological malignancy (MM) as the first‐line drug [9, 18, 19, 20] (Figure 1).

Nonetheless, the clinical applications of these compounds have been hindered by resistance and severe side effects, particularly in peripheral nerves, cardiac tissue, and the gastrointestinal tract [21, 22, 23]. The unique pharmacophore boronic acid and epoxyketone could form tetrahedral adducts and six‐membered morpholine ring with the active site of threonines, respectively; however, these interactions may cause serious neurotoxicity and cardiotoxicity [2, 9, 24]. Because of the limitations and challenges of the PIs currently approved for MM treatment, there is a need to discover novel PIs that are more effective and safer.

**Figure 2:**
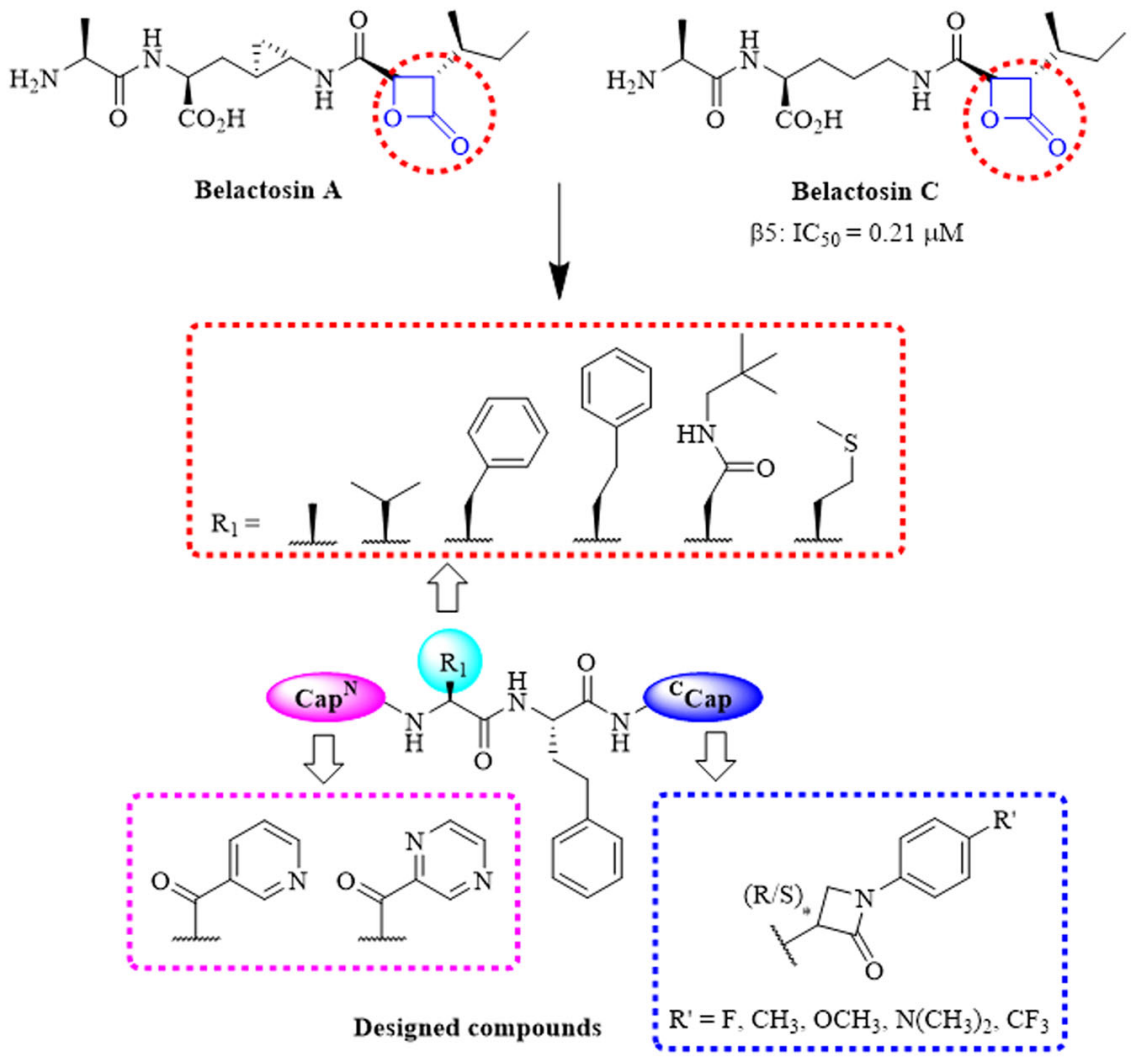
The design of *β*‐lactam derivatives.

In 2000, Asai and colleagues isolated belactosin A and C from a *Streptomyces* species [25, 26], as illustrated in Figure 2. Both of them inhibited the CT‐L activity of rabbit 20S proteasome. The inhibitory potency of belactosins toward the proteasome appears to be attributed to the *β*‐lactone moiety, which undergoes nucleophilic ring opening by the hydroxyl group of the N‐terminal threonine residue, resulting in the formation of a covalent bond [27]. Considering the instability of the lactones, we attempted to replace the lactone ring with the *β*‐lactam ring. Despite the significant role of *β*‐lactam rings in antibacterial agents, their application in other areas of medicinal chemistry has remained surprisingly limited [28]. Herein, we report peptidomimetic inhibitors of proteasome bearing a *C*‐terminal *β*‐lactam moiety. We examined the effect of various peptide skelectons on the activity, as well as the influence of the stereochemistry at the *β*‐lactam *C*‐3 stereocenter and the electronic effect of the *N*‐1 aryl substituent (Figure 2). Alongside the evaluation of the activity in biochemical and cellular assays, the inhibition mechanism of the most potent compounds was investigated.

## 2 Results and Discussion

### 2.1 Chemistry

**Scheme 1:**
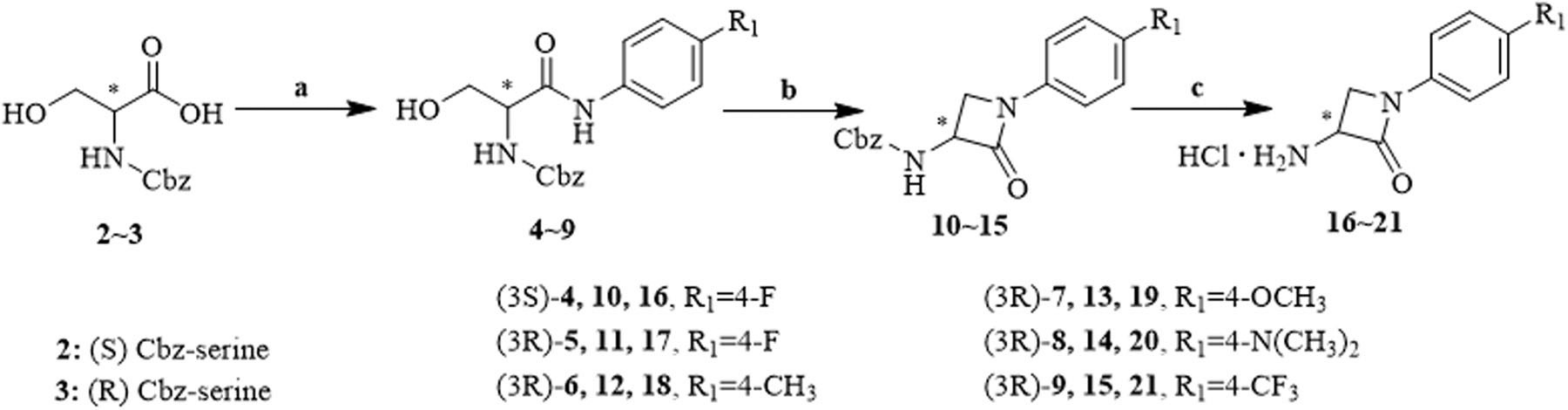
Synthesis of *β*‐lactams **16**~**21**. Reagents and conditions: (a) COMU, TMP, corresponding aniline, DMF, 0°C‐rt; (b) Im_2_SO_2_, DMF, 0°C; NaH, −20°C; (c) Pd/C, H_2_, EtOH, rt.

*β*‐Lactams **16**~**21** were synthesized through a three‐step reaction sequence initiated from Cbz‐protected serine. This method permits the preparation of enantiopure (3*R*)‐ or (3*S*)‐*β*‐lactams using the predetermined chirality of (*R*)‐ or (*S*)‐serine, respectively. Furthermore, this approach yields *β*‐lactams featuring an aryl substituent at the *N*‐1 position and a free amino group at the *C*‐3 position, which enables coupling to the peptides (Scheme 1). The first step involves the coupling of an aryl amine to the *C*‐terminus of serine, employing COMU as a coupling reagent and TMP as a base. The critical step is the cyclization of (*R*)‐ or (*S*)‐amides **4**~**9** into (3*R*)‐ or (3*S*)‐*β*‐lactams **10**~**15**. This transformation was facilitated through the addition of 1,1‐sulfonyldiimidazole and sodium hydride. Finally, the Cbz‐protection was removed utilizing hydrogen and palladium on charcoal, resulting in (3*R*)‐ or (3*S*)‐3‐amino‐*β*‐lactams **16**~**21**.

**Scheme 2:**
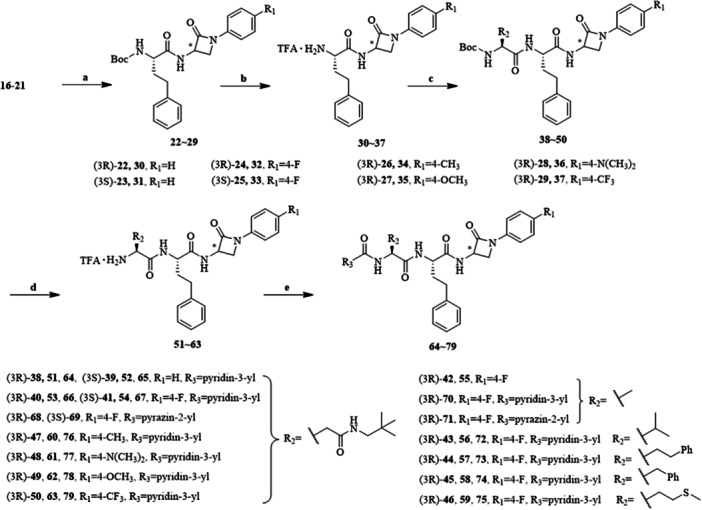
Synthesis of target compounds **64**~**79**. Reagents and conditions: (a) HOBt, EDCI, Boc‐l‐homophenylalanine, DIPEA, DCM, 0°C‐rt; (b) TFA, DCM, 0°C‐rt; (c) HOBt, EDCI, corresponding amino acids, DIPEA, DCM, 0°C‐rt; (d) TFA, DCM, 0°C‐rt; (e) HOBt, EDCI, nicotinic acid or 2‐pyrazinecarboxylic acid, DIPEA, DCM, 0°C‐rt.

The targeted compounds **64**~**79** were synthesized as shown in Scheme 2. Initially, *β*‐lactams **16**~**21** were coupled to Boc‐l‐homophenylalanine using EDCI as a coupling reagent and DIPEA as a base. (3*R*)‐ or (3*S*)‐*β*‐lactam derivatives **30**~**37** were obtained after Boc deprotection using trifluoroacetic acid. Boc‐protected amino acids were coupled to intermediates **30**~**37**, followed by cleaving Boc‐protection, yielding (3*R*)‐ or (3*S*)‐dipeptide‐*β*‐lactam derivatives **51**~**63**. Dipeptide‐*β*‐lactam derivatives **51**~**63** reacted with nicotinic acid or 2‐pyrazinecarboxylic acid under the previously described conditions to afford final products **64**~**79**.

### 2.2 Pharmacology/Biology

#### 2.2.1 The Inhibitory Activities Against Proteasome and SAR Analysis

As illustrated in Table 1, the target compounds **64**~**79** were screened for their in vitro proteasome CT‐L inhibitory activities, with bortezomib utilized as a positive control. In the initial series of compounds **64**~**69**, which featured enantiopure (3*R*)‐ and (3*S*)‐ *β*‐lactams at the *C*‐terminal, those substituted with (3*S*)‐*β*‐lactams showed markedly diminished activity (IC_50_: > 10,000 nM for 65 vs. IC_50_: 186.85 ± 35.28 nM for 64; IC_50_: 1474.00 ± 280.01 nM for 67 vs. IC_50_: 114.70 ± 1.70 nM for 66; IC_50_: > 10,000 nM for 68 vs. IC_50_: 288.45 ± 57.20 nM for 69). The data underscore the critical influence of steric configurations at the *C*‐3 position on the retention of activity.

**Table 1:** In vitro enzyme activity of compounds **64**~**79**.

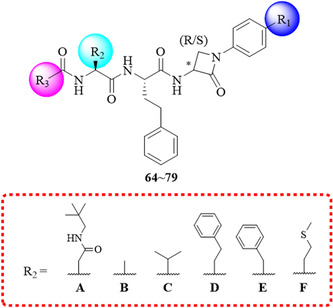						
Compound	R_1_	R_2_	R_3_	*β*5: IC_50_ (nM)^a^	*β*1: IC_50_ (nM)^a^	*β*2: IC_50_ (nM)^a^
**64‐**(3*R*)	H	A	Pyridine‐3‐yl	186.85 ± 35.28	> 10,000	> 10,000
**65‐**(3*S*)	H	A	Pyridine‐3‐yl	> 10,000	—	—
**66‐**(3*R*)	F	A	Pyridine‐3‐yl	114.70 ± 1.70	> 10,000	> 10,000
**67‐**(3*S*)	F	A	Pyridine‐3‐yl	1474.00 ± 280.01	—	—
**68‐**(3*S*)	F	A	Pyrazin‐2‐yl	> 10,000	—	—
**69‐**(3*R*)	F	A	Pyrazin‐2‐yl	288.45 ± 57.20	> 10,000	> 10,000
**70‐**(3*R*)	F	B	Pyridine‐3‐yl	> 10,000	—	—
**71‐**(3*R*)	F	B	Pyrazin‐2‐yl	> 10,000	—	—
**72‐**(3*R*)	F	C	Pyridine‐3‐yl	> 10,000	—	—
**73‐**(3*R*)	F	D	Pyridine‐3‐yl	> 10,000	—	—
**74‐**(3*R*)	F	E	Pyridine‐3‐yl	> 10,000	—	—
**75‐**(3*R*)	F	F	Pyridine‐3‐yl	> 10,000	—	—
**76‐**(3*R*)	CH_3_	A	Pyridine‐3‐yl	72.03 ± 14.89	> 10,000	> 10,000
**77‐**(3*R*)	N(CH_3_)_2_	A	Pyridine‐3‐yl	132.25 ± 14.78	> 10,000	> 10,000
**78‐**(3*R*)	OCH_3_	A	Pyridine‐3‐yl	141.60 ± 30.12	> 10,000	> 10,000
**79‐**(3*R*)	CF_3_	A	Pyridine‐3‐yl	282.35 ± 33.60	> 10,000	> 10,000
Bortezomib	—	—	—	14.81 ± 0.35	—	—
Carfilzomib	—	—	—	8.62 ± 5.35	338.90 ± 406.00	584.00 ± 539.30

^a^ Values represent the mean ± SD of three independent experiments, each based on three biological replicates.

Subsequently, when (3*R*)‐ *β*‐lactam was retained at the *C*‐terminal and the F group was incorporated into the benzene ring, further exploration of the dipeptide skeleton was undertaken in the second round of modifications. Regrettably, among the various substitutions (A–F) introduced at the R_2_ position, only the compound with an A substitution exhibited significant inhibitory activity against the proteasome (IC_50_: 114.70 ± 1.70 nM for 66), while compounds featuring other skeletal replacements (B–F) showed no activity (IC_50_: > 10,000 nM for 70, 72–75). Notably, based on the proteasome inhibitory activities of **66** and **69**, it was observed that the introduction of pyridine‐3‐yl or pyrazin‐2‐yl at the R_3_ position had minimal impact on the activity.

Building upon the insights gleaned from the first and second rounds of proteasome inhibition assessments, a third round of structural modifications was conducted using compound **66** as the reference, maintaining the *R* configuration at the *C*‐3 position, retaining substitution A at the R_2_ position, and incorporating pyridine‐3‐yl at the *N*‐terminal, while varying the substitutions on the benzene ring. This included both unsubstituted benzene (compound **64**) and substitutions with electron‐donating substituents such as methyl (compound **76**), dimethylamino (compound **77**), and methoxy (compound **78**) groups, as well as electron‐withdrawing substitutions including trifluoromethyl (compound **79**). The results indicated that diverse substitutions on the benzene ring had a negligible effect on the activity. Notably, the introduction of a methyl group on the benzene resulted in compound **76**, which exhibited robust inhibitory activity against the proteasome with an IC_50_ value of 72.03 ± 14.89 nM. To evaluate the target selectivity profile, selected CT‐L inhibitory compounds were further screened against two additional proteolytically active subunits, T‐L on the *β*2 subunit and C‐L on the *β*1 subunit, with no detectable inhibitory activity observed.

#### 2.2.2 Antiproliferative Activities

Then, we conducted cellular anti‐proliferation assays of compounds **64**, **66**, **69**, **76**, **78**, and **79** in malignant hematological tumor cell lines, specifically MV‐4‐11, RPMI‐8226, Molt4, and RS4;11, as well as normal peripheral blood mononuclear cells (PBMC). The cellular activities of target compounds are summarized in Table 2, with bortezomib serving as the reference standard. Among malignant hematological tumor cells, human acute leukemia cell line RS4;11 was more sensitive to the tested compounds, with IC_50_ values recorded at less than 2 μM. Notably, compounds **66** and **78** showed the highest antiproliferative efficacy, achieving IC_50_ values of 0.54 nM and 0.50 nM, respectively. Furthermore, compounds **66** and **78** displayed lower cytotoxicity against normal PBMC compared with (**66**, IC_50_: 4900 nM; 78, IC_50_: 17180 nM) the positive control bortezomib (IC_50_: 1.57 nM).

**Table 2:** Antiproliferative activity of representative compounds.

	IC_50_ (μM)^a^				
Compound	MV‐4‐11	RPMI‐8226	Molt4	RS4;11	PBMC‐3Day
**64**	7.42 ± 0.62	5.52 ± 1.12	8.34 ± 0.18	1.09 ± 0.31	—
**66**	1.78 ± 0.19	3.02 ± 0.14	2.68 ± 0.73	0.54 ± 0.18	4.90 ± 0.68
**69**	> 20	10.18 ± 0.76	> 10	1.52 ± 0.67	—
**76**	> 20	8.05 ± 1.17	> 10	0.86 ± 0.24	—
**78**	2.78 ± 0.41	2.34 ± 0.34	3.78 ± 0.27	0.50 ± 0.13	17.18 ± 1.93
**79**	2.75 ± 0.46	3.44 ± 0.44	2.37 ± 0.23	0.99 ± 0.17	—
Bortezomib	0.00515 ± 0.00109	0.00364 ± 0.00052	0.01327 ± 0.00247	0.00587 ± 0.00093	0.00157 ± 0.00019

^a^ Values represent the mean ± SD of three independent experiments, each based on three biological replicates.

#### 2.2.3 Cellular Mechanistic Studies

**Figure 3:**
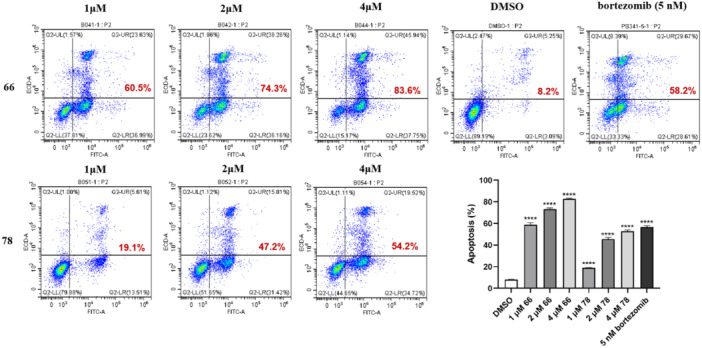
The effect of **66** and **78** on apoptosis of RS4;11. RS4;11 cells were cultured normally or incubated with medium containing 66 (1, 2, and 4 *μ*M), 78 (1, 2, and 4 *μ*M), and 5 nM bortezomib for 12 h. Bar graphs illustrate cell subpopulations labeled with annexin V‐FITC, representing early and late apoptotic stages as assessed by flow cytometry. Results were mean ± SD for three individual experiments. ****p < 0.0001 compared with DMSO (one‐way ANOVA).

To further elucidate the potential of compounds **66** and **78** in inducing apoptosis in the human acute leukemia cell line, we assessed their effects on apoptosis in RS4;11 cells via an apoptosis assay. As illustrated in Figure 3, compounds **66** and **78** triggered cell apoptosis in a dose‐dependent manner. Compound **66** exhibited a more pronounced capacity to facilitate apoptosis compared with **78**.

**Figure 4:**
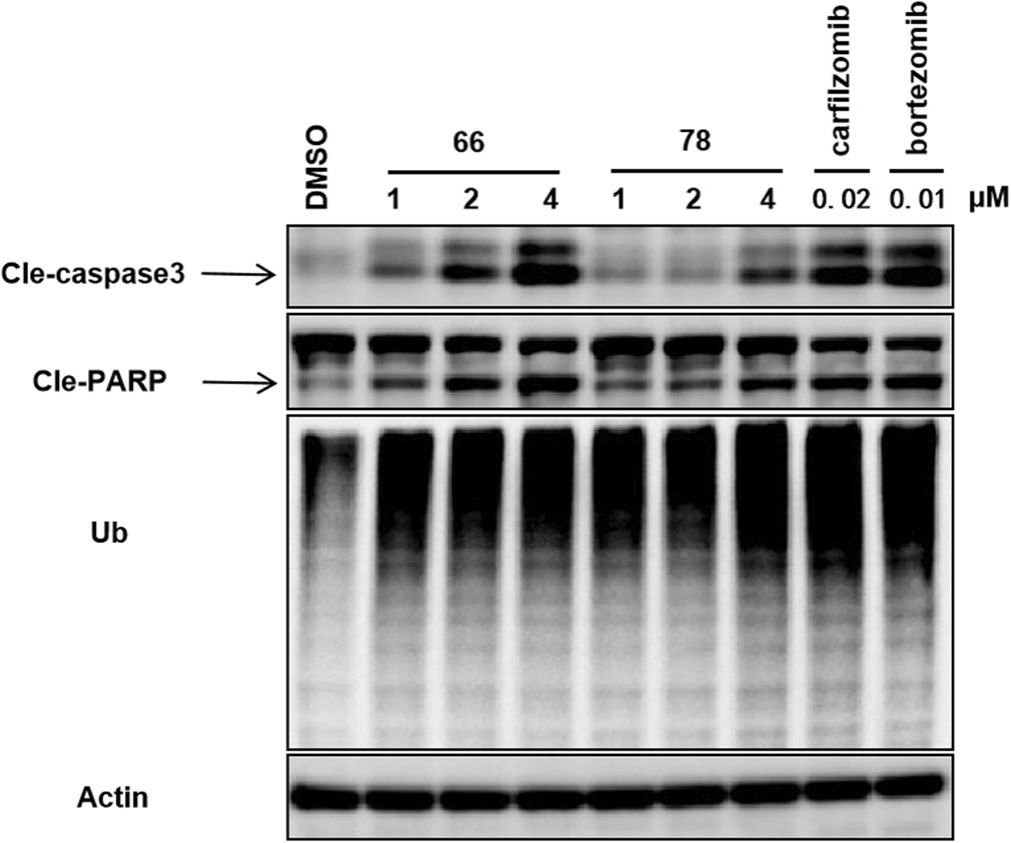
The expression of apoptosis‐related proteins Cle‐caspase and Cle‐PARP, treated with increasing concentrations 1, 2, and 4 *μ*M) of **66** and **78**, was examined using western blot analysis. Accumulation of ubiquitinated proteins induced by **66** and **78**.

Subsequently, we examined the influence of these compounds on apoptosis‐related proteins using western blot assays in RS4;11 cells. The cell line was treated with three concentrations (1, 2, and 4 μM) of compounds **66** and **78** for a duration of 8 h. As depicted in Figure 4, the activation and cleavage of caspase‐3 and PARP were detectable across all samples, indicating their key role in the induction of apoptosis. Notably, the intensity of these signals increased with higher concentrations, corroborating the findings from flow cytometry. Furthermore, **66** and **78** exhibited a significant accumulation of poly‐ubiquitinated proteins, which aligns with that of the positive controls bortezomib and carfilzomib.

### 2.3 Molecular Docking Study

Molecular docking plays a vital role in elucidating possible protein–target molecule interactions and identifying binding locations. This computational methodology aims to determine the ideal conformation that minimizes the free energy of the system. Through accurate prediction of intermolecular interactions, molecular modeling significantly facilitates the design and development of new therapeutic agents by prioritizing the most promising candidate structures for subsequent experimental validation and development. This docking study was conducted using *Glide* (*Schrödinger version 2021‐2*).

**Figure 5:**
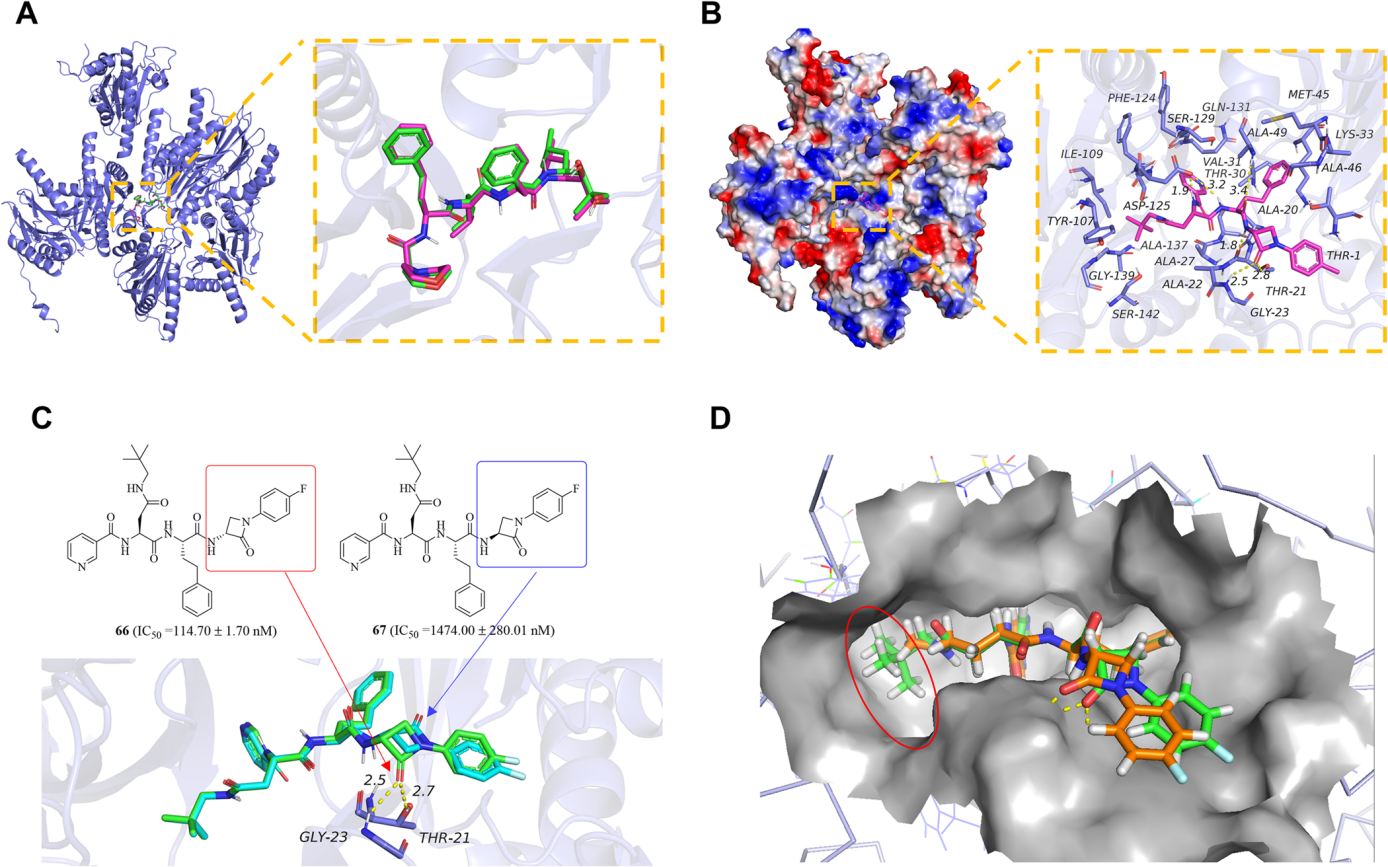
Comprehensive analysis of molecules **66**, **67**, **75**, and **76** and human constitutive 20S proteasome (PDB ID code: 4R3O) interactions. (A) The redocking results for 4R3O with nature ligand. (B) The detailed binding mode between **76** and proteasome (PDB ID code: 4R3O). The backbone of the protein was rendered in tube and colored in slate. Compound is rendered in red. (C) The detailed binding mode of molecules **66 and 67** with the complex. The backbone of the protein was rendered in tube and colored in light blue. Compound **66** is rendered in green. Compound **67** is rendered in cyan. (D) Structural comparison of proteasome *β*5 site occupancy. Compound **66** (green) with neopentyl‐asparagine optimally filling the specificity pocket. Compound **75** (orange) showing inefficient hydrophobic pocket interactions.

The docking study was conducted using the human constitutive 20S proteasome with the PDB ID: 4R3O. A grid box of 15 Å × 15 Å × 15 Å was defined around the native ligand of the selected protein with coordinates (x,y,z): 177.34, −25.55, and 1.82 to encompass the binding region. To validate the docking parameters, we performed redocking experiments using the protein's native ligand. As shown in Figure 5A, the redocked ligand demonstrated excellent structural alignment with the crystallographic conformation (RMSD < 1.78 Å), confirming the reliability of our docking methodology and parameter settings.

Revealing assorted binding interactions, the most bioactive compound **76** (IC_50_ = 72.03 ± 14.89 nM) demonstrated a docking score of −9.619 kcal/mol with the target protein 4R3O. Structural analysis revealed critical hydrogen‐bonding interactions between molecule 76 and key amino acid residues within the binding pocket. Residue THR Z:21 developed hydrogen bonds (1.8 and 2.8 Å) with NH‐proton and the O‐atom in the lactam ring. Likewise, the O‐atom of the lactam ring unveiled one hydrogen bond at a distance of 2.5 Å with residue GLY Z:23. Further, the residue ASP 1:125 produced a hydrogen bond with the NH‐proton of N‐terminus at a distance of 1.9 Å (Figure 5B). A comparison of the docking simulation studies of compounds **66** (IC_50_ = 114.70 ± 1.70 nM) and **67** (IC_50_ = 1474.00 ± 280.01 nM) underscored the significance of chirality. As depicted in Figure 5C, the carbonyl group of molecule **66**, containing (3 *R*)‐ *β*‐lactam, participated in hydrogen bonding with THR Z:21 (2.7 Å) and GLY Z:23 (2.5 Å). In contrast, molecule **67**, which incorporates the (3*S*)‐ *β*‐lactam, failed to form hydrogen bonds in the region due to the altered orientation of its carbonyl group. Additionally, we intentionally included compound **75** (IC_50_ > 10,000 nM) as an inactive control for comparative analysis. The striking potency difference between compound **66** (IC_50_ = 114.70 ± 1.70 nM), featuring a neopentyl‐asparagine group at the R_2_ position, and the inactive methionine‐containing analog (compound **75**) can be structurally rationalized by the superior complementarity of the neopentyl‐asparagine moiety within the hydrophobic binding pocket (Figure 5D).

### 2.4 Molecular Dynamics (MD) Simulation

**Figure 6:**
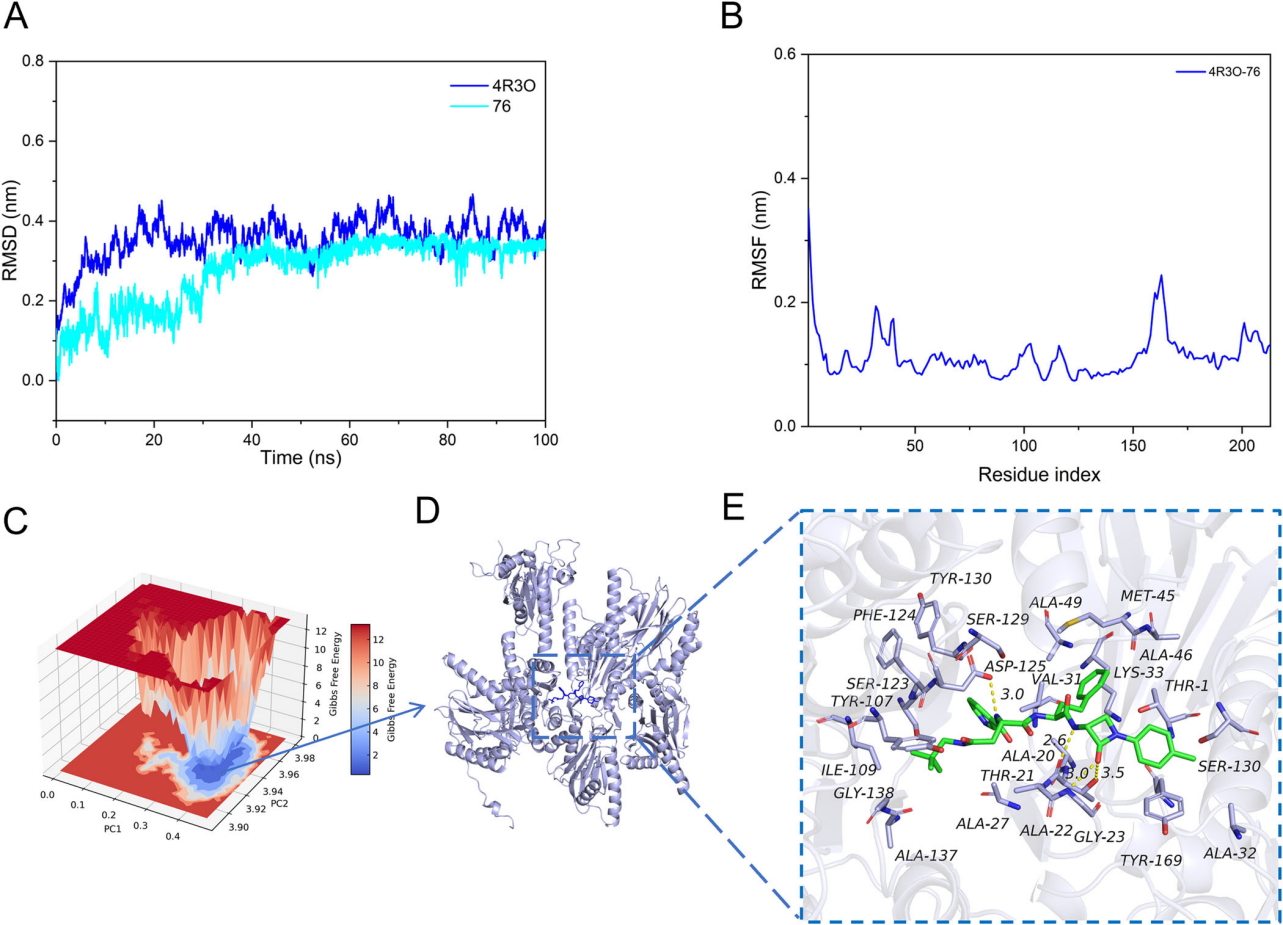
MD simulation plots for complexes “4R3O‐**76**” (PDB ID code: 4R3O) interactions. (A) Root mean square deviation (RMSD) value of proteasome (blue) and molecule **76** (cyan). (B) Root mean square fluctuation (RMSF) value of the proteasome. (C) The 3D free energy landscape of the complex. (D). The 3D structure of the complex. (E) The detailed binding mode between **76** and proteasome (PDB ID code: 4R3O). The backbone of the protein was rendered in tube and colored in light blue. Compound is rendered in green.

MD simulations are effective tools to uncover the intricate dynamics and stability of protein–ligand complexes, essential for drug development. In this study, we conducted extensive simulations of the human constitutive 20S proteasome (PDB ID code: 4R3O) and molecule **76** using Gromacs 2020, spanning a simulation duration of 100 ns to capture the dynamic changes in the system over time. In our investigation of 4R3O‐**76** complex, the root mean square deviation (RMSD) values for the ligands remained consistently below 4 Å throughout the simulation, indicating structural stability (Figure 6A). To monitor local changes in the protein chains, the root mean square fluctuation (RMSF) parameter was employed. The RMSF analysis further revealed fluctuations, particularly around residue index 170 and within the range of 200‐300, suggesting these dynamic variations stem from the positioning of these amino acids in the protein's hinge region. In contrast, most residues exhibit minimal conformational changes, indicating that molecule **76** forms a stable complex with the proteasome (Figure 6B).

The binding free energy of molecule **76** to the proteasome was calculated to be −30.23 ± 4.54 kcal/mol, with van der Waals interactions contributing predominantly (−58.13 ± 0.46 kcal/mol) (Figure 6C). This indicates that the compound stably occupied the binding pocket, engaging in extensive vdW interactions with surrounding residues. This strong interaction arises primarily due to the compound being deeply inserted into the protein cavity, forming favorable van der Waals contacts with nearby amino acids. Additionally, hydrogen bonding between the compound and the proteasome significantly enhances electrostatic stabilization (−31.17 ± 4.39 kcal/mol). Collectively, these interactions indicate that molecule **76** binds the proteasome with high affinity, forming a stable complex that likely underpins its biological activity (Figure 6D,E).

## 3 Conclusion

In this study, we aimed to mitigate the severe side effects associated with the approved proteasome inhibitor. Inspired by the *β*‐lactone moiety of belactosines, we designed and synthesized a series of dipeptides featuring enantiopure (3*R*)‐ and (3*S*)‐*β*‐lactams. Evaluation of the proteasome inhibitory activities revealed that target compounds, which include substitution A and (3*R*)‐*β*‐lactam within the peptidomimetic skeleton, exhibited potent proteasome inhibition, with IC_50_ values being below 300 μM. Furthermore, the antitumor assays demonstrated that compounds **66** and **78** exhibited strong inhibitory activity against RS4;11 cells. Fortunately, these compounds displayed minimal potency against PBMC cells, suggesting that they might have no toxic effects. Biochemical analyses confirmed that compounds **66** and **78** could induce apoptosis in RS4;11 cells, as evidenced by flow cytometry, and resulted in massive accumulation of poly‐ubiquitinated proteins. Furthermore, we explored the explicit binding interactions of molecule **66** with proteasome using molecular docking. Our analysis revealed that molecule 66 engages in hydrogen bond interactions with Thr21 and Gly23. These findings indicate that compound **66** holds promise as a potential therapeutic agent for the treatment of cancer through proteasome inhibition. However, a significant limitation of the synthesized compound is its poor stability in rat plasma, indicating rapid enzymatic degradation and chemical hydrolysis. This instability poses a considerable challenge to its *in vivo* efficacy and pharmacokinetic profile. Future research will focus on addressing this issue through strategic molecular modifications to enhance metabolic stability.

## 4 Experimental

### 4.1 Chemistry

#### 4.1.1 General

All reagents and solvents were procured from commercial sources and utilized without any further purification. NMR spectra (see the Supporting Information) were recorded for ^1^H NMR at 400/500 MHz and for ^13^C NMR at 100/125 MHz. For ^1^H NMR, CDCl_3_ (*δ* = 7.26) and DMSO‐*d*_6_ (*δ* = 2.50) were employed as internal standards, and data are reported as follows: chemical shift, multiplicity (s = singlet, d = doublet, t = triplet, q = quartet, m = multiplet), coupling constant in Hz, and integration. HRAM‐MS data were obtained using an Agilent 1290 HPLC‐6224 Time of Flight Mass Spectrometer. High‐performance liquid chromatography (HPLC) analyses were performed on an Agilent 1200 system, which was equipped with a photodiode array detector using a ZORBAX SB‐C18 column (4.6 × 250 mm) and detected at 254 nm wavelength. The samples were eluted with a 70:30 methanol/H_2_O mixture at a flow rate of 1 mL/min. All yields reported are unoptimized and generally represent typical outcomes. The InChI codes of the investigated compounds, together with some biological activity data, are provided as Supporting Information.

#### 4.1.2 General Procedure for the Synthesis of (*R*)‐ or (*S*)‐Amides 4~9

Cbz‐protected (*S*)‐ or (*R*)‐serine (2 or 3, 1.0 equiv) was dissolved in DMF (4 mL) within the reaction flask. Corresponding anilines (1.0 equiv) and TMP (1.1 equiv) were subsequently introduced to the flask. The temperature was lowered to 0°C, and COMU (1.1 equiv) was then added. The reaction proceeded at 0°C for 1 h, followed by an additional 3 h at room temperature. Ethyl acetate (30 mL) was added, and the resulting solution was washed with 1 M HCl (2 × 25 mL), saturated NaHCO_3_ (2 × 25 mL), and saturated NaCl solution (2 × 25 mL). The organic layer was dried over Na_2_SO_4_ and concentrated by evaporation to yield a solid, which was used directly in the next step without purification.

Benzyl(*S*)‐{1‐[(4‐fluorophenyl)amino]‐3‐hydroxy‐1‐oxopropan‐2‐yl}carbamate (**4**): white solid, 65%, ^1^H NMR (500 MHz, DMSO‐*d*_6_) *δ* 10.08 (s, 1H, NH), 7.65 (d, *J* = 9.0 Hz, 2H, Ar‐H), 7.37–7.34 (m, 6H, Ar‐H + NH), 7.16–7.13 (m, 2H, Ar‐H), 5.05 (s, 2H, CH_2_), 5.02–5.00 (m, 1H, OH), 4.22 (dd, *J* = 13.6, 6.4 Hz, 1H, CH), 3.66–3.62 (m, 2H, CH_2_).

Benzyl(*R*)‐[3‐hydroxy‐1‐oxo‐1‐(p‐tolylamino)propan‐2‐yl]carbamate (**6**): white solid, 73%, ^1^H NMR (500 MHz, DMSO‐*d*_6_) *δ* 9.90 (s, 1H, NH), 7.50 (d, *J* = 8.0 Hz, 2H, Ar‐H), 7.37 (d, *J* = 4.0 Hz, 3H, Ar‐H), 7.34–7.18 (m, 3H, Ar‐H + NH), 7.10 (d, *J* = 8.0 Hz, 2H, Ar‐H), 5.04 (s, 2H, CH_2_), 4.97 (t, *J* = 5.5 Hz, 1H, CH), 4.25–4.19 (m, 1H, CH), 3.69–3.58 (m, 2H, CH_2_), 2.25 (s, 3H, CH_3_).

Benzyl(*R*)‐{3‐hydroxy‐1‐[(4‐methoxyphenyl)amino]‐1‐oxopropan‐2‐yl}carbamate (**7**): white solid, 69%,^1^H NMR (400 MHz, DMSO‐*d*_6_) *δ* 9.85 (s, 1H, NH), 7.52 (d, *J* = 9.0 Hz, 2H, Ar‐H), 7.41–7.19 (m, 6H, Ar‐H + NH), 6.90–6.85 (m, 2H, Ar‐H), 5.04 (s, 2H, CH_2_), 5.01–4.93 (m, 1H, OH), 4.20 (dd, *J* = 13.8, 6.0 Hz, 1H, CH), 3.71 (s, 3H, CH_3_), 3.69–3.56 (m, 2H, CH_2_).

Benzyl(*R*)‐(1‐{[4‐(dimethylamino)phenyl]amino}‐3‐hydroxy‐1‐oxopropan‐2‐yl)carbamatee (**8**): white solid, 74%, ^1^H NMR (400 MHz, DMSO‐*d*_6_) *δ* 9.69 (s, 1H, NH), 7.45–7.26 (m, 9H, Ar‐H), 6.68 (d, *J* = 8.8 Hz, 1H, NH), 5.04 (s, 2H, CH_2_), 4.99–4.93 (m, 1H, OH), 4.19 (dd, *J* = 13.6, 6.4 Hz, 1H, CH), 3.69–3.57 (m, 2H, CH_2_), 2.84 (s, 6H, CH_3_).

Benzyl(*R*)‐(3‐hydroxy‐1‐oxo‐1‐{[4‐(trifluoromethyl)phenyl]amino}propan‐2‐yl)carbamate (**9**): white solid, 70%, ^1^H NMR (400 MHz, DMSO‐*d*_6_) *δ* 10.41 (s, 1H, NH), 7.84 (d, *J* = 8.8 Hz, 2H, Ar‐H), 7.69 (d, *J* = 8.4 Hz, 2H, Ar‐H), 7.44 (d, *J* = 7.6 Hz, 1H, NH), 7.40–7.20 (m, 5H, Ar‐H), 5.11–5.00 (m, 3H, CH_2_ + OH), 4.26 (dd, *J* = 13.5, 6.0 Hz, 1H, CH), 3.74–3.62 (m, 2H, CH_2_).

#### 4.1.3 General Procedure for the Synthesis of 3(*R*)‐ or 3(*S*)‐*β*‐Lactams 10~15

To a solution of (*R*)‐ or (*S*)‐amides 4~9 (1.0 equiv) in DMF (3 mL), 1,1′‐sulfonyldiimidazole (1.5 equiv) was added at 0°C. The mixture was stirred for 30 min at 0°C. Then the temperature was lowered to −20°C and NaH (1.5 equiv) was added. After completion of the reaction, the mixture was poured into mixed solvent (MeOH/H_2_O = 100:1). The precipitate was filtered and dried to give a crude solid 10~15.

Benzyl (*S*)‐[1‐(4‐fluorophenyl)‐2‐oxoazetidin‐3‐yl]carbamate (**10**): white solid, 87%, ^1^H NMR (400 MHz, DMSO‐*d*_6_) *δ* 8.11 (d, *J* = 8.4 Hz, 1H, NH), 7.76–7.62 (m, 2H, Ar‐H), 7.43–7.35 (m, 5H, Ar‐H), 7.23 (t, *J* = 8.4 Hz, 2H, Ar‐H), 5.06 (s, 2H, CH_2_), 4.92–4.84 (m, 1H, CH), 3.94 (t, *J* = 5.6 Hz, 1H, CH_2_), 3.59 (dd, *J* = 5.2, 2.8 Hz, 1H, CH_2_).

Benzyl (*R*)‐[2‐oxo‐1‐(p‐tolyl)azetidin‐3‐yl]carbamate (**12**): white solid, 87%, ^1^H NMR (500 MHz, DMSO‐*d*_6_) *δ* 8.10 (d, *J* = 8.5 Hz, 1H, NH), 7.41–7.29 (m, 5H, Ar‐H), 7.25 (d, *J* = 8.0 Hz, 2H, Ar‐H), 7.18 (d, *J* = 8.0 Hz, 2H, Ar‐H), 5.06 (s, 2H, CH_2_), 4.90–4.84 (m, 1H, CH), 3.91 (t, *J* = 6.0 Hz, 1H, CH_2_), 3.56 (dd, *J* = 5.5, 3.0 Hz, 1H, CH_2_), 2.27 (s, 3H, CH_3_).

Benzyl (*R*)‐[1‐(4‐methoxyphenyl)‐2‐oxoazetidin‐3‐yl]carbamate (**13**): white solid, 54%, ^1^H NMR (400 MHz, DMSO‐*d*_6_) *δ* 8.10 (d, *J* = 8.4 Hz, 1H, NH), 7.41–7.28 (m, 7H, Ar‐H), 6.99–6.92 (m, 2H, Ar‐H), 5.06 (s, 2H, CH_2_), 4.89–4.83 (m, 1H, CH), 3.90 (t, *J* = 5.6 Hz, 1H, CH_2_), 3.73 (s, 3H, CH_3_), 3.55 (dd, *J* = 5.6, 2.8 Hz, 1H, CH_2_).

Benzyl (*R*)‐{1‐[4‐(dimethylamino)phenyl]‐2‐oxoazetidin‐3‐yl}carbamate (**14**): white solid, 70%, ^1^H NMR (500 MHz, DMSO‐*d*_6_) *δ* 8.09 (d, *J* = 8.5 Hz, 1H, NH), 7.41–7.34 (m, 5H, Ar‐H), 7.22 (d, *J* = 9.0 Hz, 2H, Ar‐H), 6.75 (d, *J* = 9.0 Hz, 2H, Ar‐H), 5.06 (s, 2H, CH_2_), 4.88–4.82 (m, 1H, CH), 3.88 (t, *J* = 5.5 Hz, 1H, CH_2_), 3.51 (dd, *J* = 5.0, 2.5 Hz, 1H, CH_2_), 2.87 (s, 6H, CH_3_).

Benzyl (*R*)‐{2‐oxo‐1‐[4‐(trifluoromethyl)phenyl]azetidin‐3‐yl}carbamate (**15**): white solid, 70%, ^1^H NMR (400 MHz, DMSO‐*d*_6_) *δ* 8.19 (d, *J* = 8.4 Hz, 1H, NH), 7.76 (d, *J* = 8.4 Hz, 2H, Ar‐H), 7.53 (d, *J* = 8.4 Hz, 2H, Ar‐H), 7.41–7.34 (m, 5H, Ar‐H), 5.06 (s, 2H, CH_2_), 4.95–4.89 (m, 1H, CH), 4.01 (t, *J* = 6.0 Hz, 1H, CH_2_), 3.71–3.66 (m, 1H, CH_2_).

#### 4.1.4 General Procedure for the Synthesis of (3*R*)‐ or (3*S*)‐3‐Amino‐*β*‐Lactams 16~21

The 3(*R*)‐ or 3(*S*)‐*β*‐lactams **10**~**15** were dissolved in ethanol (10 mL), combined with 10% palladium on carbon, and stirred under H_2_ atmosphere for 2‐3 h. The mixture was then filtered, and 1 M HCl (1 mL) was added to the filtrate. The filtrate was evaporated to dryness, yielding a pure product as a hydrochloride salt.

#### 4.1.5 General Procedure for the Synthesis of (3*R*)‐ or (3*S*)‐*β*‐Lactam Derivatives 22~29

A solution of Boc‐l‐homophenylalanine (1.0 equiv) in CH_2_Cl_2_ (20 mL) was treated with HOBt (1.1 equiv) and EDCI (1.65 equiv) at 0°C. The reaction mixture was stirred for 30 min at room temperature. Subsequently, (3*R*)‐ or (3*S*)‐3‐amino‐*β*‐lactams **16**~**21** (1.0 equiv) and DIPEA (3.0 equiv) were added. After stirring at room temperature for an additional 3 h, the reaction system was washed with aqueous NaHCO_3_ (30 mL) and brine (30 mL). The dichloromethane layer was dried over anhydrous Na_2_SO_4_ and then evaporated in vacuo. The crude product was purified using flash chromatography (petroleum ether/ethyl acetate = 2:1).

*tert*‐Butyl [(*S*)‐1‐oxo‐1‐{[(*R*)‐2‐oxo‐1‐phenylazetidin‐3‐yl]amino}‐4‐phenylbutan‐2‐yl]carbamate (**22**): white solid, 60%, ^1^H NMR (500 MHz, DMSO‐*d*_6_) *δ* 8.69 (d, *J* = 8.2 Hz, 1H, NH), 7.40–7.33 (m, 4H, Ar‐H), 7.28 (t, *J* = 7.5 Hz, 2H, Ar‐H), 7.19 (dd, *J* = 7.7, 2.6 Hz, 3H, Ar‐H), 7.13–7.08 (m, 2H, Ar‐H), 5.06–4.99 (m, 1H, CH), 3.97–3.87 (m, 2H, CH + CH_2_), 3.56 (dd, *J* = 5.3, 2.8 Hz, 1H, CH_2_), 2.71–2.52 (m, 2H, CH_2_), 1.95–1.71 (m, 2H, CH_2_), 1.41 (s, 9H, CH_3_).

*tert*‐Butyl [(*S*)‐1‐{[(*R*)‐1‐(4‐fluorophenyl)‐2‐oxoazetidin‐3‐yl]amino}‐1‐oxo‐4‐phenylbutan‐2‐yl]carbamate (**24**): white solid, 65%, ^1^H NMR (400 MHz, DMSO‐*d*_6_) *δ* 8.68 (d, *J* = 8.4 Hz, 1H, NH), 7.42–7.36 (m, 2H, Ar‐H), 7.30–7.15 (m, 7H, Ar‐H), 7.12 (d, *J* = 8.0 Hz, 1H, NH), 5.05–4.99 (m, 1H, CH), 3.95–3.87 (m, 2H, CH + CH_2_), 3.55 (dd, *J* = 5.6, 2.8 Hz, 1H, CH_2_), 2.69–2.53 (m, 2H, CH_2_), 1.94–1.73 (m, 2H, CH_2_), 1.41 (s, 9H, CH_3_).

*tert*‐Butyl [(*S*)‐1‐{[(*S*)‐1‐(4‐fluorophenyl)‐2‐oxoazetidin‐3‐yl]amino}‐1‐oxo‐4‐phenylbutan‐2‐yl]carbamate (**25**): white solid, 74%, ^1^H NMR (400 MHz, CDCl_3_) *δ* 7.35–7.27 (m, 5H, Ar‐H), 7.25–7.17 (m, 3H, Ar‐H + NH), 7.09–7.02 (m, 2H, Ar‐H), 5.19 (d, *J* = 8.0 Hz, 1H, NH), 5.11–5.04 (m, 1H, CH), 4.12–4.21 (m, 1H, CH), 3.95 (t, *J* = 5.6 Hz, 1H, CH_2_), 3.60 (dd, *J* = 5.6, 2.4 Hz, 1H, CH_2_), 2.78–2.66 (m, 2H, CH_2_), 2.24–2.14 (m, 1H, CH_2_), 2.02–1.90 (m, 1H, CH_2_), 1.47 (s, 9H, CH_3_).

*tert*‐Butyl [(*S*)‐1‐oxo‐1‐{[(*R*)‐2‐oxo‐1‐(p‐tolyl)azetidin‐3‐yl]amino}‐4‐phenylbutan‐2‐yl]carbamate (**26**): white solid, 68%, ^1^H NMR (400 MHz, DMSO‐*d*_6_) *δ* 8.70 (d, *J* = 8.4 Hz, 1H, NH), 7.30–7.23 (m, 4H, Ar‐H), 7.21–7.15 (m, 5H, Ar‐H), 7.12 (d, *J* = 8.0 Hz, 1H, NH), 5.06–4.99 (m, 1H, CH), 3.96–3.86 (m, 2H, CH + CH_2_), 3.52 (dd, *J* = 5.2, 2.4 Hz, 1H, CH_2_), 2.69–2.53 (m, 2H, CH_2_), 2.27 (s, 3H, CH_3_), 1.96–1.74 (m, 2H, CH_2_), 1.41 (s, 9H, CH_3_).

*tert*‐Butyl [(*S*)‐1‐{[(*R*)‐1‐(4‐methoxyphenyl)‐2‐oxoazetidin‐3‐yl]amino}‐1‐oxo‐4‐phenylbutan‐2‐yl]carbamate (**27**): white solid, 63%, ^1^H NMR (400 MHz, DMSO‐*d*_6_) *δ* 8.68 (d, *J* = 8.4 Hz, 1H, NH), 7.33–7.25 (m, 4H, Ar‐H), 7.21–7.15 (m, 3H, Ar‐H), 7.11 (d, *J* = 7.6 Hz, 1H, NH), 6.98–6.92 (m, 2H, Ar‐H), 5.05–4.98 (m, 1H, CH), 3.94–3.84 (m, 2H, CH + CH_2_), 3.73 (s, 3H, CH_3_), 3.50 (dd, *J* = 5.2, 2.8 Hz, 1H, CH_2_), 2.69–2.53 (m, 2H, CH_2_), 1.95–1.73 (m, 2H, CH_2_), 1.41 (s, 9H, CH_3_).

*tert*‐Butyl [(*S*)‐1‐{[(*R*)‐1‐[4‐(dimethylamino)phenyl]‐2‐oxoazetidin‐3‐yl]amino}‐1‐oxo‐4‐phenylbutan‐2‐yl]carbamate (**28**): white solid, 70%, ^1^H NMR (500 MHz, DMSO‐*d*_6_) *δ* 8.68 (d, *J* = 8.0 Hz, 1H, NH), 7.27 (t, *J* = 7.5 Hz, 2H, Ar‐H), 7.24–7.15 (m, 5H, Ar‐H), 7.09 (d, *J* = 7.9 Hz, 1H, NH), 6.74 (d, *J* = 9.0 Hz, 2H, Ar‐H), 5.03–4.98 (m, 1H, CH), 3.95–3.89 (m, 1H, CH), 3.84 (t, *J* = 5.5 Hz, 1H, CH_2_), 3.49–3.44 (m, 1H, CH_2_), 2.86 (s, 6H, CH_3_), 2.68–2.52 (m, 2H, CH_2_), 1.94–1.74 (m, 2H, CH_2_), 1.41 (s, 9H, CH_3_).

*tert*‐Butyl [(*S*)‐1‐oxo‐1‐{[(*R*)‐2‐oxo‐1‐[4‐(trifluoromethyl)phenyl]azetidin‐3‐yl]amino}‐4‐phenylbutan‐2‐yl]carbamate (**29**): white solid, 59%, ^1^H NMR (400 MHz, DMSO‐*d*_6_) *δ* 8.70 (d, *J* = 8.0 Hz, 1H, NH), 7.75 (d, *J* = 8.8 Hz, 2H, Ar‐H), 7.53 (d, *J* = 8.4 Hz, 2H, Ar‐H), 7.32–7.24 (m, 2H, Ar‐H), 7.22–7.12 (m, 4H, Ar‐H + NH), 5.09–5.01 (m, 1H, CH), 3.98 (t, *J* = 6.0 Hz, 1H, CH_2_), 3.95–3.88 (m, 1H, CH), 3.67–3.60 (m, 1H, CH_2_), 2.69–2.53 (m, 2H, CH_2_), 1.97–1.75 (m, 2H, CH_2_), 1.41 (s, 9H, CH_3_).

#### 4.1.6 General Procedure for the Synthesis of Deprotected (3*R*)‐ or (3*S*)‐*β*‐Lactam Derivatives 30~37

To a solution of (3*R*)‐ or (3*S*)‐*β*‐lactam derivatives **22**~**29** in CH_2_Cl_2_ (20 mL), the trifluoroacetic acid (TFA, 10 mL) was added at 0°C. The reaction mixture was then warmed to room temperature and stirred for 1–2 h. The solvent and TFA were evaporated under reduced pressure, and ether (20 mL) was added to precipitate a white solid. The resulting solid was filtered and dried and was subsequently used in the next step without further purification.

#### 4.1.7 General Procedure for the Synthesis of Dipeptides 38~50

Boc‐protected amino acids (1.0 equiv) were dissolved in CH_2_Cl_2_ (20 mL), and HOBt (1.1 equiv), along with EDCI (1.65 equiv), was added at 0°C. The reaction was allowed to proceed for 30 min at room temperature. Subsequently, deprotected (3*R*)‐ or (3*S*)‐*β*‐lactam derivatives **30**~**37** (1.0 equiv) and DIPEA (3.0 equiv) were added. After stirring at room temperature for an additional 3 h, the reaction mixture was washed with aqueous NaHCO_3_ (30 mL) and brine (30 mL). The dichloromethane layer was dried over anhydrous Na_2_SO_4_ and then evaporated in vacuo. The crude product was purified using flash chromatography (dichloromethane/ethyl acetate = 3:1).

*tert*‐Butyl [(*S*)‐4‐(neopentylamino)‐1,4‐dioxo‐1‐{[(*S*)‐1‐oxo‐1‐{[(*R*)‐2‐oxo‐1‐phenylazetidin‐3‐yl]amino}‐4‐phenylbutan‐2‐yl]amino}butan‐2‐yl]carbamate (**38**): white solid, 64%, ^1^H NMR (500 MHz, DMSO‐*d*_6_) *δ* 8.77 (d, *J* = 8.4 Hz, 1H, NH), 8.22 (d, *J* = 8.1 Hz, 1H, NH), 7.85 (t, *J* = 6.1 Hz, 1H, NH), 7.39–7.33 (m, 4H, Ar‐H), 7.26 (t, *J* = 7.5 Hz, 2H, Ar‐H), 7.17 (t, *J* = 5.6 Hz, 3H, Ar‐H), 7.09 (ddd, *J* = 13.2, 5.9, 4.1 Hz, 2H, Ar‐H + NH), 5.10–5.04 (m, 1H, CH), 4.26 (q, *J* = 7.3 Hz, 1H, CH), 4.21–4.13 (m, 1H, CH), 3.94 (t, *J* = 5.7 Hz, 1H, CH_2_), 3.58 (dd, *J* = 5.5, 2.9 Hz, 1H, CH_2_), 2.82 (dd, *J* = 13.1, 6.4 Hz, 1H, CH_2_), 2.72 (dd, *J* = 13.1, 6.0 Hz, 1H, CH_2_), 2.68–2.60 (m, 2H, CH_2_), 2.59–2.52 (m, 2H, CH_2_), 2.12–2.01 (m, 1H, CH_2_), 1.88–1.78 (m, 1H, CH_2_), 1.37 (s, 9H, CH_3_), 0.73 (s, 9H, CH_3_).

*tert*‐Butyl {(*S*)‐4‐(neopentylamino)‐1,4‐dioxo‐1‐[((*S*)‐1‐oxo‐1‐{[(*S*)‐2‐oxo‐1‐phenylazetidin‐3‐yl]amino}‐4‐phenylbutan‐2‐yl)amino]butan‐2‐yl}carbamate (**39**): white solid, 67%, ^1^H NMR (500 MHz, DMSO‐*d*_6_) *δ* 8.80 (d, *J* = 8.6 Hz, 1H, NH), 8.27 (d, *J* = 7.8 Hz, 1H, NH), 7.88 (t, *J* = 6.1 Hz, 1H, NH), 7.38–7.32 (m, 4H, Ar‐H), 7.26 (t, *J* = 7.5 Hz, 2H, Ar‐H), 7.20–7.14 (m, 3H, Ar‐H), 7.11–7.06 (m, 1H, Ar‐H), 7.01 (d, *J* = 7.7 Hz, 1H, NH), 5.15–5.07 (m, 1H, CH), 4.25 (dd, *J* = 14.3, 7.3 Hz, 1H, CH), 4.15–4.07 (m, 1H, CH), 3.93 (t, *J* = 5.6 Hz, 1H, CH_2_), 3.53 (dd, *J* = 5.4, 2.8 Hz, 1H, CH_2_), 2.78 (dd, *J* = 13.1, 6.4 Hz, 1H, CH_2_), 2.73–2.61 (m, 3H, CH_2_), 2.60–2.52 (m, 2H, CH_2_), 2.13–2.01 (m, 1H, CH_2_), 1.90–1.80 (m, 1H, CH_2_), 1.36 (s, 9H, CH_3_), 0.70 (s, 9H, CH_3_).

*tert*‐Butyl [(*S*)‐1‐{[(*S*)‐1‐{[(*R*)‐1‐(4‐fluorophenyl)‐2‐oxoazetidin‐3‐yl]amino}‐1‐oxo‐4‐phenylbutan‐2‐yl]amino}‐4‐(neopentylamino)‐1,4‐dioxobutan‐2‐yl]carbamate (**40**): white solid, 53%, ^1^H NMR (400 MHz, DMSO‐*d*_6_) *δ* 8.80 (d, *J* = 8.8 Hz, 1H, NH), 8.27 (d, *J* = 7.6 Hz, 1H, NH), 7.88 (t, *J* = 6.0 Hz, 1H, NH), 7.41–7.34 (m, 2H, Ar‐H), 7.30–7.21 (m, 4H, Ar‐H), 7.20–7.14 (m, 3H, Ar‐H), 7.00 (d, *J* = 7.6 Hz, 1H, NH), 5.16–5.08 (m, 1H, CH), 4.25 (dd, *J* = 14.4, 7.2 Hz, 1H, CH), 4.16–4.09 (m, 1H, CH), 3.92 (t, *J* = 5.6 Hz, 1H, CH_2_), 3.52 (dd, *J* = 5.2, 2.8 Hz, 1H, CH_2_), 2.77 (dd, *J* = 13.2, 6.4 Hz, 1H, CH_2_), 2.73–2.60 (m, 3H, CH_2_), 2.59–2.52 (m, 2H, CH_2_), 2.13–2.03 (m, 1H, CH_2_), 1.93–1.78 (m, 1H, CH_2_), 1.36 (s, 9H, CH_3_), 0.70 (s, 9H, CH_3_).

*tert*‐Butyl [(*S*)‐1‐{[(*S*)‐1‐{[(*S*)‐1‐(4‐fluorophenyl)‐2‐oxoazetidin‐3‐yl]amino}‐1‐oxo‐4‐phenylbutan‐2‐yl]amino}‐4‐(neopentylamino)‐1,4‐dioxobutan‐2‐yl]carbamate (**41**): white solid, 59%, ^1^H NMR (400 MHz, DMSO‐*d*_6_) *δ* 8.79 (d, *J* = 8.4 Hz, 1H, NH), 8.24 (d, *J* = 8.0 Hz, 1H, NH), 7.87 (t, *J* = 6.0 Hz, 1H, NH), 7.43–7.35 (m, 2H, Ar‐H), 7.32–7.23 (m, 4H, Ar‐H), 7.23–7.15 (m, 3H, Ar‐H), 7.08 (d, *J* = 8.0 Hz, 1H, NH), 5.14–5.06 (m, 1H, CH), 4.28 (dd, *J* = 14.8, 7.2 Hz, 1H, CH), 4.23–4.15 (m, 1H, CH), 3.96 (t, *J* = 5.6 Hz, 1H, CH_2_), 3.60 (dd, *J* = 5.6, 2.8 Hz, 1H, CH_2_), 2.84 (dd, *J* = 13.2, 6.4 Hz, 1H, CH_2_), 2.74 (dd, *J* = 13.2, 6.4 Hz, 1H, CH_2_), 2.71–2.62 (m, 2H, CH_2_), 2.62–2.54 (m, 2H, CH_2_), 2.13–2.04 (m, 1H, CH_2_), 1.94–1.81 (m, 1H, CH_2_), 1.39 (s, 9H, CH_3_), 0.75 (s, 9H, CH_3_).

*tert*‐Butyl [(*S*)‐1‐{[(*S*)‐1‐{[(*R*)‐1‐(4‐fluorophenyl)‐2‐oxoazetidin‐3‐yl]amino}‐1‐oxo‐4‐phenylbutan‐2‐yl]amino}‐1‐oxopropan‐2‐yl]carbamate (**42**): white solid, 73%, ^1^H NMR (400 MHz, DMSO‐*d*_6_) *δ* 8.66 (d, *J* = 8.0 Hz, 1H, NH), 7.99 (d, *J* = 7.9 Hz, 1H, NH), 7.42–7.35 (m, 2H, Ar‐H), 7.31–7.20 (m, 4H, Ar‐H), 7.20–7.14 (m, 3H, Ar‐H), 7.07 (d, *J* = 6.8 Hz, 1H, NH), 5.04–4.97 (m, 1H, CH), 4.27–4.19 (m, 1H, CH), 4.05–3.96 (m, 1H, CH), 3.92 (t, *J* = 5.6 Hz, 1H, CH_2_), 3.56 (dd, *J* = 5.2, 2.8 Hz, 1H, CH_2_), 2.69–2.52 (m, 2H, CH_2_), 2.01–1.81 (m, 2H, CH_2_), 1.35 (s, 9H, CH_3_), 1.20 (d, *J* = 7.2 Hz, 3H, CH_3_).

*tert*‐Butyl [(*S*)‐1‐{[(*S*)‐1‐{[(*R*)‐1‐(4‐fluorophenyl)‐2‐oxoazetidin‐3‐yl]amino}‐1‐oxo‐4‐phenylbutan‐2‐yl]amino}‐3‐methyl‐1‐oxobutan‐2‐yl]carbamate (**43**): white solid, 65%, ^1^H NMR (400 MHz, DMSO‐*d*_6_) *δ* 8.71 (d, *J* = 8.0 Hz, 1H, NH), 8.00 (d, *J* = 8.0 Hz, 1H, NH), 7.42–7.35 (m, 2H, Ar‐H), 7.31–7.21 (m, 4H, Ar‐H), 7.20–7.15 (m, 3H, Ar‐H), 6.85 (d, *J* = 8.8 Hz, 1H, NH), 5.09–5.00 (m, 1H, CH), 4.33–4.24 (m, 1H, CH), 3.94 (t, *J* = 5.6 Hz, 1H, CH_2_), 3.82 (t, *J* = 7.6 Hz, 1H, CH), 3.54 (dd, *J* = 5.6, 2.8 Hz, 1H, CH_2_), 2.70–2.54 (m, 2H, CH_2_), 2.03–1.80 (m, 2H, CH_2_), 1.38 (s, 9H, CH_3_), 0.87 (d, *J* = 6.4 Hz, 3H, CH_3_), 0.84 (d, *J* = 6.8 Hz, 3H, CH_3_).

*tert*‐Butyl [(*S*)‐1‐{[(*S*)‐1‐{[(*R*)‐1‐(4‐fluorophenyl)‐2‐oxoazetidin‐3‐yl]amino}‐1‐oxo‐4‐phenylbutan‐2‐yl]amino}‐1‐oxo‐4‐phenylbutan‐2‐yl]carbamate (**44**): white solid, 65%, ^1^H NMR (500 MHz, DMSO‐*d*_6_) *δ* 8.70 (d, *J* = 8.0 Hz, 1H, NH), 8.06 (d, *J* = 7.8 Hz, 1H, NH), 7.4–7.33 (m, 2H, Ar‐H), 7.28–7.22 (m, 6H, Ar‐H), 7.21–7.15 (m, 7H, Ar‐H + NH), 5.03–4.95 (m, 1H, CH), 4.25 (dd, *J* = 12.7, 8.5 Hz, 1H, CH), 3.98 (dd, *J* = 13.2, 7.9 Hz, 1H, CH), 3.91 (t, *J* = 5.6 Hz, 1H, CH_2_), 3.54 (dd, *J* = 5.4, 2.7 Hz, 1H, CH_2_), 2.68–2.53 (m, 4H, CH_2_), 1.98–1.80 (m, 4H, CH_2_), 1.38 (s, 9H, CH_3_).

*tert*‐Butyl [(*S*)‐1‐{[(*S*)‐1‐{[(*R*)‐1‐(4‐fluorophenyl)‐2‐oxoazetidin‐3‐yl]amino}‐1‐oxo‐4‐phenylbutan‐2‐yl]amino}‐1‐oxo‐3‐phenylpropan‐2‐yl]carbamate (**45**): white solid, 69%, ^1^H NMR (500 MHz, DMSO‐*d*_6_) *δ* 8.67 (d, *J* = 8.1 Hz, 1H, NH), 8.14 (d, *J* = 7.9 Hz, 1H, NH), 7.42–7.37 (m, 2H, Ar‐H), 7.28–7.23 (m, 7H, Ar‐H), 7.22–7.16 (m, 5H, Ar‐H), 7.02 (d, *J* = 8.4 Hz, 1H, NH), 5.03–4.98 (m, 1H, CH), 4.30–4.20 (m, 2H, CH), 3.93 (t, *J* = 5.6 Hz, 1H, CH_2_), 3.56 (dd, *J* = 5.4, 2.7 Hz, 1H, CH_2_), 3.02 (dd, *J* = 13.8, 4.0 Hz, 1H, CH_2_), 2.77 (dd, *J* = 13.8, 10.5 Hz, 1H, CH_2_), 2.67–2.55 (m, 2H, CH_2_), 2.00–1.83 (m, 2H, CH_2_), 1.30 (s, 9H, CH_3_).

*tert*‐Butyl [(*S*)‐1‐{[(*S*)‐1‐{[(*R*)‐1‐(4‐fluorophenyl)‐2‐oxoazetidin‐3‐yl]amino}‐1‐oxo‐4‐phenylbutan‐2‐yl]amino}‐4‐(methylthio)‐1‐oxobutan‐2‐yl]carbamate (**46**): white solid, 63%, ^1^H NMR (500 MHz, DMSO‐*d*_6_) *δ* 8.63 (d, *J* = 8.0 Hz, 1H, NH), 8.22 (d, *J* = 8.0 Hz, 1H, NH), 7.40–7.35 (m, 2H, Ar‐H), 7.30–7.19 (m, 5H, Ar‐H), 7.18–7.14 (m, 3H, Ar‐H), 7.12 (d, *J* = 7.0 Hz, 1H, NH), 5.06–5.00 (m, 1H, CH), 4.28‐4.21 (m, 1H, CH), 4.07–4.00 (m, 1H, CH), 3.95 (t, *J* = 5.5 Hz, 1H, CH_2_), 3.54 (dd, *J* = 5.5, 2.5 Hz, 1H, CH_2_), 2.64–2.56 (m, 1H, CH_2_), 2.55–2.41 (m, 3H, CH_2_), 2.03 (s, 3H, CH_3_), 2.01–1.98 (m, 1H, CH_2_), 1.92–1.78 (m, 3H, CH_2_), 1.33 (s, 9H, CH_3_).

*tert*‐Butyl [(*S*)‐4‐(neopentylamino)‐1,4‐dioxo‐1‐{[(*S*)‐1‐oxo‐1‐{[(*R*)‐2‐oxo‐1‐(p‐tolyl)azetidin‐3‐yl]amino}‐4‐phenylbutan‐2‐yl]amino}butan‐2‐yl]carbamate (**47**): white solid, 62%, ^1^H NMR (500 MHz, DMSO‐*d*_6_) *δ* 8.81 (d, *J* = 8.5 Hz, 1H, NH), 8.28 (d, *J* = 8.0 Hz, 1H, NH), 7.89 (t, *J* = 6.0 Hz, 1H, NH), 7.39–7.33 (m, 4H, Ar‐H), 7.27 (t, *J* = 7.5 Hz, 2H, Ar‐H), 7.22–7.16 (m, 3H, Ar‐H), 7.12–7.08 (m, 1H, Ar‐H), 7.02 (d, *J* = 7.5 Hz, 1H, NH), 5.15–5.09 (m, 1H, CH), 4.26 (dd, *J* = 14.0, 7.0 Hz, 1H, CH), 4.17–4.10 (m, 1H, CH), 3.94 (t, *J* = 5.5 Hz, 1H, CH_2_), 3.54 (dd, *J* = 5.0, 2.5 Hz, 1H, CH_2_), 2.79 (dd, *J* = 13.5, 6.5 Hz, 1H, CH_2_), 2.74–2.62 (m, 3H, CH_2_), 2.61–2.53 (m, 2H, CH_2_), 2.27 (s, 3H, CH_3_), 2.14–2.03 (m, 1H, CH_2_), 1.92–1.82 (m, 1H, CH_2_), 1.37 (s, 9H, CH_3_), 0.71 (s, 9H, CH_3_).

*tert*‐Butyl [(*S*)‐1‐{[(*S*)‐1‐{[(*R*)‐1‐[4‐(dimethylamino)phenyl]‐2‐oxoazetidin‐3‐yl]amino}‐1‐oxo‐4‐phenylbutan‐2‐yl]amino}‐4‐(neopentylamino)‐1,4‐dioxobutan‐2‐yl]carbamate (**48**): white solid, 65%, ^1^H NMR (400 MHz, DMSO‐*d*_6_) *δ* 8.77 (d, *J* = 8.4 Hz, 1H, NH), 8.25 (d, *J* = 8.0 Hz, 1H, NH), 7.86 (t, *J* = 6.0 Hz, 1H, NH), 7.29–7.23 (m, 2H, Ar‐H), 7.22–7.13 (m, 5H, Ar‐H), 7.01 (d, *J* = 7.6 Hz, 1H, NH), 6.72 (d, *J* = 8.8 Hz, 2H, Ar‐H), 5.10–5.04 (m, 1H, CH), 4.25 (dd, *J* = 14.4, 7.2 Hz, 1H, CH), 4.16–4.07 (m, 1H, CH), 3.85 (t, *J* = 5.6 Hz, 1H, CH_2_), 3.45 (dd, *J* = 4.8, 2.4 Hz, 1H, CH_2_), 3.35 (s, 6H, CH_3_), 2.79 (dd, *J* = 13.2, 6.4 Hz, 1H, CH_2_), 2.74–2.60 (m, 3H, CH_2_), 2.60–2.53 (m, 2H, CH_2_), 2.10–2.01 (m, 1H, CH_2_), 1.93–1.79 (m, 1H, CH_2_), 1.36 (s, 9H, CH_3_), 0.71 (s, 9H, CH_3_).

*tert*‐Butyl [(*S*)‐1‐{[(*S*)‐1‐{[(*R*)‐1‐(4‐methoxyphenyl)‐2‐oxoazetidin‐3‐yl]amino}‐1‐oxo‐4‐phenylbutan‐2‐yl]amino}‐4‐(neopentylamino)‐1,4‐dioxobutan‐2‐yl]carbamate (**49**): white solid, 53%, ^1^H NMR (500 MHz, DMSO‐*d*_6_) *δ* 8.79 (d, *J* = 8.5 Hz, 1H, NH), 8.26 (d, *J* = 7.5 Hz, 1H, NH), 7.87 (t, *J* = 6.0 Hz, 1H, NH), 7.33–7.24 (m, 4H, Ar‐H), 7.22–7.15 (m, 3H), 7.01 (d, *J* = 7.5 Hz, 1H, NH), 6.94 (d, *J* = 9.0 Hz, 2H, Ar‐H), 5.13–5.08 (m, 1H, CH), 4.26 (dd, *J* = 14.0, 7.0 Hz, 1H, CH), 4.17–4.09 (m, 1H, CH), 3.90 (t, *J* = 5.5 Hz, 1H, CH_2_), 3.73 (s, 3H, CH_3_), 3.50 (dd, *J* = 5.0, 2.5 Hz, 1H, CH_2_), 2.79 (dd, *J* = 13.0, 6.5 Hz, 1H, CH_2_), 2.75–2.62 (m, 3H, CH_2_), 2.60–2.53 (m, 2H, CH_2_), 2.11–2.01 (m, 1H, CH_2_), 1.93–1.76 (m, 1H, CH_2_), 1.37 (s, 9H, CH_3_), 0.72 (s, 9H, CH_3_).

*tert*‐Butyl [(*S*)‐4‐(neopentylamino)‐1,4‐dioxo‐1‐{[(*S*)‐1‐oxo‐1‐{[(*R*)‐2‐oxo‐1‐[4‐(trifluoromethyl)phenyl]azetidin‐3‐yl]amino}‐4‐phenylbutan‐2‐yl]amino}butan‐2‐yl]carbamate (**50**): white solid, 55%, ^1^H NMR (400 MHz, DMSO‐*d*_6_) *δ* 8.85 (d, *J* = 8.8 Hz, 1H, NH), 8.32 (d, *J* = 7.6 Hz, 1H, NH), 7.91 (t, *J* = 6.0 Hz, 1H, NH), 7.73 (d, *J* = 8.8 Hz, 2H, Ar‐H), 7.52 (d, *J* = 8.8 Hz, 2H, Ar‐H), 7.31–7.21 (m, 5H, Ar‐H), 7.06 (d, *J* = 8.0 Hz, 1H, NH), 5.21–5.14 (m, 1H, CH), 4.48 (dd, *J* = 13.6, 7.2 Hz, 1H, CH), 4.13–4.07 (m, 1H, CH), 4.02–3.98 (m, 1H, CH_2_), 3.55–3.47 (m, 1H, CH_2_), 2.73 (dd, *J* = 12.8, 6.4 Hz, 1H, CH_2_), 2.69–2.61 (m, 3H, CH_2_), 2.60–2.55 (m, 2H, CH_2_), 2.12–2.03 (m, 1H, CH_2_), 1.89–1.82 (m, 1H, CH_2_), 1.37 (s, 9H, CH_3_), 0.66 (s, 9H, CH_3_).

#### 4.1.8 General Procedure for the Synthesis of Deprotected Dipeptides 51~63

To a solution of dipeptides **38**~**50** in CH_2_Cl_2_ (20 mL), the trifluoroacetic acid (TFA, 10 mL) was added at 0°C. The reaction mixture was then warmed to room temperature and stirred for 1–2 h. The solvent and TFA were evaporated under reduced pressure, and ether (20 mL) was added to precipitate a white solid. The resulting solid was filtered and dried and was subsequently used in the next step without further purification.

#### 4.1.9 General Procedure for the Synthesis of Target Compounds 64~79

Nicotinic acid or 2‐Pyrazinecarboxylic acid (1.0 equiv) was dissolved in CH_2_Cl_2_ (20 mL), and HOBt (1.1 equiv), along with EDCI (1.65 equiv), was added at 0°C. The reaction was allowed to proceed for 30 min at room temperature. Subsequently, deprotected dipeptides **51**~**63** (1.0 equiv) and DIPEA (3.0 equiv) were added. After stirring at room temperature for an additional 3 h, the reaction mixture was washed with aqueous NaHCO_3_ (30 mL) and brine (30 mL). The dichloromethane layer was dried over anhydrous Na_2_SO_4_ and then evaporated in vacuo. The crude product was purified using flash chromatography (dichloromethane/ethyl acetate = 1:1).

(*S*)‐*N* ^4^‐Neopentyl‐2‐(nicotinamido)‐*N* ^1^‐[(S)‐1‐oxo‐1‐{[(*R*)‐2‐oxo‐1‐phenylazetidin‐3‐yl]amino}‐4‐phenylbutan‐2‐yl]succinimide (**64**): white solid, 64%, ^1^H NMR (500 MHz, DMSO‐*d*_6_) *δ* 9.03 (d, *J* = 1.6 Hz, 1H, pyridine‐H), 8.96 (d, *J* = 7.6 Hz, 1H, NH), 8.78 (d, *J* = 8.4 Hz, 1H, NH), 8.71 (dd, *J* = 4.8, 1.6 Hz, 1H, pyridine‐H), 8.43 (d, *J* = 8.0 Hz, 1H, NH), 8.24–8.19 (m, 1H, pyridine‐H), 7.93 (t, *J* = 6.2 Hz, 1H, NH), 7.51 (dd, *J* = 7.9, 4.8 Hz, 1H, pyridine‐H), 7.40–7.32 (m, 4H, Ar‐H), 7.26–7.21 (m, 2H, Ar‐H), 7.19–7.13 (m, 3H, Ar‐H), 7.12–7.08 (m, 1H, Ar‐H), 5.12–5.07 (m, 1H, CH), 4.83 (q, *J* = 7.3 Hz, 1H, CH), 4.25–4.15 (m, 1H, CH), 3.95 (t, *J* = 5.7 Hz, 1H, CH_2_), 3.60 (dd, *J* = 5.5, 2.9 Hz, 1H, CH_2_), 2.89–2.80 (m, 2H, CH_2_), 2.78–2.70 (m, 2H, CH_2_), 2.69–2.62 (m, 1H, CH_2_), 2.59–2.52 (m, 1H, CH_2_), 2.13–2.03 (m, 1H, CH_2_), 1.92–1.82 (m, 1H, CH_2_), 0.73 (s, 9H, CH_3_). ^13^C NMR (125 MHz, DMSO‐*d*_6_) *δ* 172.22, 171.56, 170.28, 165.38, 164.97, 152.52, 149.07, 141.65, 138.59, 135.67, 129.95, 129.68, 128.90, 128.72, 126.31, 124.13, 123.87, 116.63, 55.11, 52.68, 51.35, 50.13, 46.81, 37.64, 33.44, 32.19, 31.70, 27.52. HPLC purity: 100.0%. HRAM‐MS (ESI) *m*/*z*: [M+H]^+^ calcd for C_34_H_41_N_6_O_5_ 613.3138; found 613.3127.

(*S*)‐*N* ^4^‐Neopentyl‐2‐(nicotinamido)‐*N* ^1^‐[(*S*)‐1‐oxo‐1‐{[(*S*)‐2‐oxo‐1‐phenylazetidin‐3‐yl]amino}‐4‐phenylbutan‐2‐yl]succinimide (**65**): white solid, 59%, ^1^H NMR (500 MHz, DMSO‐*d*_6_) *δ* 9.02 (d, *J* = 1.6 Hz, 1H, pyridine‐H), 8.90 (d, *J* = 7.6 Hz, 1H, NH), 8.80 (d, *J* = 8.6 Hz, 1H, NH), 8.71 (dd, *J* = 4.8, 1.6 Hz, 1H, pyridine‐H), 8.47 (d, *J* = 7.8 Hz, 1H, NH), 8.24–8.14 (m, 1H, pyridine‐H), 7.92 (t, *J* = 6.2 Hz, 1H, NH), 7.55–7.47 (m, 1H, pyridine‐H), 7.39–7.34 (m, 4H, Ar‐H), 7.26–7.20 (m, 2H, Ar‐H), 7.16 (dd, *J* = 6.8, 4.3 Hz, 3H, Ar‐H), 7.10 (ddd, *J* = 8.5, 6.4, 2.2 Hz, 1H, Ar‐H), 5.12 (ddd, *J* = 8.6, 5.8, 2.8 Hz, 1H, CH), 4.82 (q, *J* = 7.2 Hz, 1H, CH), 4.16 (ddd, *J* = 11.6, 7.9, 4.0 Hz, 1H, CH), 3.95 (t, *J* = 5.6 Hz, 1H, CH_2_), 3.56 (dd, *J* = 5.5, 2.8 Hz, 1H, CH_2_), 2.84 (ddd, *J* = 10.9, 6.7, 3.2 Hz, 2H, CH_2_), 2.76–2.62 (m, 3H, CH_2_), 2.56 (dt, *J* = 11.3, 7.2 Hz, 1H, CH_2_), 2.13–1.83 (m, 2H, CH_2_), 0.70 (s, 9H, CH_3_). ^13^C NMR (125 MHz, DMSO‐*d*_6_) *δ* 172.16, 171.47, 170.30, 165.41, 164.95, 152.51, 149.09, 141.72, 138.59, 135.67, 129.95, 129.68, 128.88, 128.72, 126.30, 124.11, 123.87, 116.61, 55.25, 52.52, 51.39, 50.16, 46.54, 37.62, 33.50, 32.24, 31.69, 27.57. HPLC purity: 100.0%. HRAM‐MS (ESI) *m*/*z*: [M+H]^+^ calcd for C_34_H_41_N_6_O_5_ 613.3138; found 613.3125.

(*S*)‐*N* ^1^‐[(*S*)‐1‐{[(*R*)‐1‐(4‐Fluorophenyl)‐2‐oxoazetidin‐3‐yl]amino}‐1‐oxo‐4‐phenylbutan‐2‐yl]‐*N* ^4^‐neopentyl‐2‐(nicotinamido)succinimide (**66**): white solid, 75%, ^1^H NMR (500 MHz, DMSO‐*d*_6_) *δ* 9.02 (d, *J* = 1.5 Hz, 1H, pyridine‐H), 8.90 (d, *J* = 7.5 Hz, 1H, NH), 8.79 (d, *J* = 8.5 Hz, 1H, NH), 8.71 (dd, *J* = 4.5, 1.5 Hz, 1H, pyridine‐H), 8.46 (d, *J* = 7.5 Hz, 1H, NH), 8.23–8.15 (m, 1H, pyridine‐H), 7.92 (t, *J* = 6.5 Hz, 1H, NH), 7.53–7.47 (m, 1H, pyridine‐H), 7.39–7.33 (m, 4H, Ar‐H), 7.26–7.20 (m, 2H, Ar‐H), 7.18–7.13 (m, 3H, Ar‐H), 7.12–7.07 (m, 1H, Ar‐H), 5.14–5.10 (m, 1H, CH), 4.82 (q, *J* = 7.0 Hz, 1H, CH), 4.19–4.12 (m, 1H, CH), 3.94 (t, *J* = 6.0 Hz, 1H, CH_2_), 3.55 (dd, *J* = 5.5, 3.0 Hz, 1H, CH_2_), 2.87–2.81 (m, 2H, CH_2_), 2.75–2.69 (m, 2H, CH_2_), 2.69–2.64 (m, 1H, CH_2_), 2.60–2.54 (m, 1H, CH_2_), 2.13–2.03 (m, 1H, CH_2_), 1.93–1.83 (m, 1H, CH_2_), 0.70 (s, 9H, CH_3_). ^13^C NMR (125 MHz, DMSO‐*d*_6_) *δ* 172.22, 171.56, 170.28, 165.38, 164.97, 152.52, 149.07, 141.65, 138.59, 135.67, 129.95, 129.68, 128.90, 128.72, 126.31, 124.13, 123.87, 116.63, 55.11, 52.68, 51.35, 50.13, 46.81, 37.64, 33.44, 32.19, 31.70, 27.52. HPLC purity: 100.0%. HRAM‐MS (ESI) *m*/*z*: [M+H]^+^ calcd for C_34_H_40_N_6_O_5_ 631.3044; found 631.3040.

(*S*)‐*N* ^1^‐[(*S*)‐1‐{[(*S*)‐1‐(4‐Fluorophenyl)‐2‐oxoazetidin‐3‐yl]amino}‐1‐oxo‐4‐phenylbutan‐2‐yl]‐*N* ^4^‐neopentyl‐2‐(nicotinamido)succinimide (**67**): white solid, 78%, ^1^H NMR (500 MHz, DMSO‐*d*_6_) *δ* 9.08–9.02 (m, 2H, pyridine‐H + NH), 8.84 (d, *J* = 8.5 Hz, 1H, NH), 8.71 (dd, *J* = 5.0, 1.5 Hz, 1H, pyridine‐H), 8.45 (d, *J* = 8.0 Hz, 1H, NH), 8.28–8.23 (m, 1H, pyridine‐H), 8.05 (t, *J* = 6.5 Hz, 1H, NH), 7.50 (dd, *J* = 7.5, 5.0 Hz, 1H, pyridine‐H), 7.42–7.36 (m, 2H, Ar‐H), 7.26–7.20 (m, 4H, Ar‐H), 7.19–7.13 (m, 3H, Ar‐H), 5.11–5.05 (m, 1H, CH), 4.83 (q, *J* = 7.5 Hz, 1H, CH), 4.26–4.15 (m, 1H, CH), 3.95 (t, *J* = 6.0 Hz, 1H, CH_2_), 3.60 (dd, *J* = 5.0, 2.5 Hz, 1H, CH_2_), 2.90–2.84 (m, 1H, CH_2_), 2.84–2.78 (m, 2H, CH_2_), 2.78–2.73 (m, 1H, CH_2_), 2.69–2.62 (m, 1H, CH_2_), 2.60–2.53 (m, 1H, CH_2_), 2.11–2.02 (m, 1H, CH_2_), 1.93–1.83 (m, 1H, CH_2_), 0.73 (s, 9H, CH_3_). ^13^C NMR (125 MHz, DMSO‐*d*_6_) *δ* 172.20, 171.46, 170.33, 165.40, 164.76, 157.71, 152.49, 149.15, 141.75, 135.71, 135.22, 129.90, 128.88, 128.71, 126.28, 123.83, 118.26 (d, *J* = 7.8 Hz), 116.42 (d, *J* = 22.6 Hz), 55.47, 52.60, 51.58, 50.17, 46.79, 37.67, 33.59, 32.24, 31.70, 27.58. HPLC purity: 100.0%. HRAM‐MS (ESI) *m*/*z*: [M+H]^+^ calcd for C_34_H_40_N_6_O_5_ 631.3044; found 631.3042.

(*S*)‐*N* ^1^‐[(*S*)‐1‐{[(*R*)‐1‐(4‐Fluorophenyl)‐2‐oxoazetidin‐3‐yl]amino}‐1‐oxo‐4‐phenylbutan‐2‐yl]‐*N* ^4^‐neopentyl‐2‐(pyrazine‐2‐carboxamido)succinamide (**68**): white solid, 80%, ^1^H NMR (500 MHz, DMSO‐*d*_6_) *δ* 9.20 (d, *J* = 1.0 Hz, 1H, pyrazine‐H), 8.94–8.87 (m, 3H, pyrazine‐H + NH), 8.76 (dd, *J* = 2.5, 1.5 Hz, 1H, pyrazine‐H), 8.46 (d, *J* = 7.5 Hz, 1H, NH), 8.03 (t, *J* = 6.0 Hz, 1H, NH), 7.41–7.36 (m, 2H, Ar‐H), 7.27–7.19 (m, 4H, Ar‐H), 7.19–7.14 (m, 3H, Ar‐H), 5.18–5.13 (m, 1H, CH), 4.87–4.80 (m, 1H, CH), 4.21–4.15 (m, 1H, CH), 3.95 (t, *J* = 5.5 Hz, 1H, CH_2_), 3.55 (dd, *J* = 5.0, 2.5 Hz, 1H, CH_2_), 2.94 (dd, *J* = 15.0, 6.0 Hz, 1H, CH_2_), 2.85 (dd, *J* = 14.5, 5.5 Hz, 1H, CH_2_), 2.74–2.66 (m, 2H, CH_2_), 2.65–2.54 (m, 2H, CH_2_), 2.15–2.07 (m, 1H, CH_2_), 1.92–1.82 (m, 1H, CH_2_), 0.60 (s, 9H, CH_3_). ^13^C NMR (125 MHz, DMSO‐*d*_6_) *δ* 172.04, 170.85, 170.32, 164.77, 162.74, 159.65, 157.74, 148.39, 144.51, 143.92, 143.82, 141.62, 135.22, 128.81 (d, *J* = 15.3 Hz), 126.33, 118.26 (d, *J* = 7.9 Hz), 116.42 (d, *J* = 22.7 Hz), 55.25, 53.03, 50.63, 50.03, 47.24, 37.80, 33.27, 32.11, 31.83, 27.38. HPLC purity: 100.0%. HRAM‐MS (ESI) *m*/*z*: [M+H]^+^ calcd for C_33_H_39_FN_7_O_5_ 632.2997; found 632.3014.

(*S*)‐*N* ^1^‐[(*S*)‐1‐{[(*S*)‐1‐(4‐Fluorophenyl)‐2‐oxoazetidin‐3‐yl]amino}‐1‐oxo‐4‐phenylbutan‐2‐yl]‐*N* ^4^‐neopentyl‐2‐(pyrazine‐2‐carboxamido)succinamide (**69**): white solid, 80%, ^1^H NMR (400 MHz, DMSO‐*d*_6_) *δ* 9.23 (s, 1H, pyrazine‐H), 8.98 (d, *J* = 7.6 Hz, 1H, NH), 8.93 (d, *J* = 7.2 Hz, 1H, pyrazine‐H), 8.85 (d, *J* = 8.0 Hz, 1H, NH), 8.79 (d, *J* = 8.0 Hz, 1H, pyrazine‐H), 8.44 (d, *J* = 7.6 Hz, 1H, NH), 8.02 (t, *J* = 5.2 Hz, 1H, NH), 7.40–7.37 (m, 2H, Ar‐H), 7.32–7.21 (m, 4H, Ar‐H), 7.19–7.15 (m, 3H, Ar‐H), 5.15–5.06 (m, 1H, CH), 4.92–4.82 (m, 1H, CH), 4.30–4.19 (m, 1H, CH), 3.96 (t, *J* = 4.8 Hz, 1H, CH_2_), 3.65–3.54 (m, 1H, CH_2_), 2.93 (dd, *J* = 14.4, 5.6 Hz, 1H, CH_2_), 2.87–2.74 (m, 3H, CH_2_), 2.72–2.56 (m, 2H, CH_2_), 2.18–2.05 (m, 1H, CH_2_), 1.96–1.81 (m, 1H, CH_2_), 0.69 (s, 9H, CH_3_). ^13^C NMR (100 MHz, DMSO‐*d*_6_) *δ* 171.50, 170.31, 169.83, 164.20, 162.37, 159.38, 147.85, 144.11, 143.41, 143.35, 141.21, 134.73, 134.71, 128.30 (d, *J* = 10.4 Hz), 125.82, 117.75 (d, *J* = 8.0 Hz), 115.92 (d, *J* = 22.7 Hz), 55.01, 52.28, 50.23, 49.63, 46.31, 37.28, 32.93, 31.69, 31.29, 26.98. HPLC purity: 100.0%. HRAM‐MS (ESI) *m*/*z*: [M+H]^+^ calcd for C_33_H_39_FN_7_O_5_ 632.2997; found 632.3015.

*N*‐[(*S*)‐1‐{[(*S*)‐1‐{[(*R*)‐1‐(4‐Fluorophenyl)‐2‐oxoazetidin‐3‐yl]amino}‐1‐oxo‐4‐phenylbutan‐2‐yl]amino}‐1‐oxopropan‐2‐yl]nicotinamide (**70**): white solid, 55%, ^1^H NMR (400 MHz, DMSO‐*d*_6_) *δ* 9.05 (d, *J* = 1.6 Hz, 1H, pyridine‐H), 8.85 (d, *J* = 6.8 Hz, 1H, NH), 8.71–8.68 (m, 1H, pyridine‐H), 8.64 (d, *J* = 8.0 Hz, 1H, NH), 8.26–8.17 (m, 2H, pyridine‐H + NH), 7.47 (dd, *J* = 8.0, 4.8 Hz, 1H, pyridine‐H), 7.44–7.34 (m, 2H, Ar‐H), 7.29–7.20 (m, 4H, Ar‐H), 7.20–7.14 (m, 3H, Ar‐H), 5.06–5.01 (m, 1H, CH), 4.56–4.46 (m, 1H, CH), 4.27–4.20 (m, 1H, CH), 3.93 (t, *J* = 5.6 Hz, 1H, CH_2_), 3.57 (dd, *J* = 5.6, 2.8 Hz, 1H, CH_2_), 2.70–2.53 (m, 2H, CH_2_), 2.06–1.80 (m, 2H, CH_2_), 1.39 (d, *J* = 7.2 Hz, 3H, CH_3_). ^13^C NMR (100 MHz, DMSO‐*d*_6_) *δ* 172.34, 171.81, 165.13, 164.25, 159.39, 157.00, 151.94, 148.67, 141.23, 135.26, 134.69 (d, *J* = 2.4 Hz), 129.47, 128.31 (d, *J* = 2.6 Hz), 125.85, 123.32, 117.80 (d, *J* = 8.0 Hz), 115.96 (d, *J* = 22.5 Hz)., 55.05, 52.20, 49.36, 46.60, 33.54, 31.30, 17.42. HPLC purity: 100.0%. HRAM‐MS (ESI) *m*/*z*: [M+H]^+^ calcd for C_28_H_29_FN_5_O_4_ 518.2204; found 518.2219.

*N*‐[(*S*)‐1‐{[(*S*)‐1‐{[(*R*)‐1‐(4‐Fluorophenyl)‐2‐oxoazetidin‐3‐yl]amino}‐1‐oxo‐4‐phenylbutan‐2‐yl]amino}‐1‐oxopropan‐2‐yl]pyrazine‐2‐carboxamide (**71**): white solid, 57%, ^1^H NMR (400 MHz, DMSO‐*d*_6_) *δ* 9.18 (s, 1H, pyrazine‐H), 8.89 (d, *J* = 2.4 Hz, 1H, pyrazine‐H), 8.83 (d, *J* = 7.6 Hz, 1H, NH), 8.79–8.68 (m, 2H, pyrazine‐H + NH), 8.37 (d, *J* = 8.0 Hz, 1H, NH), 7.43–7.36 (m, 2H, Ar‐H), 7.30–7.20 (m, 4H, Ar‐H), 7.20–7.11 (m, 3H, Ar‐H), 5.07–4.99 (m, 1H, CH), 4.65–4.56 (m, 1H, CH), 4.33–4.25 (m, 1H, CH), 3.93 (t, *J* = 5.2 Hz, 1H, CH_2_), 3.58 (dd, *J* = 5.2, 2.4 Hz, 1H, CH_2_), 2.68–2.54 (m, 2H, CH_2_), 2.01–1.83 (m, 2H, CH_2_), 1.41 (d, *J* = 6.8 Hz, 3H, CH_3_). ^13^C NMR (100 MHz, DMSO‐*d*_6_) *δ* 172.24, 172.11, 164.70, 162.88, 159.90, 157.50, 148.24, 144.77, 143.93, 143.88, 141.68, 135.18, 128.79, 128.76, 126.35, 118.30 (d, *J* = 8.1 Hz), 116.43 (d, *J* = 22.5 Hz), 55.62, 52.80, 49.10, 47.13, 34.14, 31.80, 18.91. HPLC purity: 99.7%. HRAM‐MS (ESI) *m*/*z*: [M+H]^+^ calcd for C_27_H_28_FN_6_O_4_ 519.2156; found 519.2175.

*N*‐[(*S*)‐1‐{[(*S*)‐1‐{[(*R*)‐1‐(4‐Fluorophenyl)‐2‐oxoazetidin‐3‐yl]amino}‐1‐oxo‐4‐phenylbutan‐2‐yl]amino}‐3‐methyl‐1‐oxobutan‐2‐yl]nicotinamide (**72**): white solid, 63%, ^1^H NMR (400 MHz, DMSO‐*d*_6_) *δ* 9.05 (d, *J* = 1.6 Hz, 1H, pyridine‐H), 8.72 (dd, *J* = 4.4, 1.6 Hz, 1H, pyridine‐H), 8.70 (d, *J* = 8.4 Hz, 1H, NH), 8.66 (d, *J* = 8.4 Hz, 1H, NH), 8.28 (d, *J* = 8.0 Hz, 1H, NH), 8.23 (d, *J* = 8.0 Hz, 1H, pyridine‐H), 7.51 (dd, *J* = 8.0, 4.8 Hz, 1H, pyridine‐H), 7.44–7.36 (m, 2H, Ar‐H), 7.30–7.21 (m, 4H, Ar‐H), 7.21–7.14 (m, 3H, Ar‐H), 5.11–5.05 (m, 1H, CH), 4.37 (t, *J* = 8.0 Hz, 1H, CH), 4.31–4.23 (m, 1H, CH), 3.95 (t, *J* = 5.6 Hz, 1H, CH_2_), 3.54 (dd, *J* = 5.6, 2.8 Hz, 1H, CH_2_), 2.70–2.57 (m, 2H, CH_2_), 2.24–2.14 (m, 1H, CH), 2.03–1.87 (m, 2H, CH_2_), 0.99 (d, *J* = 6.0 Hz, 6H, CH_3_). ^13^C NMR (100 MHz, DMSO‐*d*_6_) *δ* 171.57, 170.91, 165.45, 164.22, 159.39, 157.00, 151.88, 148.62, 141.16, 135.32, 134.64 (d, *J* = 2.4 Hz), 129.80, 128.29, 125.87, 123.34, 117.79 (d, *J* = 7.9 Hz), 115.95 (d, *J* = 22.6 Hz), 59.27, 55.04, 52.21, 46.51, 33.64, 31.25, 29.80, 19.36, 18.89. HPLC purity: 99.5%. HRAM‐MS (ESI) *m*/*z*: [M+H]^+^ calcd for C_30_H_33_FN_5_O_4_ 546.2517; found 546.2533.

*N*‐[(*S*)‐1‐{[(*S*)‐1‐{[(*R*)‐1‐(4‐Fluorophenyl)‐2‐oxoazetidin‐3‐yl]amino}‐1‐oxo‐4‐phenylbutan‐2‐yl]amino}‐1‐oxo‐4‐phenylbutan‐2‐yl]nicotinamide (**73**): white solid, 60%, ^1^H NMR (500 MHz, DMSO‐*d*_6_) *δ* 9.08 (d, *J* = 1.6 Hz, 1H, pyridine‐H), 8.88 (d, *J* = 7.4 Hz, 1H, NH), 8.73 (dd, *J* = 4.7, 1.5 Hz, 1H, NH), 8.69 (d, *J* = 8.1 Hz, 1H, pyridine‐H), 8.28 (d, *J* = 7.8 Hz, 1H, NH), 8.26–8.22 (m, 1H, pyridine‐H), 7.51 (dd, *J* = 7.8, 4.9 Hz, 1H, pyridine‐H), 7.42–7.36 (m, 2H, Ar‐H), 7.29–7.21 (m, 8H, Ar‐H), 7.20–7.13 (m, 4H, Ar‐H), 5.06–4.99 (m, 1H, CH), 4.57–4.49 (m, 1H, CH), 4.32–4.22 (m, 1H, CH), 3.92 (t, *J* = 5.6 Hz, 1H, CH_2_), 3.56 (dd, *J* = 5.4, 2.7 Hz, 1H, CH_2_), 2.79–2.63 (m, 3H, CH_2_), 2.61–2.54 (m, 1H, CH_2_), 2.17–1.94 (m, 3H, CH_2_), 1.94–1.82 (m, 1H, CH_2_). ^13^C NMR (125 MHz, DMSO‐*d*_6_) *δ* 172.30, 172.08, 165.93, 164.70, 159.63, 157.72, 152.47, 149.20, 141.96, 141.68, 135.79, 135.18, 130.09, 128.79, 126.35, 126.30, 123.84, 118.29, 118.23, 116.53, 116.35, 55.60, 54.03, 52.75, 47.08, 33.96, 33.67, 32.31, 31.80. HPLC purity: 100.0%. HRAM‐MS (ESI) *m*/*z*: [M+H]^+^ calcd for C_35_H_35_FN_5_O_4_ 608.2673; found 608.2667.

*N*‐[(*S*)‐1‐{[(*S*)‐1‐{[(*R*)‐1‐(4‐Fluorophenyl)‐2‐oxoazetidin‐3‐yl]amino}‐1‐oxo‐4‐phenylbutan‐2‐yl]amino}‐1‐oxo‐3‐phenylpropan‐2‐yl]nicotinamide (**74**): white solid, 65%, ^1^H NMR (500 MHz, DMSO‐*d*_6_) *δ* 8.93 (d, *J* = 1.5 Hz, 1H, pyridine‐H), 8.91 (d, *J* = 8.3 Hz, 1H, NH), 8.72 (d, *J* = 8.1 Hz, 1H, NH), 8.68 (dd, *J* = 4.8, 1.6 Hz, 1H, pyridine‐H), 8.43 (d, *J* = 7.8 Hz, 1H, NH), 8.14–8.09 (m, 1H, pyridine‐H), 7.49–7.45 (m, 1H, pyridine‐H), 7.42 – 7.38 (m, 4H, Ar‐H), 7.29–7.24 (m, 6H, Ar‐H), 7.20–7.16 (m, 4H, Ar‐H), 5.08–5.01 (m, 1H, CH), 4.84–4.78 (m, 1H, CH), 4.32–4.25 (m, 1H, CH), 3.94 (t, *J* = 5.6 Hz, 1H, CH_2_), 3.58 (dd, *J* = 5.5, 2.8 Hz, 1H, CH_2_), 3.21 (dd, *J* = 13.8, 3.9 Hz, 1H, CH_2_), 3.01 (dd, *J* = 13.8, 11.0 Hz, 1H, CH_2_), 2.71–2.55 (m, 2H, CH_2_), 2.06–1.85 (m, 2H, CH_2_). ^13^C NMR (125 MHz, DMSO‐*d*_6_) *δ* 172.24, 171.81, 165.57, 164.75, 159.63, 157.72, 152.44, 148.99, 141.67, 138.77, 135.59, 135.20, 130.01, 129.67, 128.80, 128.56, 126.75, 126.36, 123.85, 118.30, 118.24, 116.55, 116.37, 55.60, 55.36, 52.87, 47.05, 37.28, 34.15, 31.74. HPLC purity: 98.2%. HRAM‐MS (ESI) *m*/*z*: [M+H]^+^ calcd for C_34_H_33_FN_5_O_4_ 594.2517; found 594.2517.

*N*‐[(*S*)‐1‐{[(*S*)‐1‐{[(*R*)‐1‐(4‐Fluorophenyl)‐2‐oxoazetidin‐3‐yl]amino}‐1‐oxo‐4‐phenylbutan‐2‐yl]amino}‐4‐(methylthio)‐1‐oxobutan‐2‐yl]nicotinamide (**75**): white solid, 74%, ^1^H NMR (500 MHz, DMSO‐*d*_6_) *δ* 9.04 (dd, *J* = 2.0, 0.5 Hz, 1H, pyridine‐H), 8.89 (d, *J* = 7.0 Hz, 1H, NH), 8.69 (dd, *J* = 5.0, 1.5 Hz, 1H, pyridine‐H), 8.65 (d, *J* = 8.0 Hz, 1H, NH), 8.58 (d, *J* = 8.0 Hz, 1H, NH), 8.18–8.12 (m, 1H, pyridine‐H), 7.40–7.36 (m, 3H, pyridine‐H + Ar‐H), 7.30–7.21 (m, 4H, Ar‐H), 7.20–7.15 (m, 3H, Ar‐H), 5.12–5.07 (m, 1H, CH), 4.54 (q, *J* = 7.0 Hz, 1H, CH), 4.28–4.20 (m, 1H, CH), 3.94 (t, *J* = 6.0 Hz, 1H, CH_2_), 3.50 (dd, *J* = 5.5, 2.5 Hz, 1H, CH_2_), 2.69–2.58 (m, 2H, CH_2_), 2.58–2.51 (m, 2H, CH_2_), 2.07 (s, 3H, CH_3_), 2.06–2.00 (m, 3H, CH_3_), 1.91–1.82 (m, 1H, CH_2_). ^13^C NMR (125 MHz, DMSO‐*d*_6_) *δ* 172.18, 172.00, 166.03, 164.61, 159.64, 157.73, 152.57, 149.15, 141.55, 135.74, 135.16, 129.64, 128.83, 128.77, 126.41, 123.74, 118.27 (d, *J* = 8.0 Hz), 116.47 (d, *J* = 22.6 Hz), 55.52, 54.03, 52.61, 47.30, 33.74, 31.90, 31.47, 30.34, 15.11. HPLC purity: 96.7%. HRAM‐MS (ESI) *m*/*z*: [M+H]^+^ calcd for C_30_H_33_FN_5_O_4_S 578.2237; found 578.2245.

(*S*)‐*N* ^4^‐Neopentyl‐2‐(nicotinamido)‐*N* ^1^‐[(*S*)‐1‐oxo‐1‐{[(*R*)‐2‐oxo‐1‐(p‐tolyl)azetidin‐3‐yl]amino}‐4‐phenylbutan‐2‐yl]succinamide (**76**): white solid, 56%, ^1^H NMR (500 MHz, DMSO‐*d*_6_) *δ* 9.02 (d, *J* = 1.5 Hz, 1H, pyridine‐H), 8.91 (d, *J* = 8.0 Hz, 1H, NH), 8.78 (d, *J* = 8.5 Hz, 1H, NH), 8.71 (dd, *J* = 5.0, 1.5 Hz, 1H, pyridine‐H), 8.46 (d, *J* = 8.0 Hz, 1H, NH), 8.22–8.16 (m, 1H, pyridine‐H), 7.91 (t, *J* = 6.0 Hz, 1H, NH), 7.50 (dd, *J* = 7.5, 4.5 Hz, 1H, pyridine‐H), 7.26–7.20 (m, 4H, Ar‐H), 7.19–7.10 (m, 5H, Ar‐H), 5.13–5.07 (m, 1H, CH), 4.82 (q, *J* = 7.0 Hz, 1H, CH), 4.19–4.13 (m, 1H, CH), 3.91 (t, *J* = 5.5 Hz, 1H, CH_2_), 3.52 (dd, *J* = 5.5, 3.0 Hz, 1H, CH_2_), 2.87–2.80 (m, 2H, CH_2_), 2.76–2.69 (m, 2H, CH_2_), 2.69–2.63 (m, 1H, CH_2_), 2.60–2.53 (m, 1H, CH_2_), 2.26 (s, 3H, CH_3_), 2.12–2.03 (m, 1H, CH_2_), 1.92–1.83 (m, 1H, CH_2_), 0.71 (s, 9H, CH_3_). ^13^C NMR (125 MHz, DMSO‐*d*_6_) *δ* 172.19, 171.56, 170.26, 165.38, 164.63, 152.52, 149.08, 141.65, 136.27, 135.67, 133.17, 130.02, 129.94, 128.90, 128.72, 126.30, 123.87, 116.58, 55.08, 52.69, 51.36, 50.13, 46.81, 37.64, 33.47, 32.20, 31.71, 27.52, 20.95. HPLC purity: 99.4%. HRAM‐MS (ESI) *m*/*z*: [M+H]^+^ calcd for C_35_H_43_N_6_O_5_ 627.3295; found 627.3303.

(*S*)‐*N* ^1^‐[(*S*)‐1‐{[(*R*)‐1‐[4‐(Dimethylamino)phenyl]‐2‐oxoazetidin‐3‐yl]amino}‐1‐oxo‐4‐phenylbutan‐2‐yl]‐*N* ^4^‐neopentyl‐2‐(nicotinamido)succinamide (**77**): white solid, 53%, ^1^H NMR (500 MHz, DMSO‐*d*_6_) *δ* 9.03 (d, *J* = 1.5 Hz, 1H, pyridine‐H), 8.93 (d, *J* = 8.0 Hz, 1H, NH), 8.77 (d, *J* = 8.5 Hz, 1H, NH), 8.73 (dd, *J* = 4.5, 1.0 Hz, 1H, pyridine‐H), 8.45 (d, *J* = 7.5 Hz, 1H, NH), 8.26–8.20 (m, 1H, pyridine‐H), 7.91 (t, *J* = 6.0 Hz, 1H, NH), 7.54 (dd, *J* = 8.0, 5.0 Hz, 1H, pyridine‐H), 7.28–7.20 (m, 5H, Ar‐H), 7.19–7.12 (m, 4H, Ar‐H), 5.10–5.06 (m, 1H, CH), 4.82 (dd, *J* = 14.0, 7.0 Hz, 1H, CH), 4.18–4.13 (m, 1H, CH), 3.88 (t, *J* = 5.5 Hz, 1H, CH_2_), 3.50 (dd, *J* = 5.0, 2.5 Hz, 1H, CH_2_), 2.90 (s, 6H, CH_3_), 2.86–2.80 (m, 2H, CH_2_), 2.75–2.66 (m, 3H, CH_2_), 2.58–2.52 (m, 1H, CH_2_), 2.09–2.02 (m, 1H, CH_2_), 1.90–1.82 (m, 1H, CH_2_), 0.71 (s, 9H, CH_3_). ^13^C NMR (125 MHz, DMSO‐*d*_6_) *δ* 172.15, 171.53, 170.23, 165.22, 152.11, 148.71, 141.66, 136.17, 130.10, 128.90, 128.72, 126.31, 124.07, 117.73, 55.05, 52.70, 51.37, 50.12, 46.88, 37.62, 33.50, 32.21, 31.72, 27.54. HPLC purity: 95.8%. HRAM‐MS (ESI) *m*/*z*: [M+H]^+^ calcd for C_36_H_46_N_7_O_5_ 656.3560; found 656.3565.

(*S*)‐*N* ^1^‐[(*S*)‐1‐{[(*R*)‐1‐(4‐Methoxyphenyl)‐2‐oxoazetidin‐3‐yl]amino}‐1‐oxo‐4‐phenylbutan‐2‐yl]‐*N* ^4^‐neopentyl‐2‐(nicotinamido)succinimide (**78**): white solid, 56%, ^1^H NMR (500 MHz, DMSO‐*d*_6_) *δ* 9.03 (d, *J* = 2.0 Hz, 1H, pyridine‐H), 8.92 (d, *J* = 7.5 Hz, 1H, NH), 8.78 (d, *J* = 8.5 Hz, 1H, NH), 8.71 (dd, *J* = 5.0, 1.5 Hz, 1H, pyridine‐H), 8.47 (d, *J* = 8.0 Hz, 1H, NH), 8.25–8.16 (m, 1H, pyridine‐H), 7.92 (t, *J* = 6.0 Hz, 1H, NH), 7.51 (dd, *J* = 7.5, 5.0 Hz, 1H, pyridine‐H), 7.34–7.28 (m, 2H, Ar‐H), 7.27–7.20 (m, 2H, Ar‐H), 7.19–7.13 (m, 3H, Ar‐H), 6.98–6.91 (m, 2H, Ar‐H), 5.14–5.08 (m, 1H, CH), 4.83 (q, *J* = 7.0 Hz, 1H, CH), 4.19–4.13 (m, 1H, CH), 3.91 (t, *J* = 5.5 Hz, 1H, CH_2_), 3.72 (s, 3H, CH_3_), 3.51 (dd, *J* = 5.5, 2.5 Hz, 1H, CH_2_), 2.89–2.80 (m, 2H, CH_2_), 2.76–2.64 (m, 3H, CH_2_), 2.60–2.52 (m, 1H, CH_2_), 2.14–2.03 (m, 1H, CH_2_), 1.93–1.82 (m, 1H, CH_2_), 0.71 (s, 9H, CH_3_). ^13^C NMR (125 MHz, DMSO‐*d*_6_) *δ* 172.17, 171.56, 170.25, 165.37, 164.19, 156.02, 152.50, 149.06, 141.65, 135.70, 132.22, 129.94, 128.90, 128.71, 126.30, 123.89, 117.89, 114.90, 55.76, 55.10, 52.69, 51.35, 50.12, 46.96, 37.63, 33.46, 32.21, 31.71, 27.52. HPLC purity: 96.6%. HRAM‐MS (ESI) *m*/*z*: [M+H]^+^ calcd for C_35_H_43_N_6_O_6_ 643.3244; found 643.3245.

(*S*)‐*N* ^4^‐Neopentyl‐2‐(nicotinamido)‐*N* ^1^‐[(*S*)‐1‐oxo‐1‐{[(*R*)‐2‐oxo‐1‐[4‐(trifluoromethyl)phenyl]azetidin‐3‐yl]amino}‐4‐phenylbutan‐2‐yl]succinamide (**79**): white solid, 64%, ^1^H NMR (500 MHz, DMSO‐*d*_6_) *δ* 9.03 (d, *J* = 2.0 Hz, 1H, pyridine‐H), 8.92 (d, *J* = 7.5 Hz, 1H, NH), 8.85 (d, *J* = 8.5 Hz, 1H, NH), 8.72 (dd, *J* = 5.0, 1.5 Hz, 1H, pyridine‐H), 8.43 (d, *J* = 8.0 Hz, 1H, NH), 8.22–8.20 (m, 1H, pyridine‐H), 7.95 (t, *J* = 6.0 Hz, 1H, NH), 7.52–7.50 (m, 1H, pyridine‐H), 7.27–7.20 (m, 4H, Ar‐H), 7.18–7.11 (m, 5H, Ar‐H), 5.22–5.16 (m, 1H, CH), 4.88–4.83 (m, 1H, CH), 4.18–4.12 (m, 1H, CH), 4.03–3.99 (m, 1H, CH_2_), 3.62–3.60 (m, 1H, CH_2_), 2.92 (dd, *J* = 13.5, 6.5 Hz, 1H, CH_2_), 2.88–2.84 (m, 1H, CH_2_), 2.71–2.68 (m, 2H, CH_2_), 2.61–2.53 (m, 2H, CH_2_), 2.13–2.06 (m, 1H, CH_2_), 1.99–1.94 (m, 1H, CH_2_), 0.68 (s, 9H, CH_3_). ^13^C NMR (125 MHz, DMSO‐*d*_6_) *δ* 172.12, 171.58, 170.34, 165.35, 152.50, 149.06, 141.61, 135.70, 129.95, 128.91, 128.72, 126.31, 123.92, 116.92, 55.46, 52.54, 51.68, 50.10, 47.11, 37.66, 33.31, 32.12, 31.69, 27.6. HPLC purity: 100.0%. HRAM‐MS (ESI) *m*/*z*: [M+H]^+^ calcd for C_35_H_40_F_3_N_6_O_5_ 681.3012; found 681.3029.

### 4.2 Pharmacological/Biological Assays

#### 4.2.1 In Vitro 20S Proteasome Chymotrypsin‐Like Inhibition Assay

The human constitutive proteasome was generously provided by Dr. Jiang‐ping Wu (Notre‐Dame Hospital, Montreal, QC, Canada). The inhibition of enzyme activity was assessed by monitoring the decrease in hydrolysis of the fluorogenic substrate Suc‐LLVY‐AMC for ChT‐L. All compounds were initially dissolved in DMSO and subsequently diluted with water to achieve a 10% DMSO solution. The proteasome activity assay was performed in a total volume of 50 μL, with all samples diluted in Tris–HCl buffer (100 mM Tris–HCl, pH 8.0). Reactions were conducted in 384‐well polystyrene microplates. 10 μL purified human proteasome (25 μg/mL) was added to 1 μL of each compound; after incubation for 15 min, the mixture was combined with 39 μL of the fluorogenic substrate. The release of the AMC moiety was detected by monitoring the increase of fluorescence with the Envision system, using 355 nm excitation and 460 nm emission wavelengths. The IC_50_ values were calculated using the software GraphPad Prism4, employing the “sigmoidal dose–response (variable slope)” equation for curve fitting.

#### 4.2.2 Cell Proliferation Inhibition Assay

MV‐4‐11 cells (1 × 10^4^ cells/well), RPMI‐8226 cells (8 × 10^3^ cells/well), Molt4 cells (2 × 10^3^ cells/well), RS4;11 cells (2 × 10^4^ cells/well), and PBMC cells (2 × 10^4^ cells/well) were seeded in 96‐well plates (Corning, 3599). Cells were treated with either 0.2% DMSO or fivefold dilutions of compounds from 10 mM stock solutions in DMSO (0.2% final concentration of DMSO) for 72 h. 10 μL of CCK8 reagent was added to each well, and the plates were incubated at 37°C for 3 h. Absorbance was measured at 450 nm and 650 nm using a Spectra Max Molecular Devices microplate reader. The final data were calibrated by OD_450 nm_‐OD_650 nm_. Proliferation inhibition rates were calculated using the following equation: Inhibition ratio = (OD_DMSO_‐OD_Compd_)/(OD_DMSO_‐OD_Blank_) × 100%. The concentrations of the compounds that inhibited cell growth by 50% (IC_50_) were calculated using GraphPad Prism version 9.5.

#### 4.2.3 Apoptosis Assay and Western Blot Analysis

Apoptosis was assessed using an Annexin V‐APC/7AAD apoptosis detection kit (GA1023‐KGA1026, Keygentec, Nanjing, China), followed by flow cytometry analysis. Briefly, cells were treated with the indicated drug concentration and durations or left untreated, then washed twice with PBS, stained with Annexin V‐APC/7AAD, and analyzed using flow cytometry (CytoFLEX flow cytometer, Beckman Coulter Inc.). RS4;11 cells were exposed to various concentrations of the compound or DMSO. Protein lysates were prepared using 4 × Laemmli sample buffer (161073, BIORAD, Richmond, USA). Protein samples were separated by SDS‐polyacrylamide gel electrophoresis (BIO‐RAD, Richmond, USA) and subsequently transferred to nitrocellulose (NC) membranes. Following a 90‐min blocking step with 5% skim milk at room temperature, the NC membranes were incubated with the primary antibody at 4 ^o^C overnight, followed by three washes with TBST for 10 min each. The NC membranes were then incubated with secondary antibodies for 1.5 h at room temperature. Signals were detected by ECL (KF8001&KF8003, Affinity, China) using the BIO‐RAD ChemiDoc Touch Imaging System.

### 4.3 Computational Methods

#### 4.3.1 Molecular Docking Studies

The molecular docking procedure was conducted using *Glide* (*Schrödinger version 2021‐2*) with the default option (standard precision mode). To analyze the binding interaction of target compounds with the active site of proteasome, the protein structure of 20S proteasome was utilized, obtained from the RCSB Protein Data Bank (PDB) as entry 4R3O. The protein was prepared using the Protein Preparation *Wizard* in *Schrödinger's Maestro* environment by removing nonrelevant ligands and solvent molecules, retaining crystallographic water molecules involved in the binding site. Missing residues were repaired, bond orders fixed, hydrogen atoms added, and the protein structure optimized with OPLS_2005 force field. For the preparation of ligands, the 3D structures were generated, and their energies were minimized in *LigPrep*. A grid box measuring 15 Å × 15 Å × 15 Å was created, centered at the native ligand of the selected protein with coordinates (x,y,z): 177.34, −25.55, and 1.82. Interactions of the docked proteasome with the ligand were analyzed, and the docking conformations were selected and saved based on the calculated *Glide* docking energy score. Graphical images were created using *Pymol*.

#### 4.3.2 MD Studies

To investigate the interactions within the molecule **76**‐proteasome complex, MD simulations were conducted using *Gromacs 2020* software. The topology of the proteasome protein was generated using the *AMBER99SB‐ILDN* force field, while the topology of the ligand was generated by *sobtop* server using *gaff* force field. The *TIP3P* explicit water model was selected, with a minimum distance of 1.0 nm between the protein atoms and the edges of the water box. Sodium or chloride ions were added to neutralize the system charge based on the docking results. Further, energy minimization was done, and the minimized systems were subjected to NPT (Number of particles Pressure Temperature) equilibration of 50 ps. Finally, the systems were subjected to the production MD runs of 100 ns.

## Conflicts of Interest

The authors declare no conflicts of interest.

## Supporting information

ArchPharm SupplMat InChI.

Supporting_information.

## Data Availability

The data that support the findings of this study are available in the supplementary material of this article.
