# Supplementary material for: Identification of Novel β‐Lactam Derivatives as Proteasome Inhibitors for Antitumor Therapy
Source: Arch Pharm (Weinheim). 2025 Nov 21;358(11):e70136. doi: 10.1002/ardp.70136 (PMC12638514; doi:10.1002/ardp.70136)
Supplement: Supplementary file 1 — ArchPharm SupplMat InChI. [file ARDP-358-e70136-s002.doc]

**Supplemental Material: Novel Compounds and Biological Screening Results**

Identification of novel *β*-lactam derivatives as proteasome inhibitors for antitumor therapy

Yu Cao^1, #^, Gaoya Xu^2, #^, Lixin Gao^2^, Jingjing Sun^1^, Lu Zhang^1^, Qiao Tong^1^, Limin Kong^3^, Jiankang Zhang^4^, Yubo Zhou^2, 7^, Li Liao^5, *^, , Liping Fu^6, *^, , Jianjun Xi^1, *^

1 Department of Pharmaceutical Preparation, Hangzhou Xixi Hospital, Hangzhou, 310023, China

2 State Key Laboratory of Chemical Biology, Shanghai Institute of Materia Medica, Chinese Academy of Sciences, Shanghai 201203, China

3 Department of Clinical Pharmacy, the First Affiliated Hospital, Zhejiang University, School of Medicine, Hangzhou, 310003, China

4 Key Laboratory of Novel Targets and Drug Study for Neural Repair of Zhejiang Province, School of Medicine, Hangzhou City University, Hangzhou, 310015, China

5 Department of Pharmacy, Hangzhou Children’s Hospital, Hangzhou, 310014, China

6 Department of Pharmacy, Shaoxing TCM Hospital Affiliated to Zhejiang Chinese Medical University, Shaoxing, 312000, China

7 Zhongshan Institute for Drug Discovery, Shanghai Institute of Materia Medica, Chinese Academy of Sciences, Zhongshan, 528400, China

*Correspondence:

Li Liao, Department of Pharmacy, Hangzhou Children’s Hospital, Hangzhou, 310014, China.

Email: liaoli0101@sina.com

Liping Fu, Department of Pharmacy, Shaoxing TCM Hospital Affiliated to Zhejiang Chinese Medical University, Shaoxing, 312000, China.

Email: fuliping100@163.com

Jianjun Xi, Department of Pharmaceutical Preparation, Hangzhou Xixi Hospital, Hangzhou, 310023, China.

Email: xjianjun@foxmail.com

| **Compound No.** | **InChI** | **Biological Activity ( IC_50_)^a^** |
| --- | --- | --- |
| 64 | InChI=1S/C34H40N6O5/c1-34(2,3)22-36-29(41)19-27(38-30(42)24-13-10-18-35-20-24)32(44)37-26(17-16-23-11-6-4-7-12-23)31(43)39-28-21-40(33(28)45)25-14-8-5-9-15-25/h4-15,18,20,26-28H,16-17,19,21-22H2,1-3H3,(H,36,41)(H,37,44)(H,38,42)(H,39,43)/t26-,27-,28+/m0/s1 | 186.85±35.28 nM |
| 65 | InChI=1S/C34H40N6O5/c1-34(2,3)22-36-29(41)19-27(38-30(42)24-13-10-18-35-20-24)32(44)37-26(17-16-23-11-6-4-7-12-23)31(43)39-28-21-40(33(28)45)25-14-8-5-9-15-25/h4-15,18,20,26-28H,16-17,19,21-22H2,1-3H3,(H,36,41)(H,37,44)(H,38,42)(H,39,43)/t26-,27-,28-/m0/s1 | >10000 nM |
| 66 | InChI=1S/C34H39FN6O5/c1-34(2,3)21-37-29(42)18-27(39-30(43)23-10-7-17-36-19-23)32(45)38-26(16-11-22-8-5-4-6-9-22)31(44)40-28-20-41(33(28)46)25-14-12-24(35)13-15-25/h4-10,12-15,17,19,26-28H,11,16,18,20-21H2,1-3H3,(H,37,42)(H,38,45)(H,39,43)(H,40,44)/t26-,27-,28+/m0/s1 | 114.70±1.70 nM |
| 67 | InChI=1S/C34H39FN6O5/c1-34(2,3)21-37-29(42)18-27(39-30(43)23-10-7-17-36-19-23)32(45)38-26(16-11-22-8-5-4-6-9-22)31(44)40-28-20-41(33(28)46)25-14-12-24(35)13-15-25/h4-10,12-15,17,19,26-28H,11,16,18,20-21H2,1-3H3,(H,37,42)(H,38,45)(H,39,43)(H,40,44)/t26-,27-,28-/m0/s1 | 1474.00±280.01 nM |
| 68 | InChI=1S/C33H38FN7O5/c1-33(2,3)20-37-28(42)17-25(39-31(45)26-18-35-15-16-36-26)30(44)38-24(14-9-21-7-5-4-6-8-21)29(43)40-27-19-41(32(27)46)23-12-10-22(34)11-13-23/h4-8,10-13,15-16,18,24-25,27H,9,14,17,19-20H2,1-3H3,(H,37,42)(H,38,44)(H,39,45)(H,40,43)/t24-,25-,27-/m0/s1 | >10000 nM |
| 69 | InChI=1S/C33H38FN7O5/c1-33(2,3)20-37-28(42)17-25(39-31(45)26-18-35-15-16-36-26)30(44)38-24(14-9-21-7-5-4-6-8-21)29(43)40-27-19-41(32(27)46)23-12-10-22(34)11-13-23/h4-8,10-13,15-16,18,24-25,27H,9,14,17,19-20H2,1-3H3,(H,37,42)(H,38,44)(H,39,45)(H,40,43)/t24-,25-,27+/m0/s1 | 288.45±57.20 nM |
| 70 | InChI=1S/C28H28FN5O4/c1-18(31-26(36)20-8-5-15-30-16-20)25(35)32-23(14-9-19-6-3-2-4-7-19)27(37)33-24-17-34(28(24)38)22-12-10-21(29)11-13-22/h2-8,10-13,15-16,18,23-24H,9,14,17H2,1H3,(H,31,36)(H,32,35)(H,33,37)/t18-,23-,24+/m0/s1 | >10000 nM |
| 71 | InChI=1S/C27H27FN6O4/c1-17(31-26(37)22-15-29-13-14-30-22)24(35)32-21(12-7-18-5-3-2-4-6-18)25(36)33-23-16-34(27(23)38)20-10-8-19(28)9-11-20/h2-6,8-11,13-15,17,21,23H,7,12,16H2,1H3,(H,31,37)(H,32,35)(H,33,36)/t17-,21-,23+/m0/s1 | >10000 nM |
| 72 | InChI=1S/C30H32FN5O4/c1-19(2)26(35-27(37)21-9-6-16-32-17-21)29(39)33-24(15-10-20-7-4-3-5-8-20)28(38)34-25-18-36(30(25)40)23-13-11-22(31)12-14-23/h3-9,11-14,16-17,19,24-26H,10,15,18H2,1-2H3,(H,33,39)(H,34,38)(H,35,37)/t24-,25+,26-/m0/s1 | >10000 nM |
| 73 | InChI=1S/C35H34FN5O4/c36-27-15-17-28(18-16-27)41-23-31(35(41)45)40-34(44)30(20-14-25-10-5-2-6-11-25)39-33(43)29(19-13-24-8-3-1-4-9-24)38-32(42)26-12-7-21-37-22-26/h1-12,15-18,21-22,29-31H,13-14,19-20,23H2,(H,38,42)(H,39,43)(H,40,44)/t29-,30-,31+/m0/s1 | >10000 nM |
| 74 | InChI=1S/C34H32FN5O4/c35-26-14-16-27(17-15-26)40-22-30(34(40)44)39-32(42)28(18-13-23-8-3-1-4-9-23)37-33(43)29(20-24-10-5-2-6-11-24)38-31(41)25-12-7-19-36-21-25/h1-12,14-17,19,21,28-30H,13,18,20,22H2,(H,37,43)(H,38,41)(H,39,42)/t28-,29-,30+/m0/s1 | >10000 nM |
| 75 | InChI=1S/C30H32FN5O4S/c1-41-17-15-25(33-27(37)21-8-5-16-32-18-21)29(39)34-24(14-9-20-6-3-2-4-7-20)28(38)35-26-19-36(30(26)40)23-12-10-22(31)11-13-23/h2-8,10-13,16,18,24-26H,9,14-15,17,19H2,1H3,(H,33,37)(H,34,39)(H,35,38)/t24-,25-,26+/m0/s1 | >10000 nM |
| 76 | InChI=1S/C35H42N6O5/c1-23-12-15-26(16-13-23)41-21-29(34(41)46)40-32(44)27(17-14-24-9-6-5-7-10-24)38-33(45)28(19-30(42)37-22-35(2,3)4)39-31(43)25-11-8-18-36-20-25/h5-13,15-16,18,20,27-29H,14,17,19,21-22H2,1-4H3,(H,37,42)(H,38,45)(H,39,43)(H,40,44)/t27-,28-,29+/m0/s1 | 72.03±14.89 nM |
| 77 | InChI=1S/C36H45N7O5/c1-36(2,3)23-38-31(44)20-29(40-32(45)25-12-9-19-37-21-25)34(47)39-28(18-13-24-10-7-6-8-11-24)33(46)41-30-22-43(35(30)48)27-16-14-26(15-17-27)42(4)5/h6-12,14-17,19,21,28-30H,13,18,20,22-23H2,1-5H3,(H,38,44)(H,39,47)(H,40,45)(H,41,46)/t28-,29-,30+/m0/s1 | 132.25±14.78 nM |
| 78 | InChI=1S/C35H42N6O6/c1-35(2,3)22-37-30(42)19-28(39-31(43)24-11-8-18-36-20-24)33(45)38-27(17-12-23-9-6-5-7-10-23)32(44)40-29-21-41(34(29)46)25-13-15-26(47-4)16-14-25/h5-11,13-16,18,20,27-29H,12,17,19,21-22H2,1-4H3,(H,37,42)(H,38,45)(H,39,43)(H,40,44)/t27-,28-,29+/m0/s1 | 141.60±30.12 nM |
| 79 | InChI=1S/C35H39F3N6O5/c1-34(2,3)21-40-29(45)18-27(42-30(46)23-10-7-17-39-19-23)32(48)41-26(16-11-22-8-5-4-6-9-22)31(47)43-28-20-44(33(28)49)25-14-12-24(13-15-25)35(36,37)38/h4-10,12-15,17,19,26-28H,11,16,18,20-21H2,1-3H3,(H,40,45)(H,41,48)(H,42,46)(H,43,47)/t26-,27-,28+/m0/s1 | 282.35±33.60 nM |

^a^ Compounds 64~79 were screened for their in vitro proteasome CT-L inhibitory activities, IC_50_ data was expressed as the mean ± SD from three independent experiments.
