# Supplementary material for: Identification of Novel β‐Lactam Derivatives as Proteasome Inhibitors for Antitumor Therapy
Source: Arch Pharm (Weinheim). 2025 Nov 21;358(11):e70136. doi: 10.1002/ardp.70136 (PMC12638514; doi:10.1002/ardp.70136)
Supplement: Supplementary file 2 — Supporting_information. [file ARDP-358-e70136-s001.docx]

**Supporting Information**

**Identification of novel *β*-lactam derivatives as proteasome inhibitors for antitumor therapy**

Yu Cao^1,^ **^#^**, Gaoya Xu^2, #^, Lixin Gao^2^, Jingjing Sun^1^, Lu Zhang^1^, Qiao Tong^1^, Limin Kong^3^, Jiankang Zhang^4^, Yubo Zhou^2, 7^, Li Liao^5, *^, Liping Fu^6, *^, Jianjun Xi^1, *^

*^1^Department of Pharmaceutical Preparation, Hangzhou Xixi Hospital, Hangzhou, 310023, China*

*^2^State Key Laboratory of Chemical Biology, Shanghai Institute of Materia Medica, Chinese Academy of Sciences, Shanghai 201203, China*

*^3^Department of Clinical Pharmacy, the First Affiliated Hospital, Zhejiang University, School of Medicine, Hangzhou, 310003, China*

*^4^Key Laboratory of Novel Targets and Drug Study for Neural Repair of Zhejiang Province, School of Medicine, Hangzhou City University, Hangzhou, 310015, China*

*^5^Department of Pharmacy, Hangzhou Children’s Hospital, Hangzhou, 310014, China*

*^6^Department of Pharmacy, Shaoxing TCM Hospital Affiliated to Zhejiang Chinese Medical University, Shaoxing, 312000, China*

*^7^Zhongshan Institute for Drug Discovery, Shanghai Institute of Materia Medica, Chinese Academy of Sciences, Zhongshan 528400, China*

**^#^** Contributed equally

* Corresponding author

E-mail addresses: liaoli0101@sina.com (L. Liao); fuliping100@163.com (J. Zhang); [xjianjun@foxmail.com](mailto:xjianjun@foxmail.com) (J. Xi)

***Table of contents***

| **Title page** | **S1** |
| --- | --- |
| **Table of contents** | **S2** |
| **Copies of NMR spectra, HRMS spectra and purity determination for target compounds and the key intermediates** | **S3** |

**Copies of NMR spectra, HRMS spectra and purity determination**


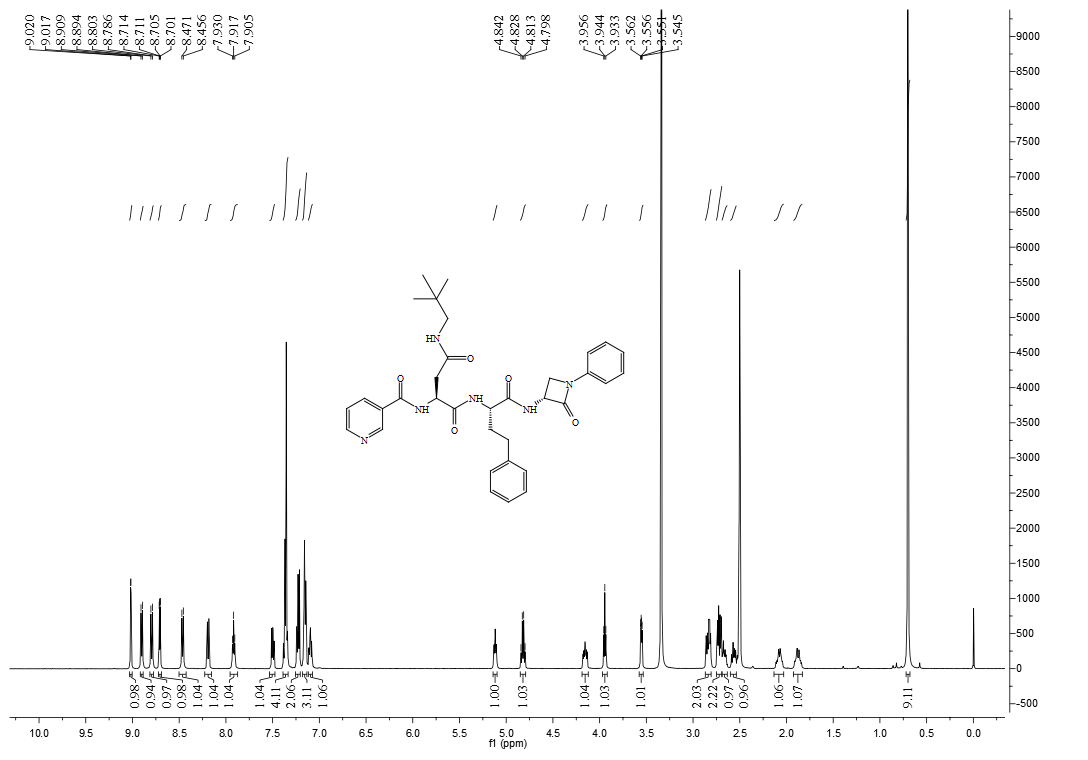


**Figure S1**. ^1^H NMR spectrum of compound **64** in DMSO-*d*_6_


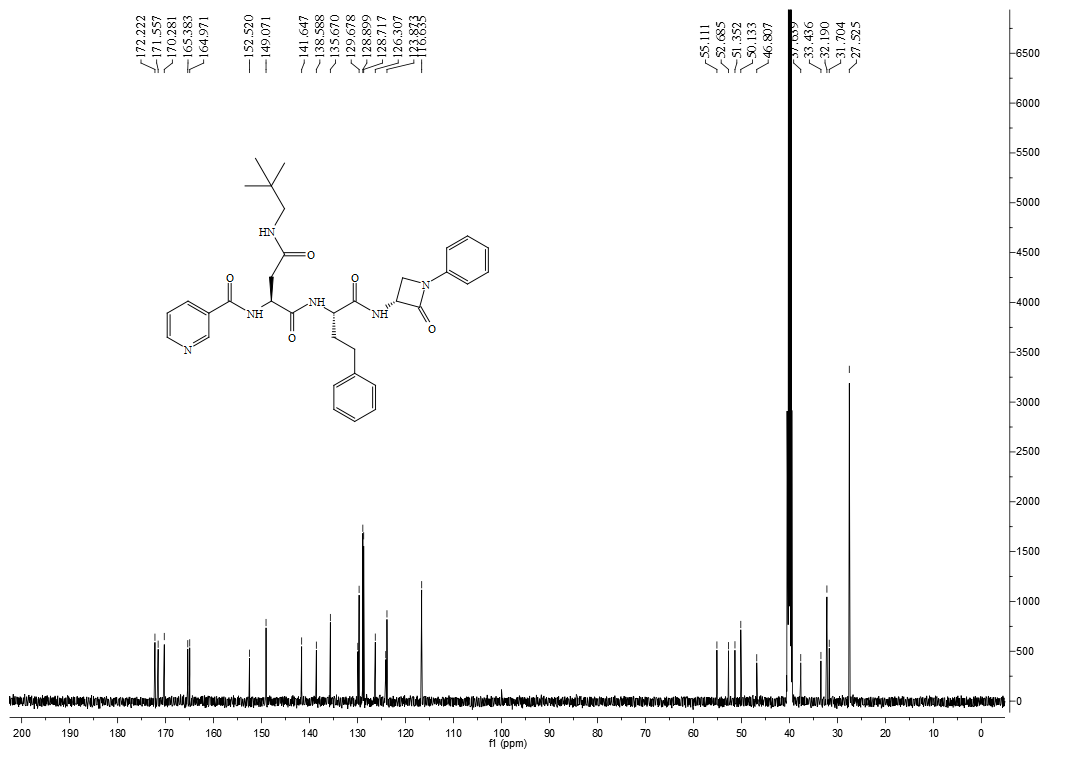


**Figure S2**. ^13^C NMR spectrum of compound **64** in DMSO-*d*_6_

_
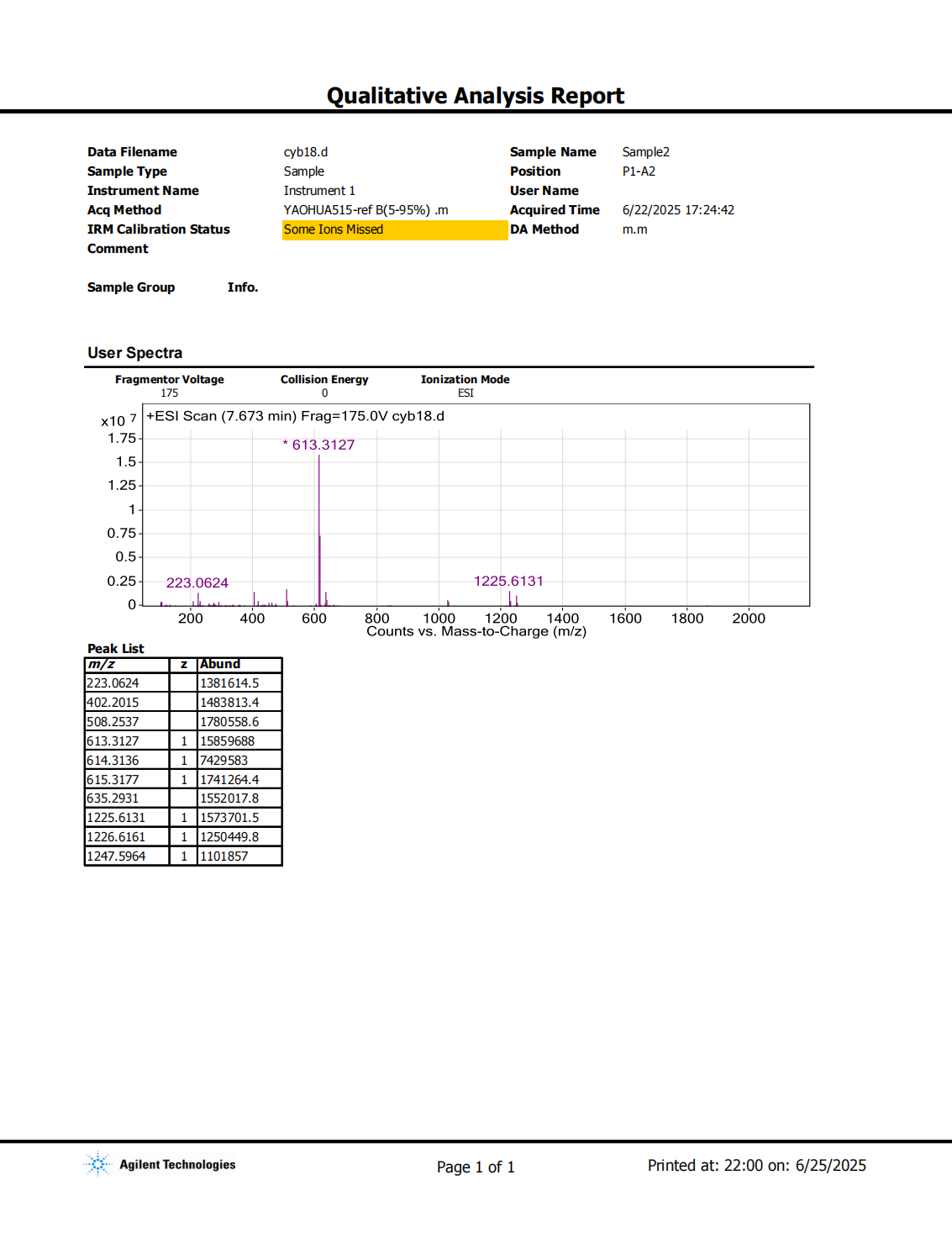
_

**Figure S3**. HRMS spectrum of compound **64**

_
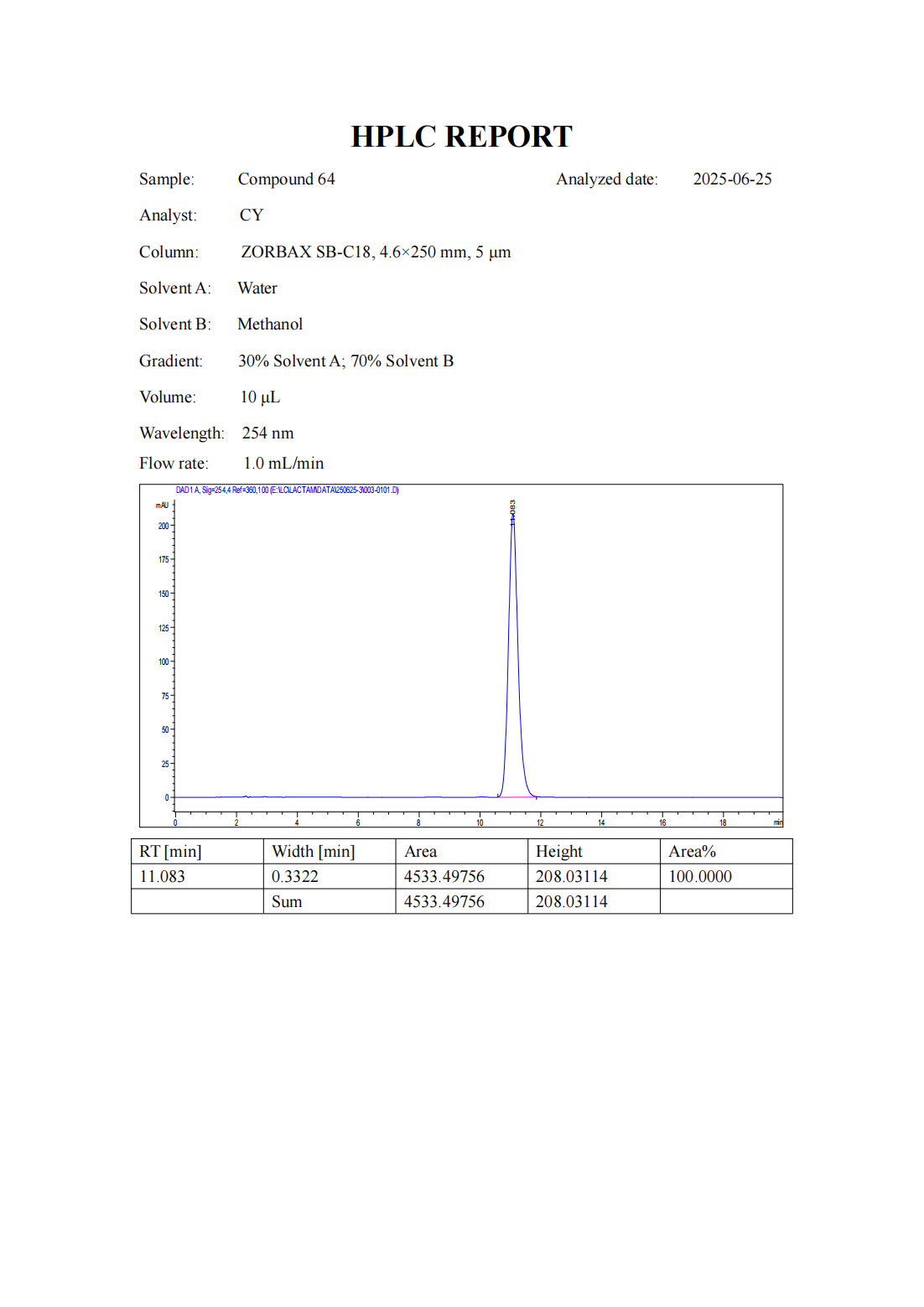
_

**Figure S4**. Chromatogram of compound **64**


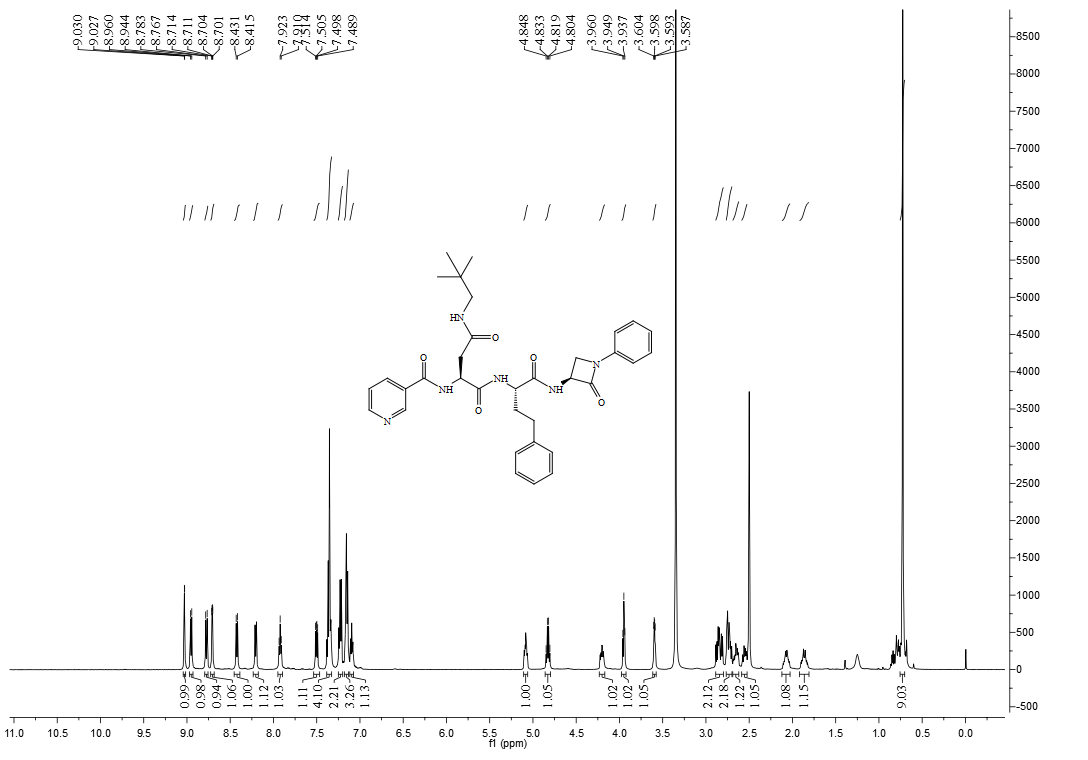


**Figure S5**. ^1^H NMR spectrum of compound **65** in DMSO-*d*_6_


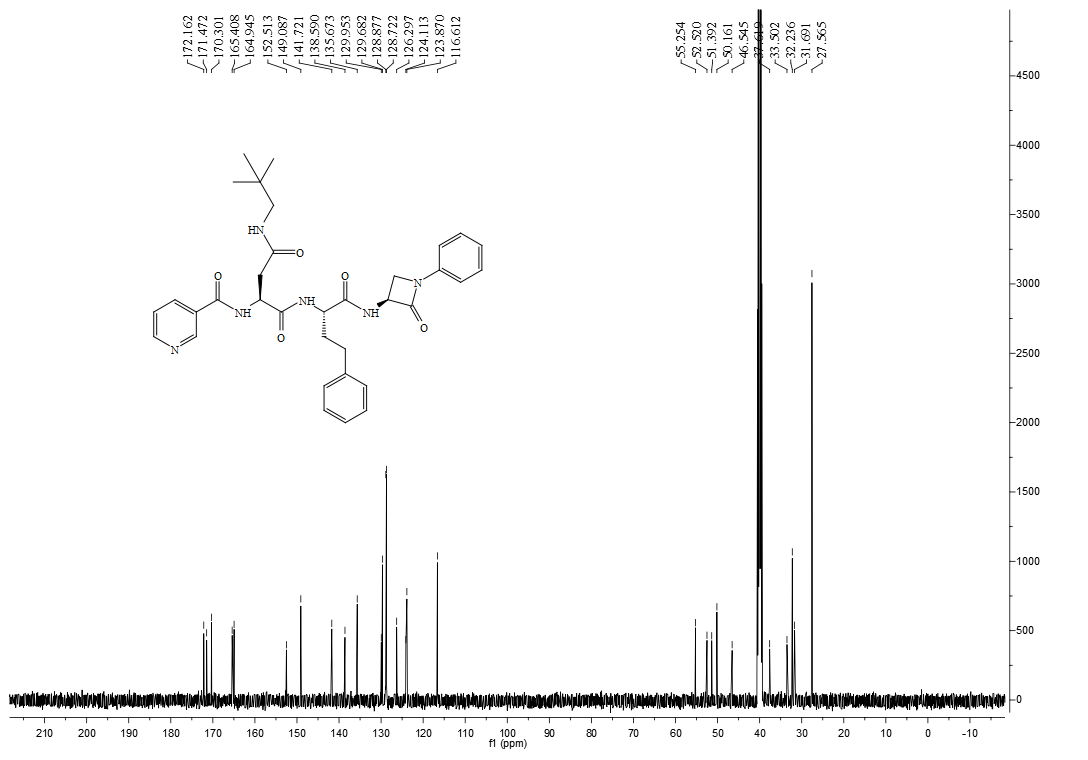


**Figure S6**. ^13^C NMR spectrum of compound **65** in DMSO-*d*_6_


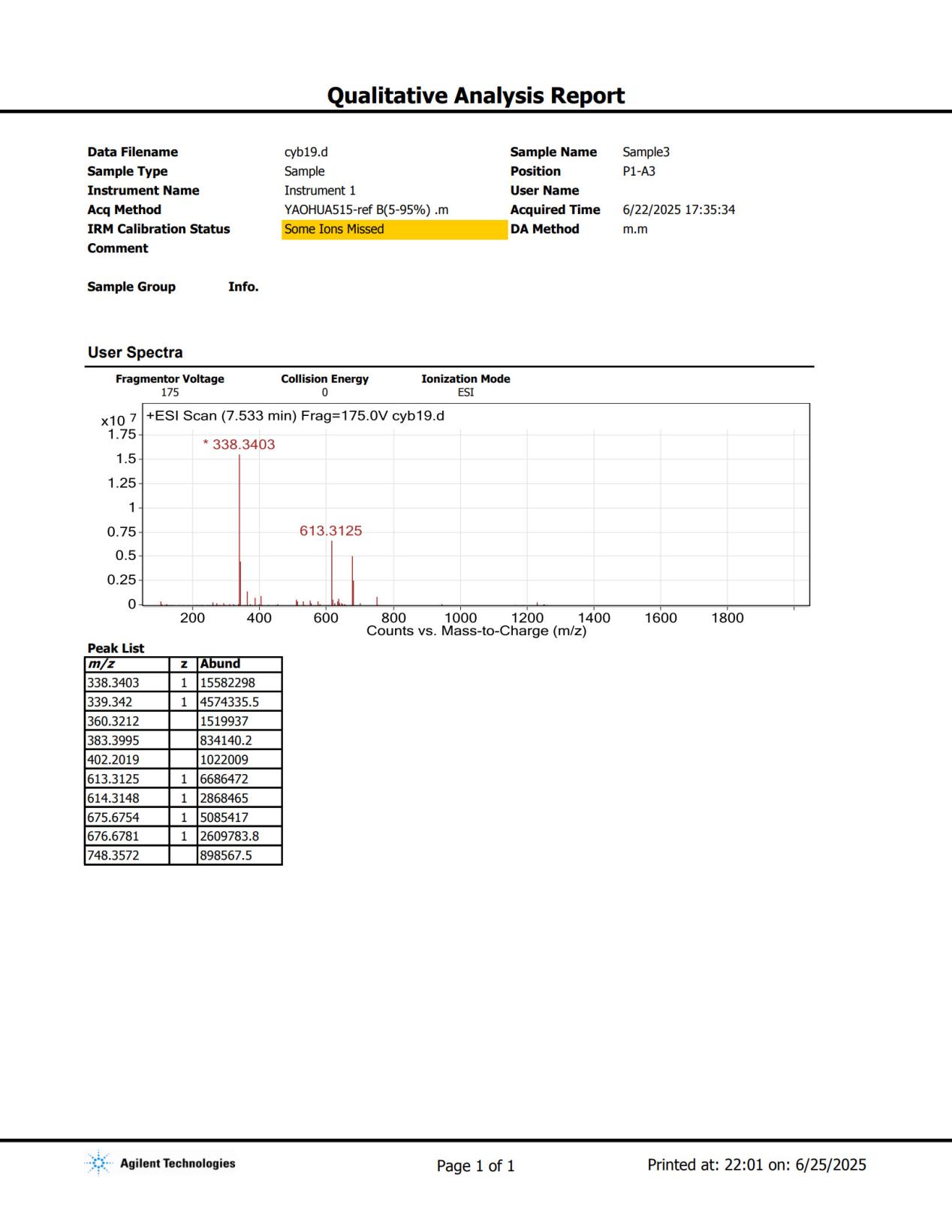


**Figure S7**. HRMS spectrum of compound **65**

**
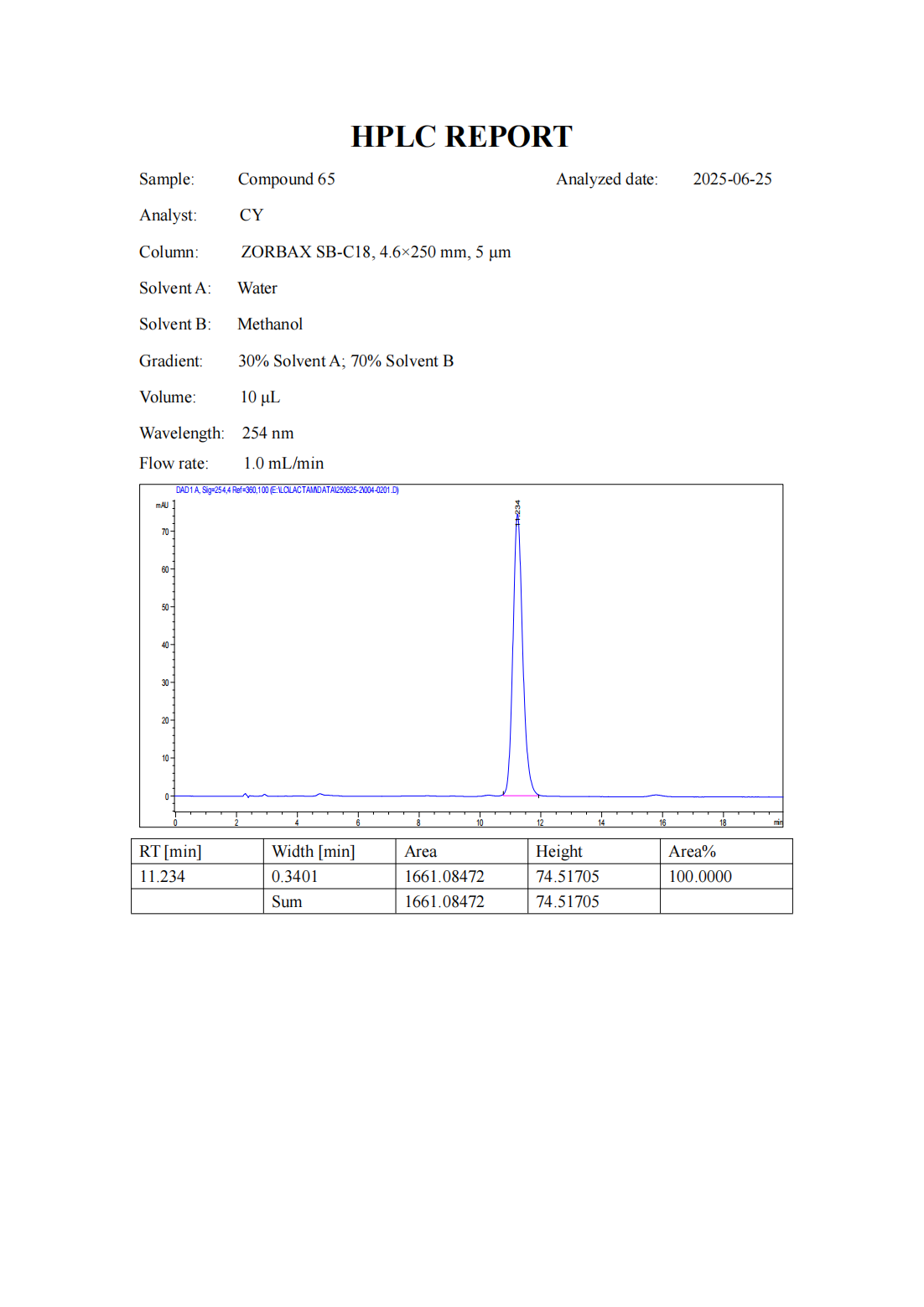
**

**Figure S8**. Chromatogram of compound **65**


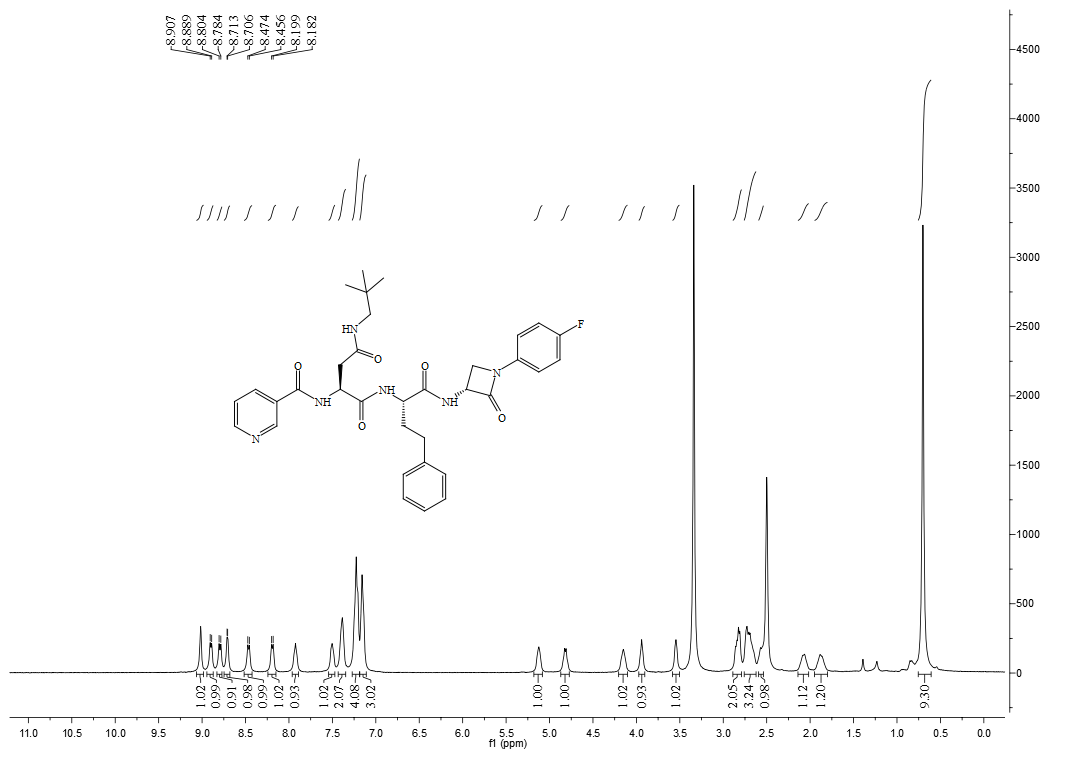


**Figure S9**. ^1^H NMR spectrum of compound **66** in DMSO-*d*_6_


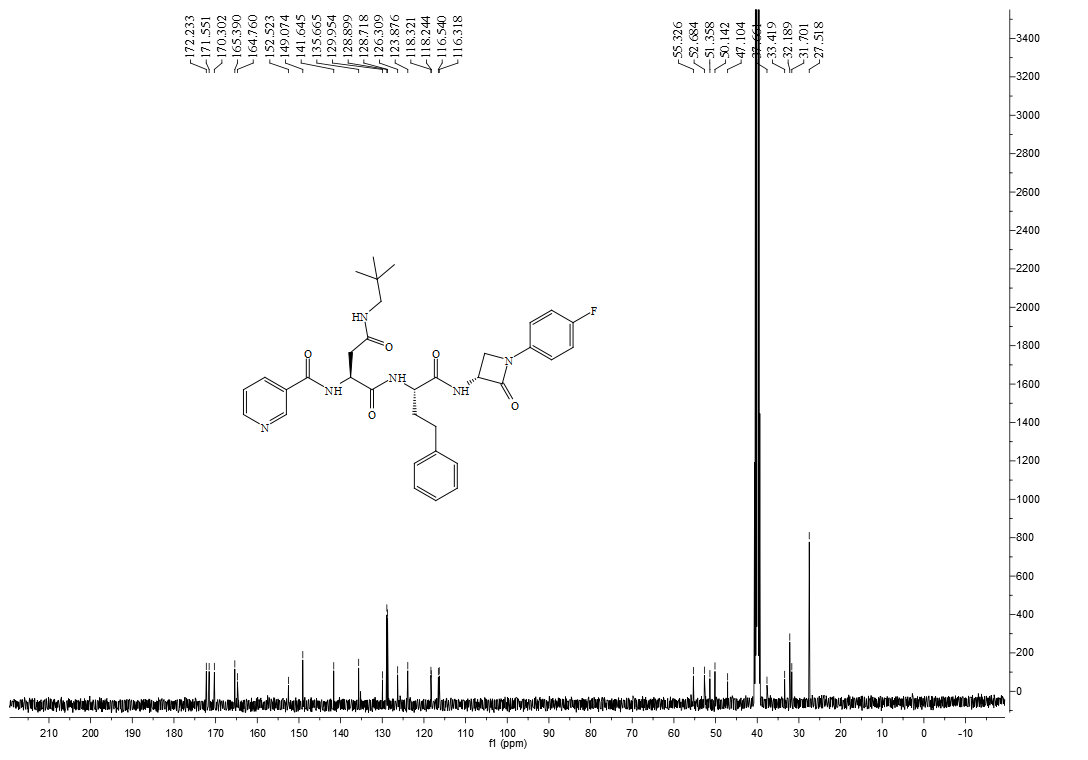


**Figure S10**. ^13^C NMR spectrum of compound **66** in DMSO-*d*_6_


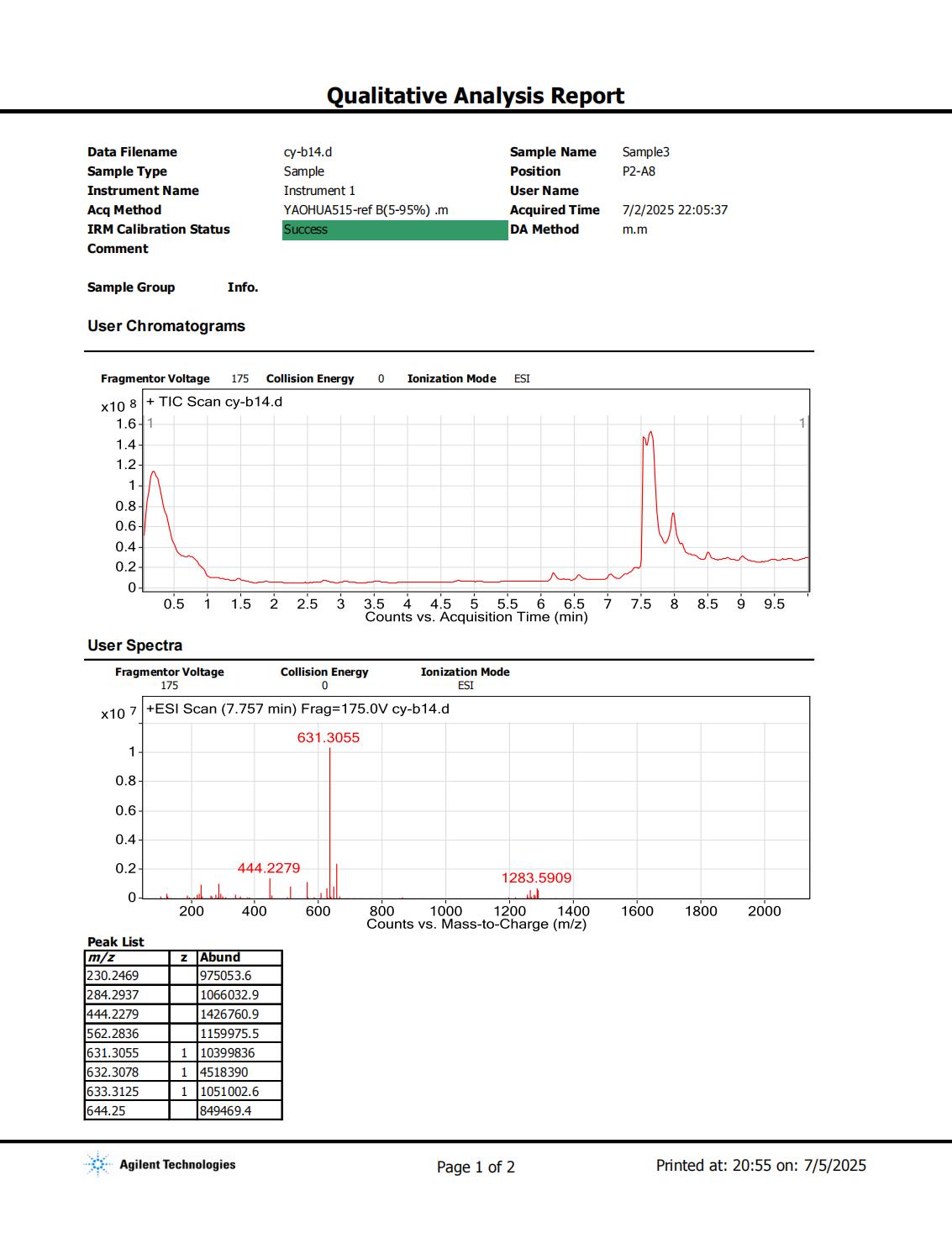


**Figure S11**. HRMS spectrum of compound **66**

**
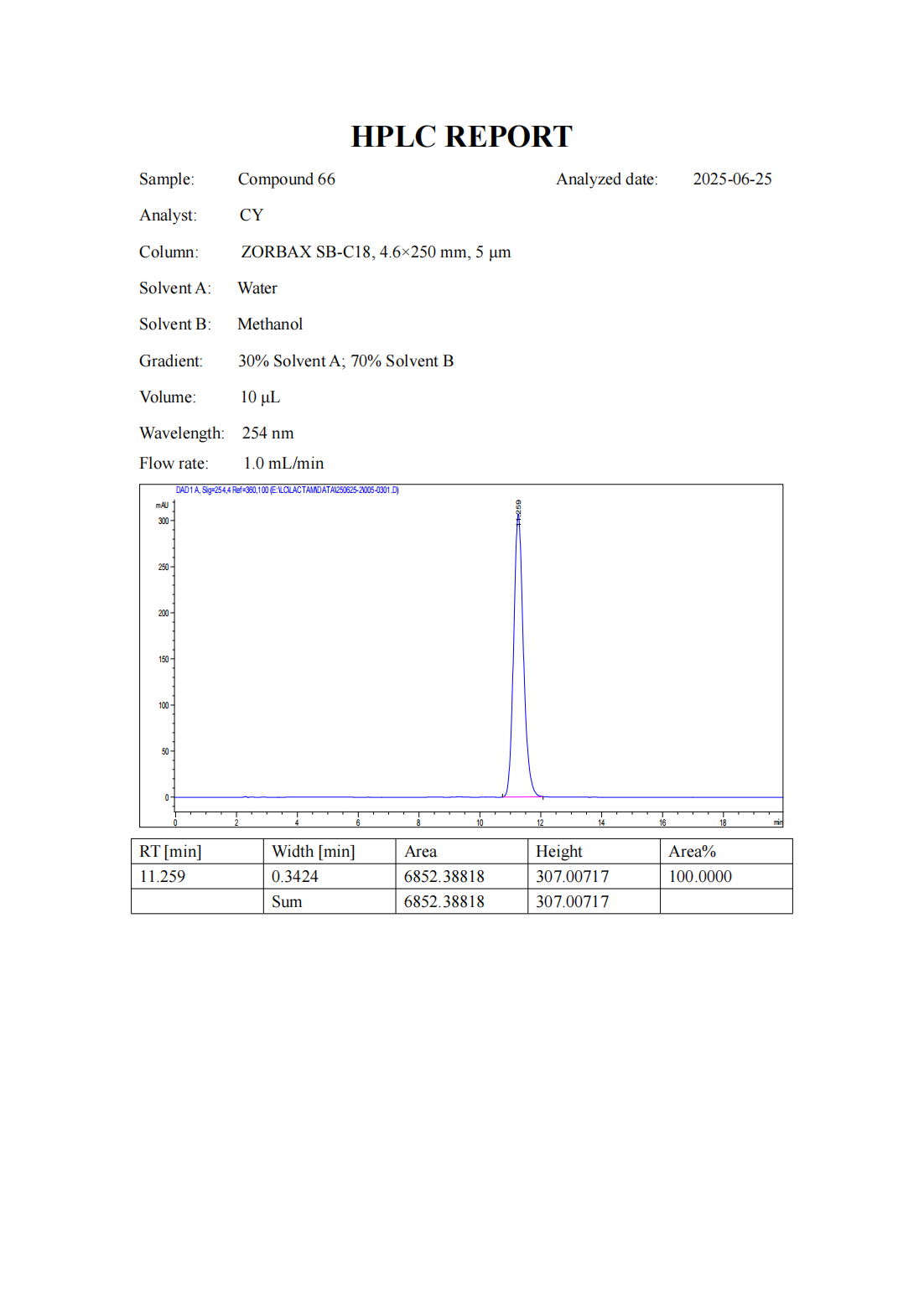
**

**Figure S12**. Chromatogram of compound **66**


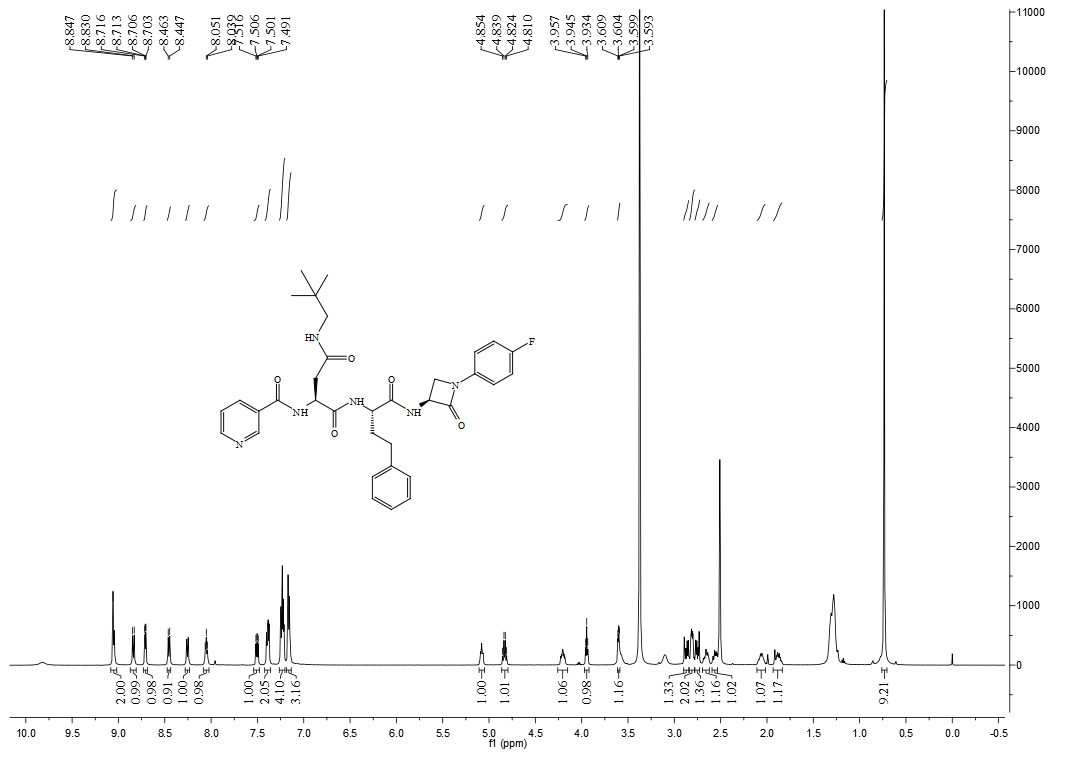


**Figure S13**. ^1^H NMR spectrum of compound **67** in DMSO-*d*_6_


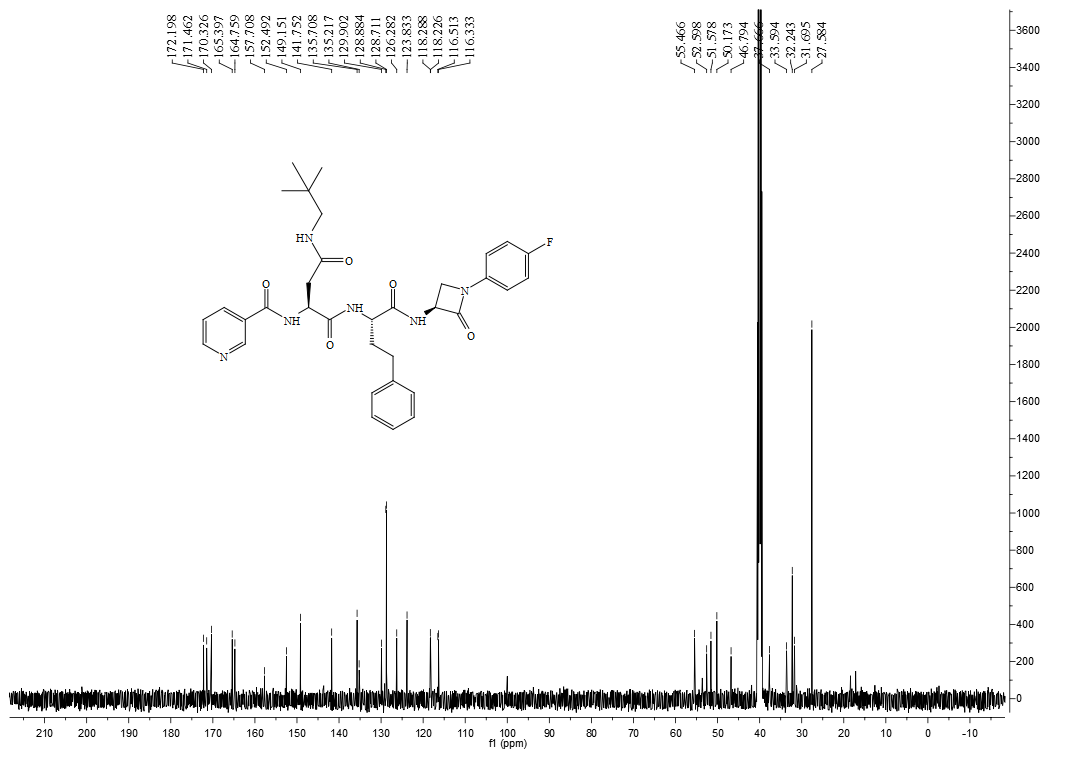


**Figure S14**. ^13^C NMR spectrum of compound **67** in DMSO-*d*_6_

_
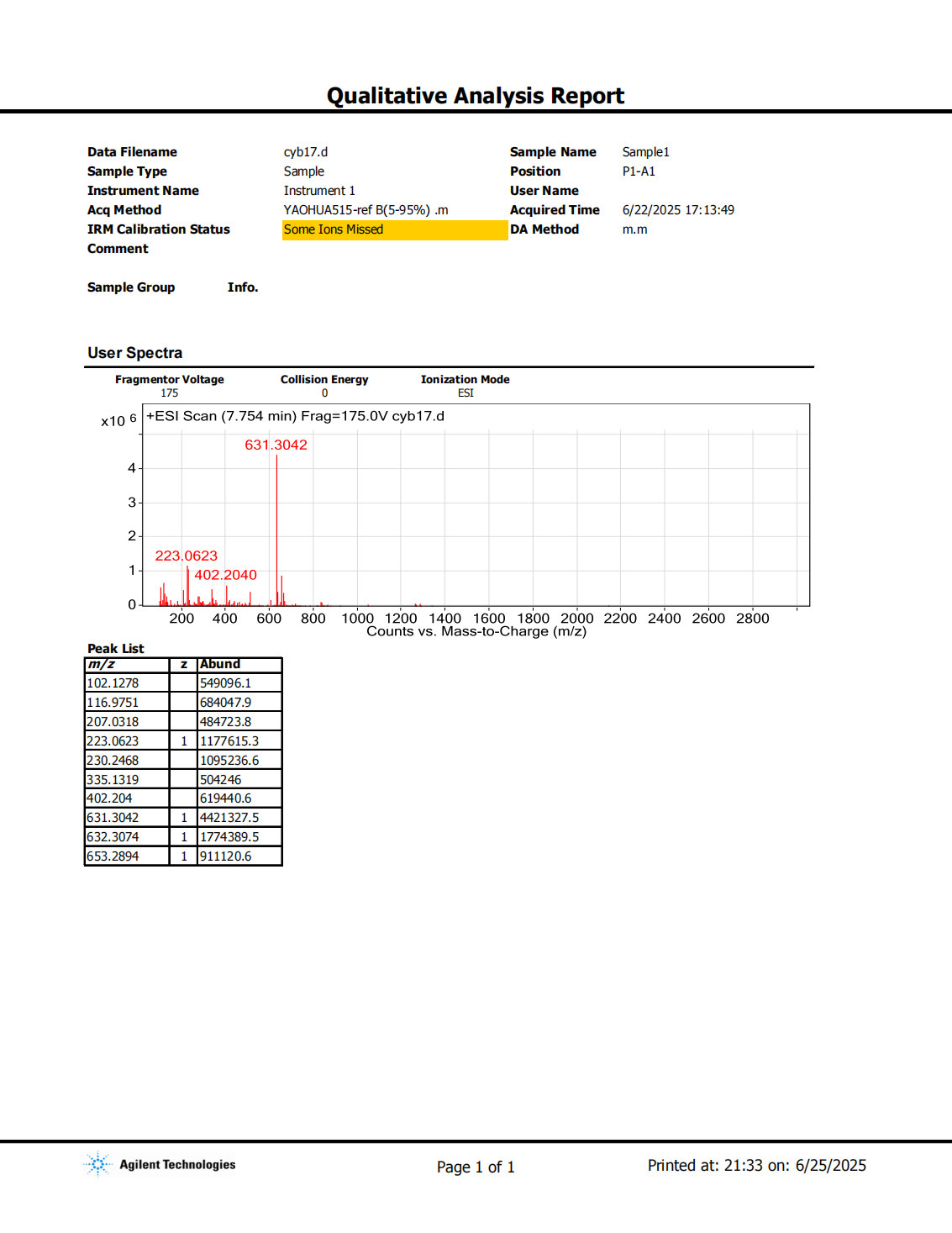
_

**Figure S15**. HRMS spectrum of compound **67**

**
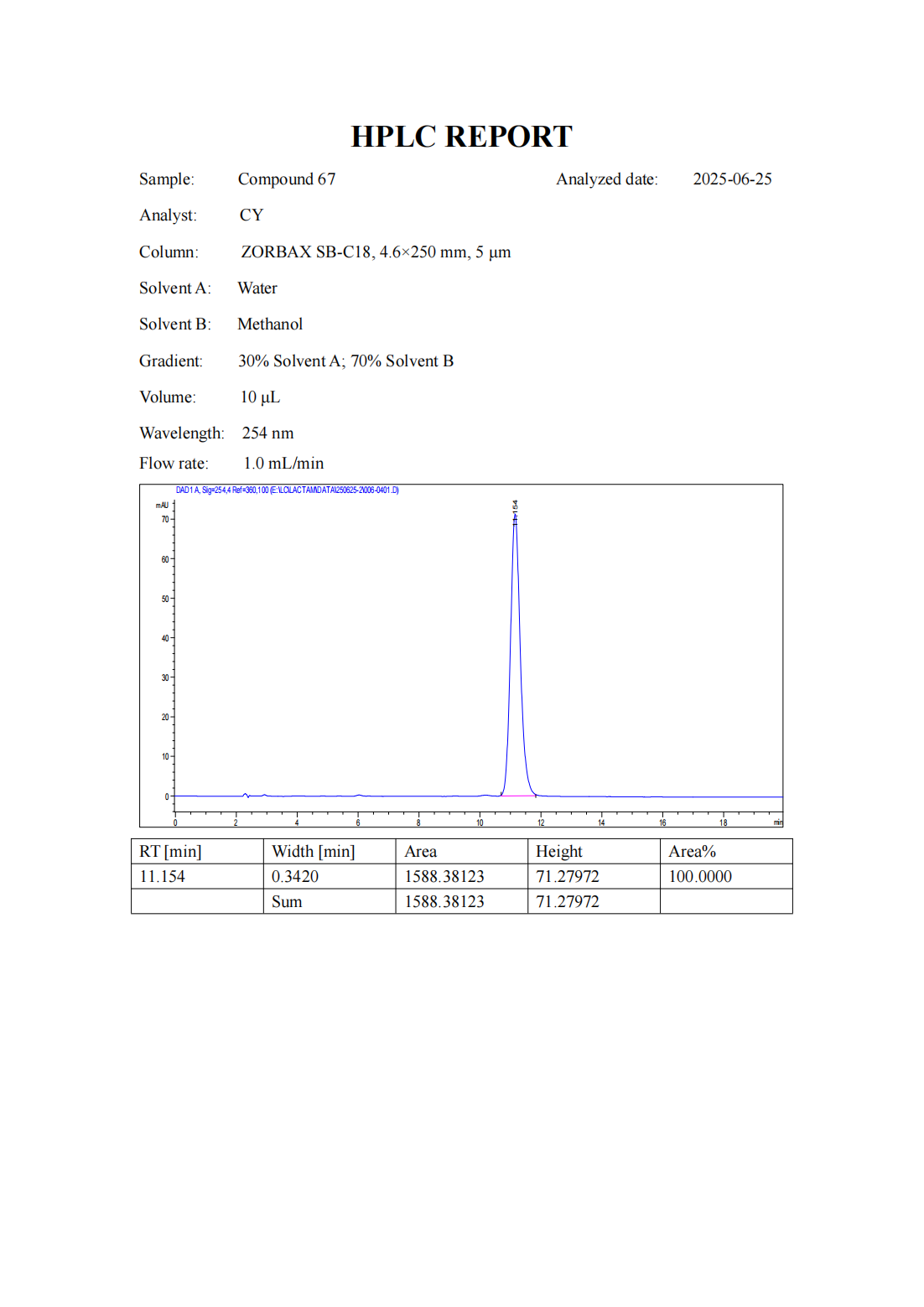
**

**Figure S16**. Chromatogram of compound **67**


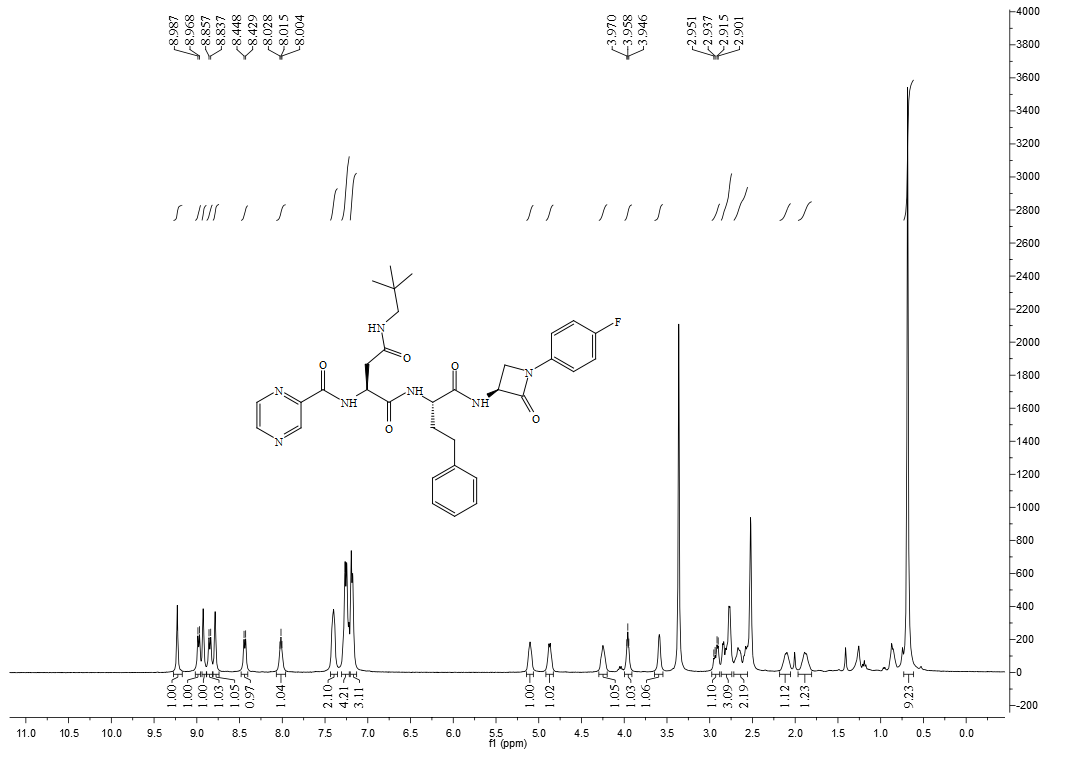


**Figure S17**. ^1^H NMR spectrum of compound **68** in DMSO-*d*_6_


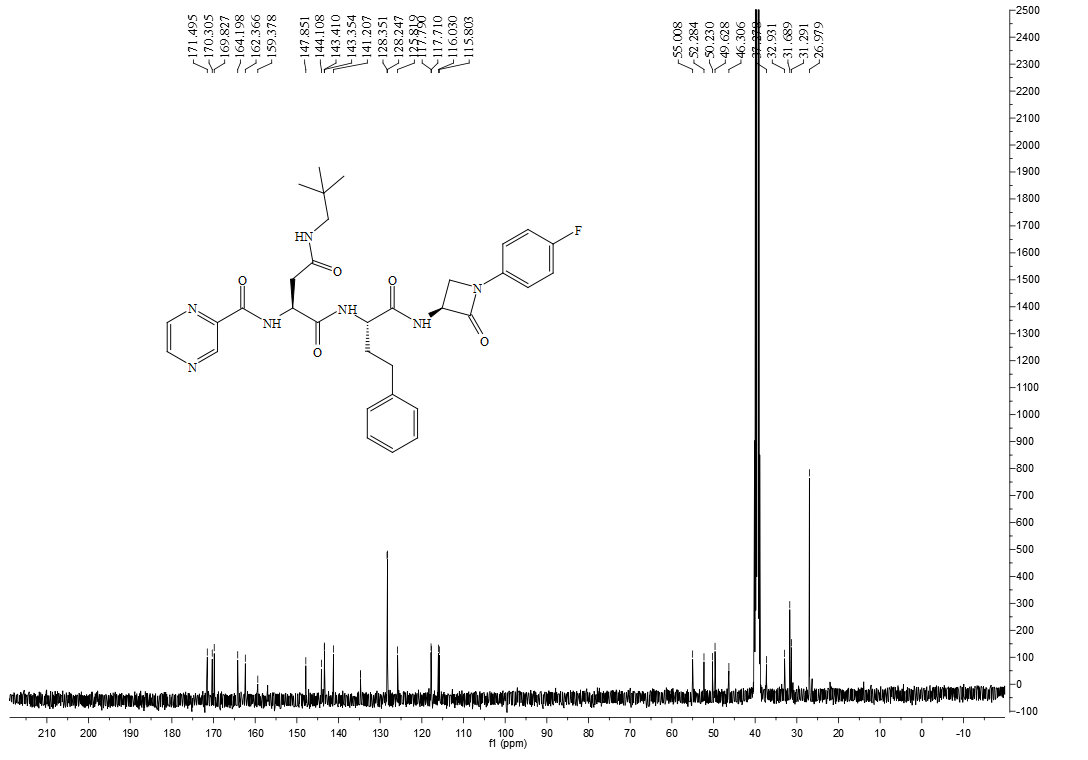


**Figure S18**. ^13^C NMR spectrum of compound **68** in DMSO-*d*_6_

_
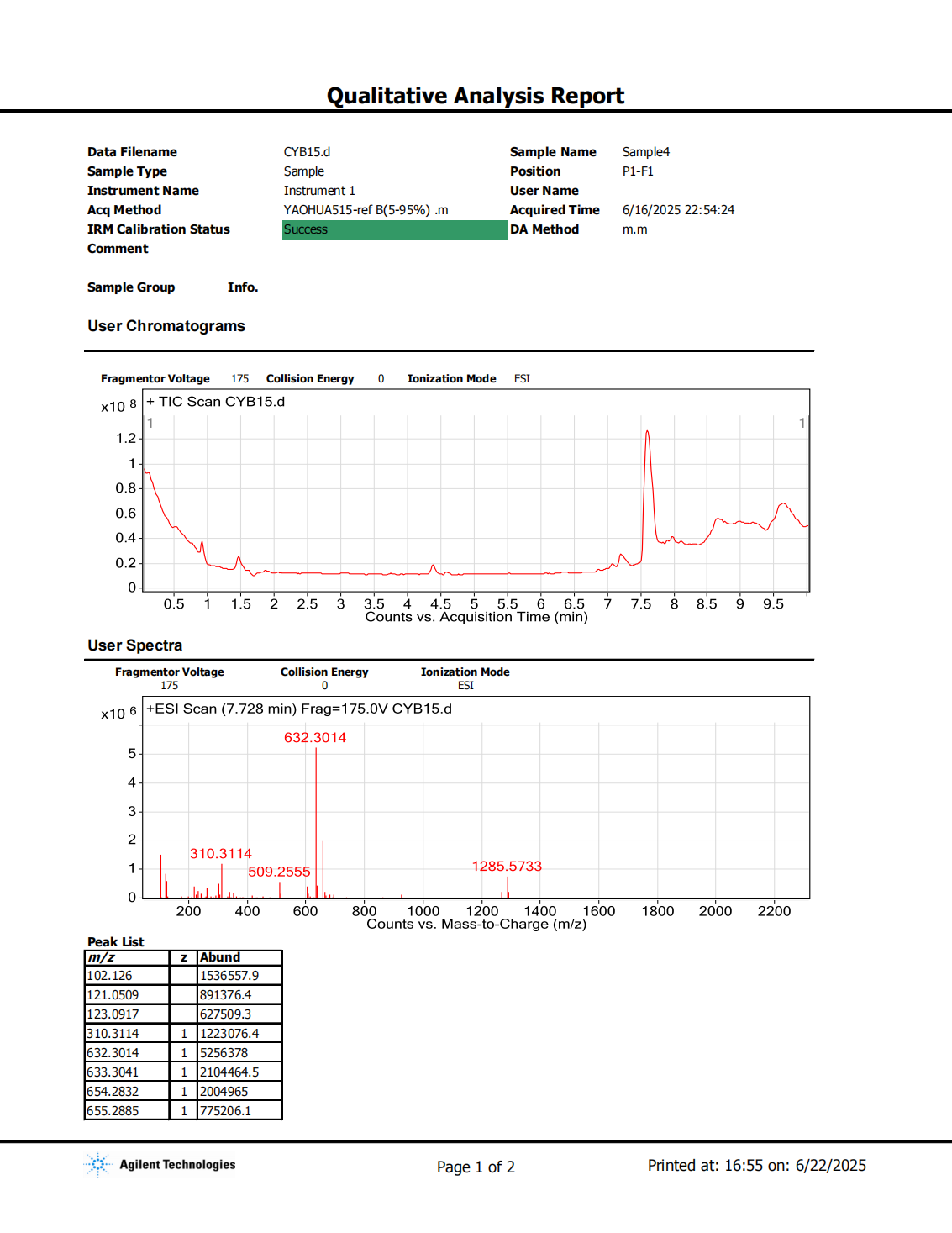
_

**Figure S19**. HRMS spectrum of compound **68**

**
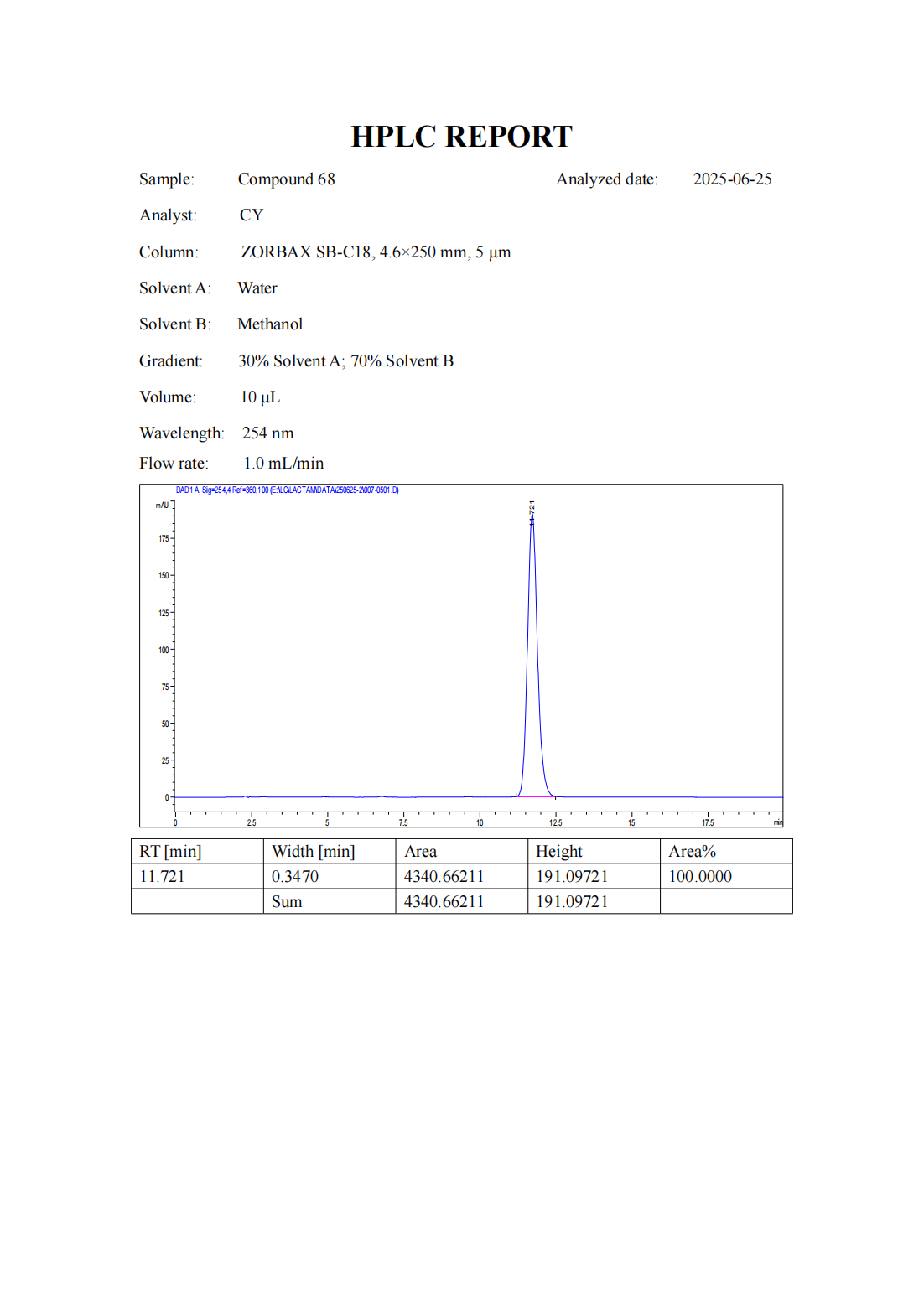
**

**Figure S20**. Chromatogram of compound **68**


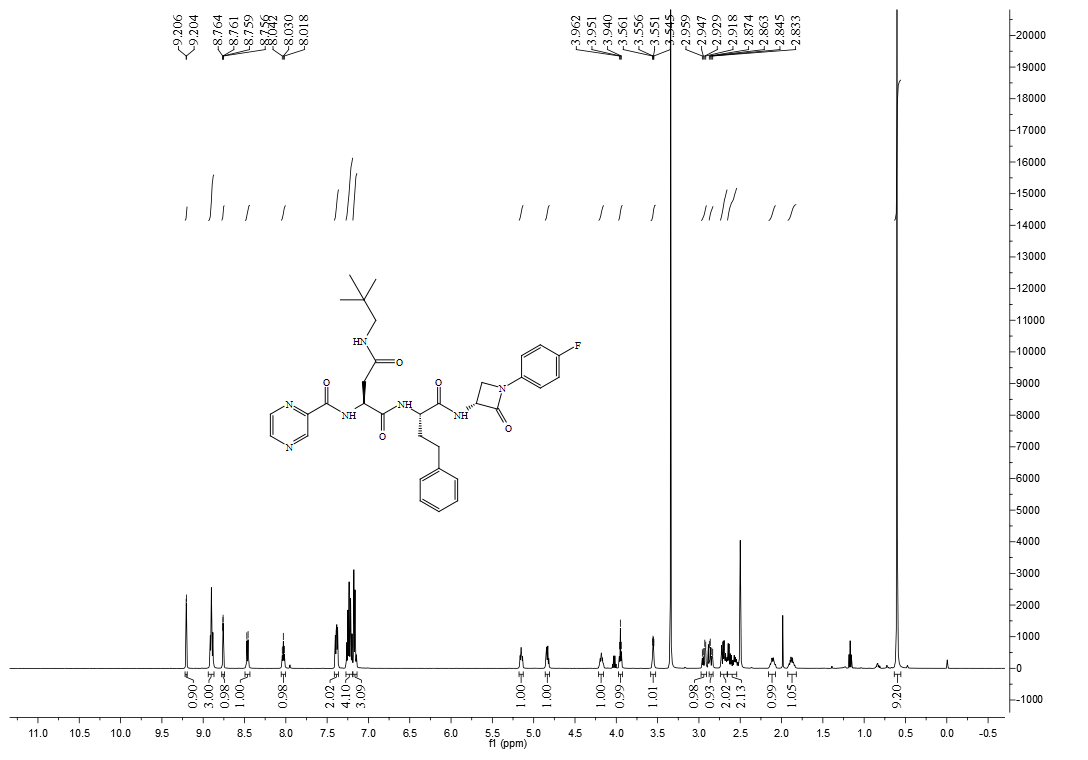


**Figure S21**. ^1^H NMR spectrum of compound **69** in DMSO-*d*_6_


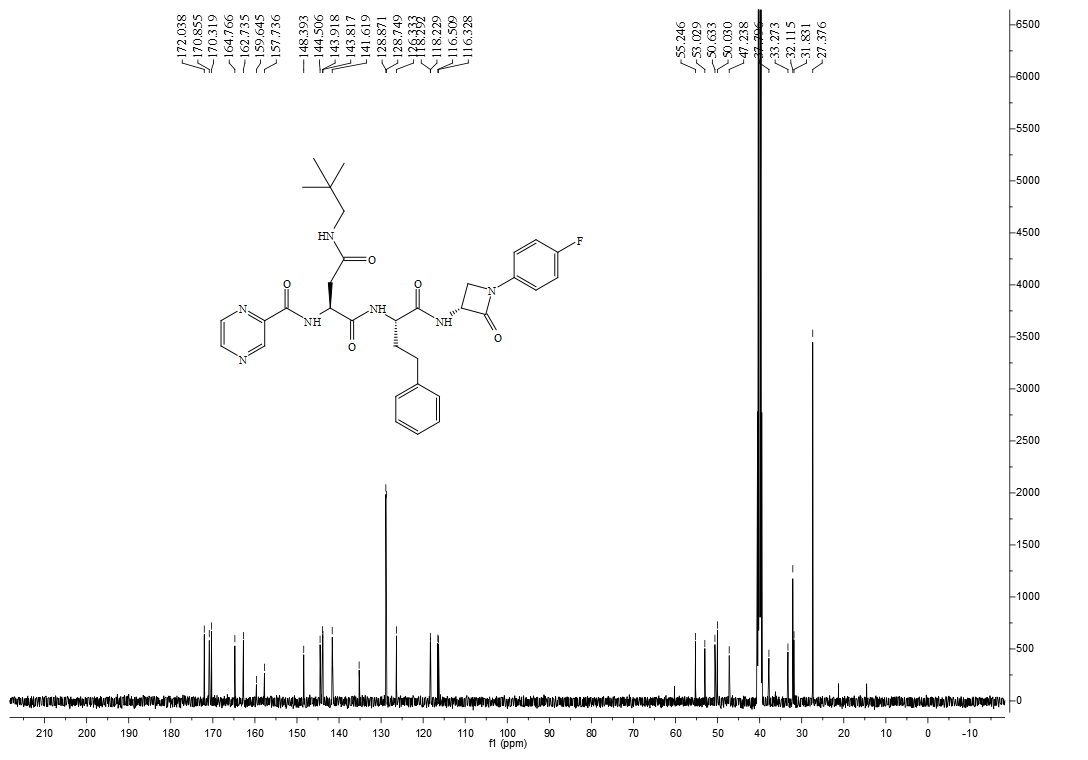


**Figure S22**. ^13^C NMR spectrum of compound **69** in DMSO-*d*_6_

*
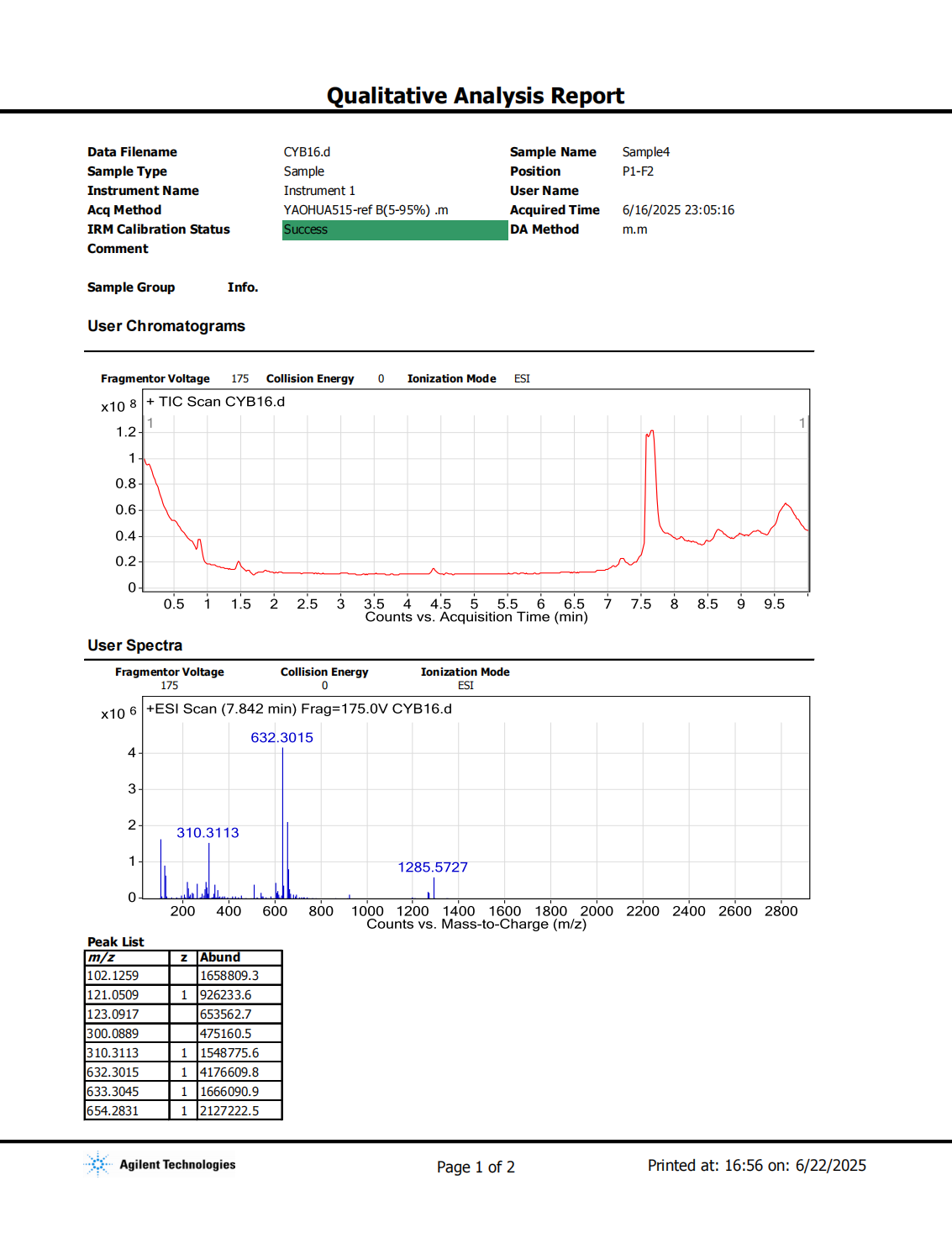
*

**Figure S23**. HRMS spectrum of compound **69**

**
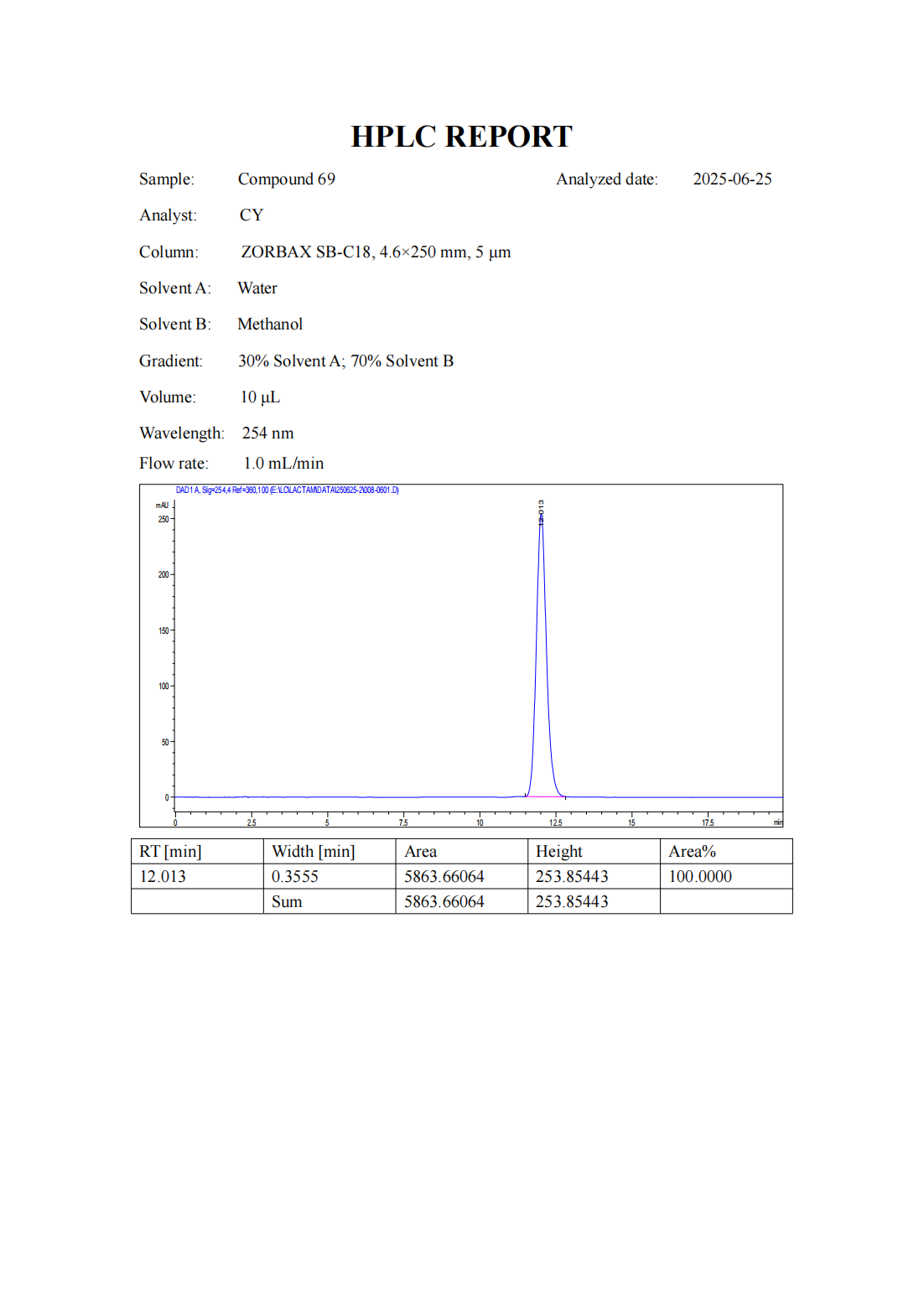
**

**Figure S24**. Chromatogram of compound **69**


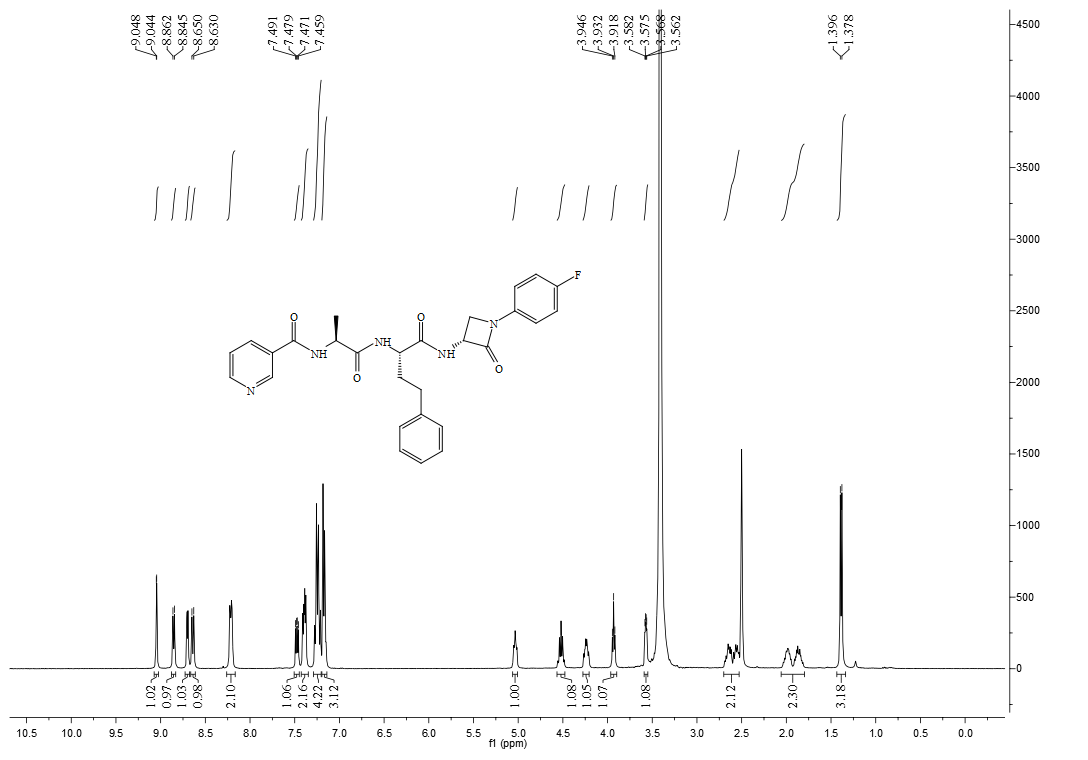


**Figure S25**. ^1^H NMR spectrum of compound **70** in DMSO-*d*_6_


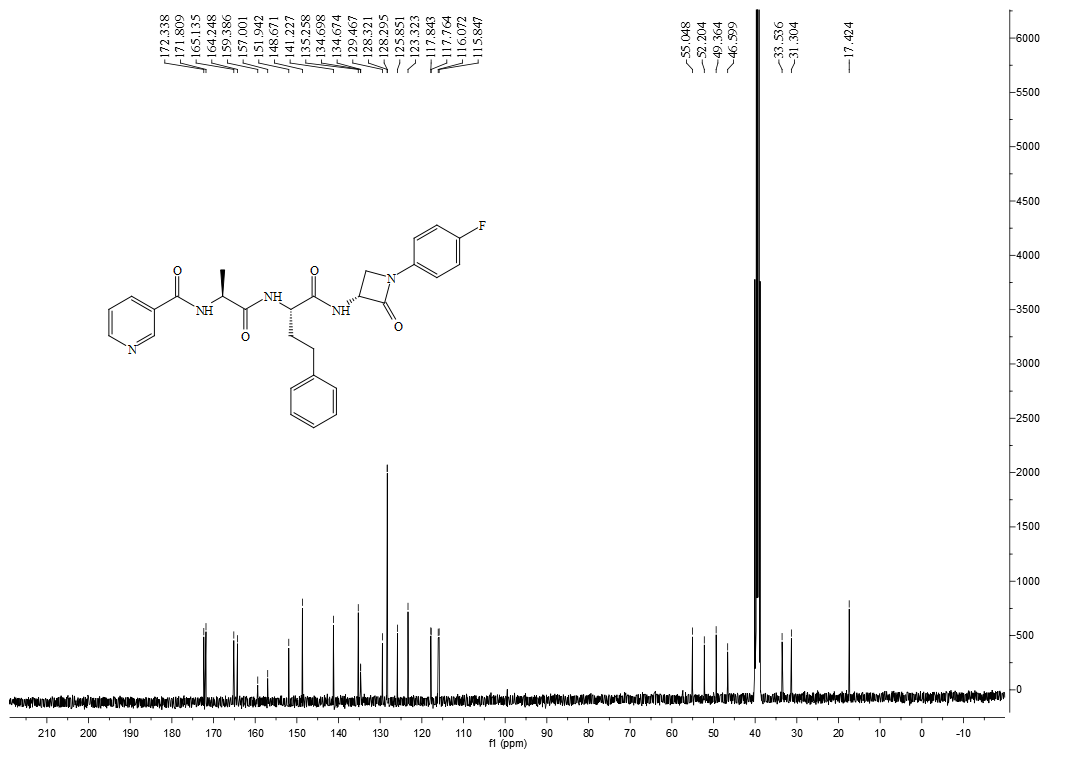


**Figure S26**. ^13^C NMR spectrum of compound **70** in DMSO-*d*_6_

*
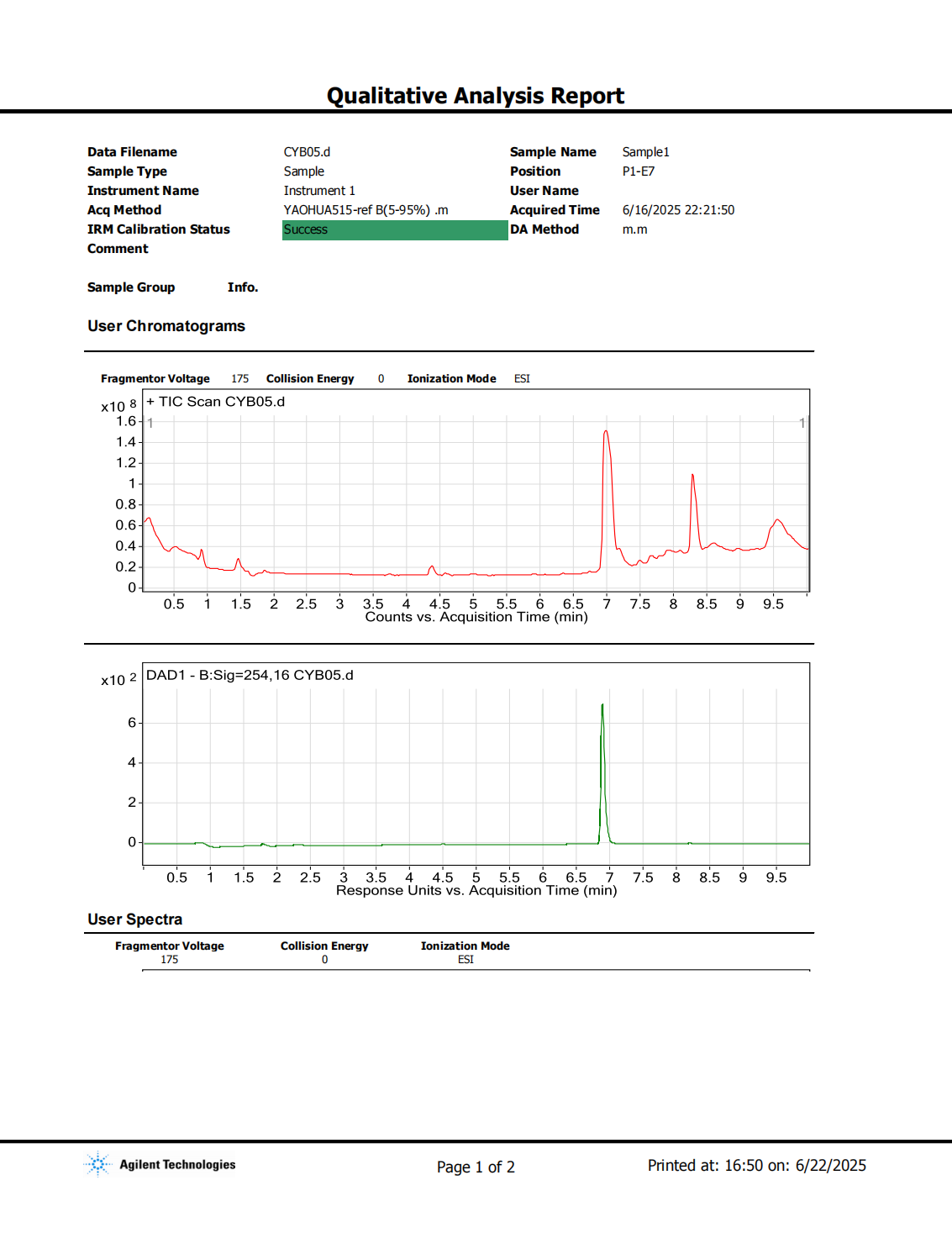
*

*
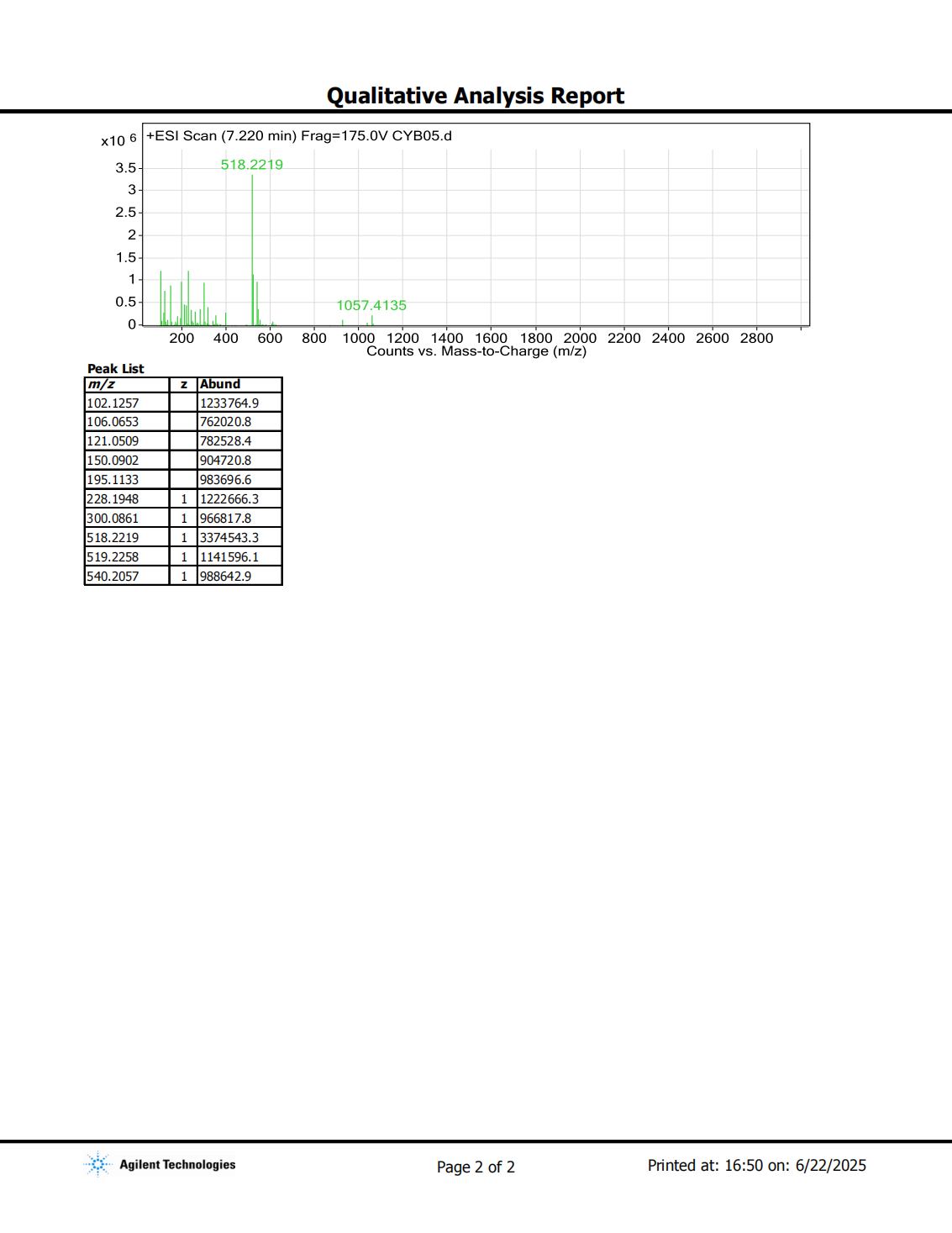
*

**Figure S27**. HRMS spectrum of compound **70**


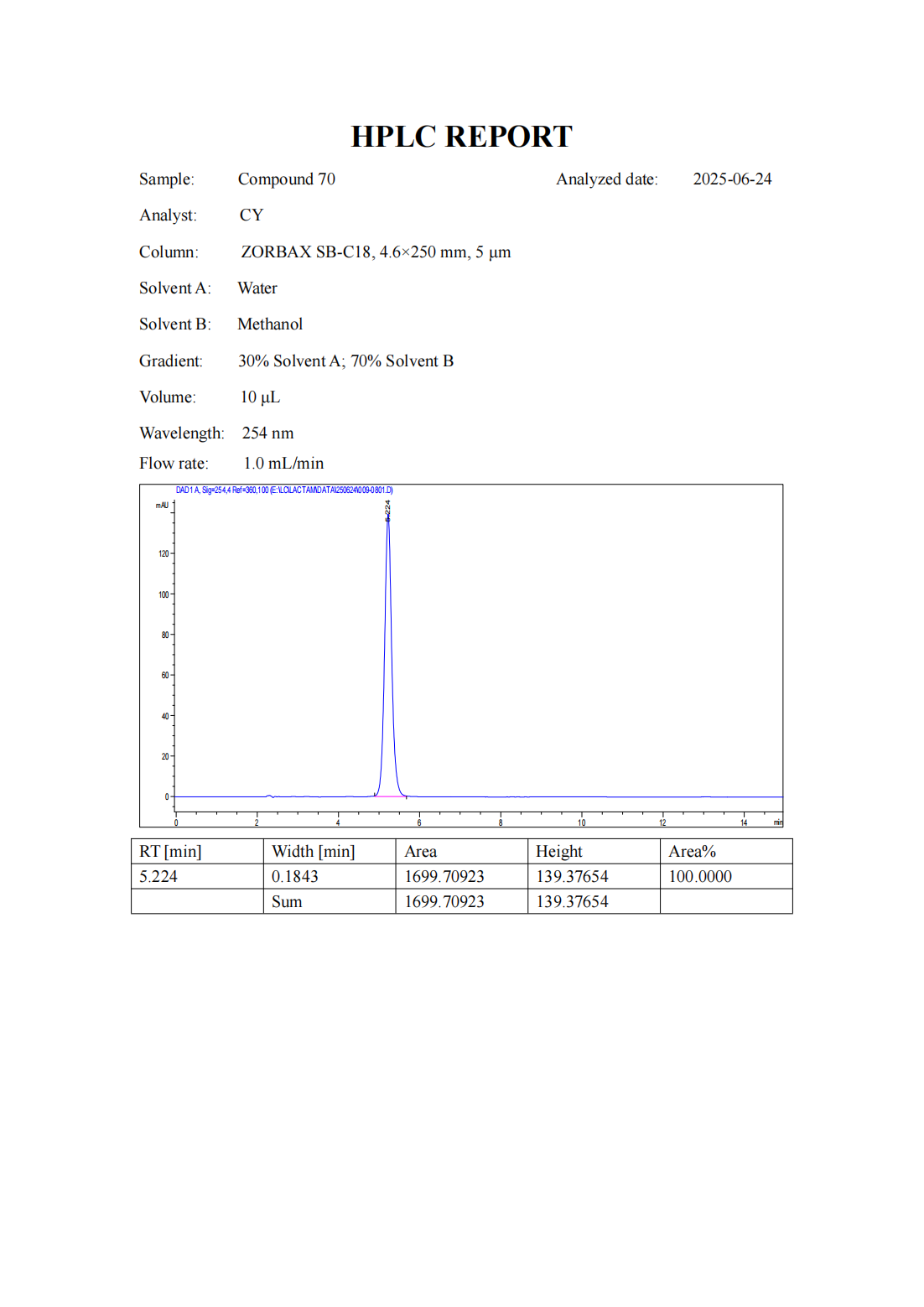


**Figure S28**. Chromatogram of compound **70**


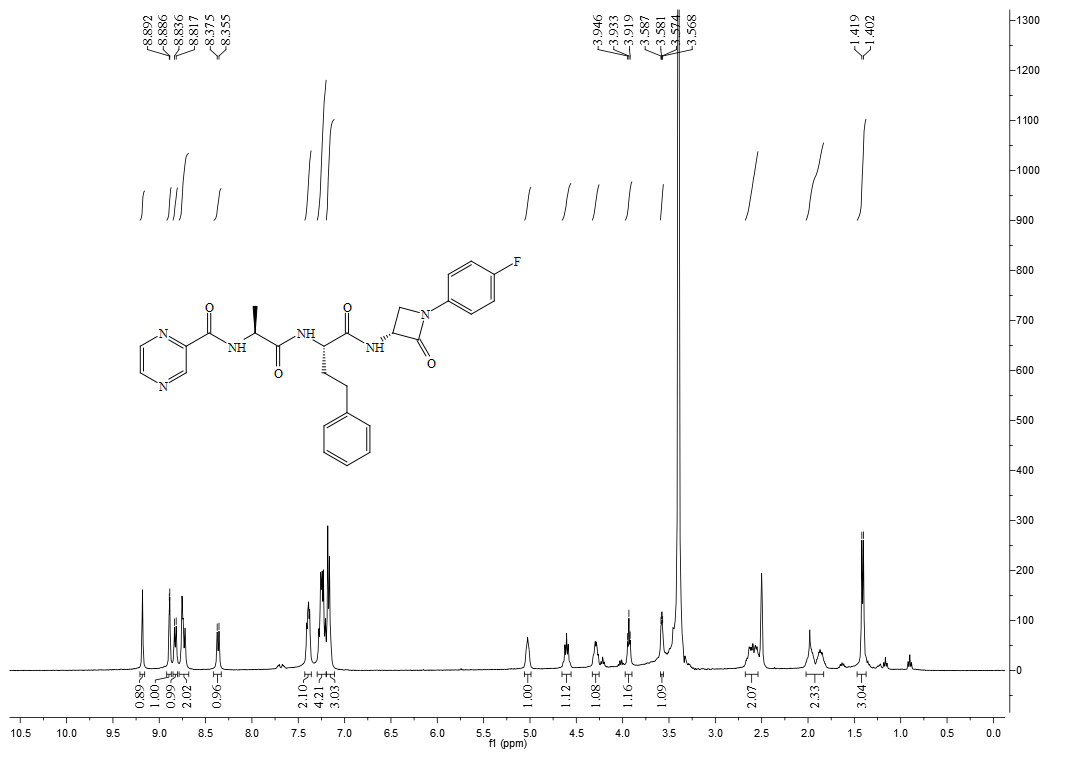


**Figure S29**. ^1^H NMR spectrum of compound **71** in DMSO-*d*_6_


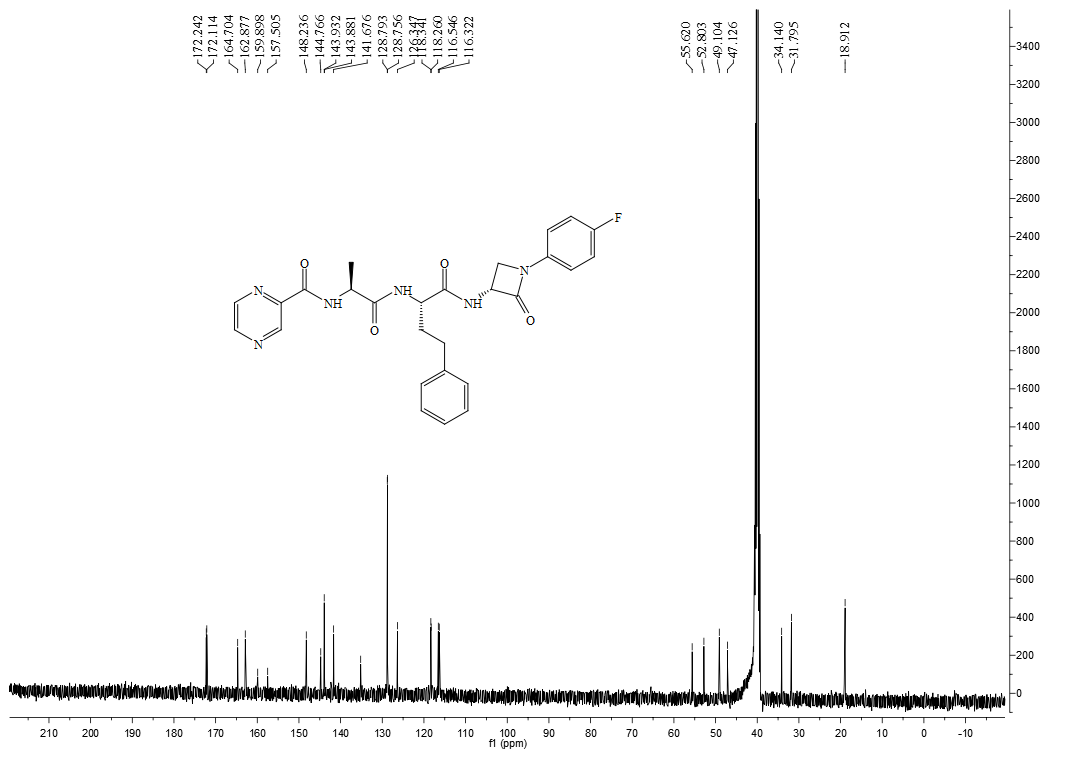


**Figure S30**. ^1^H NMR spectrum of compound **71** in DMSO-*d*_6_


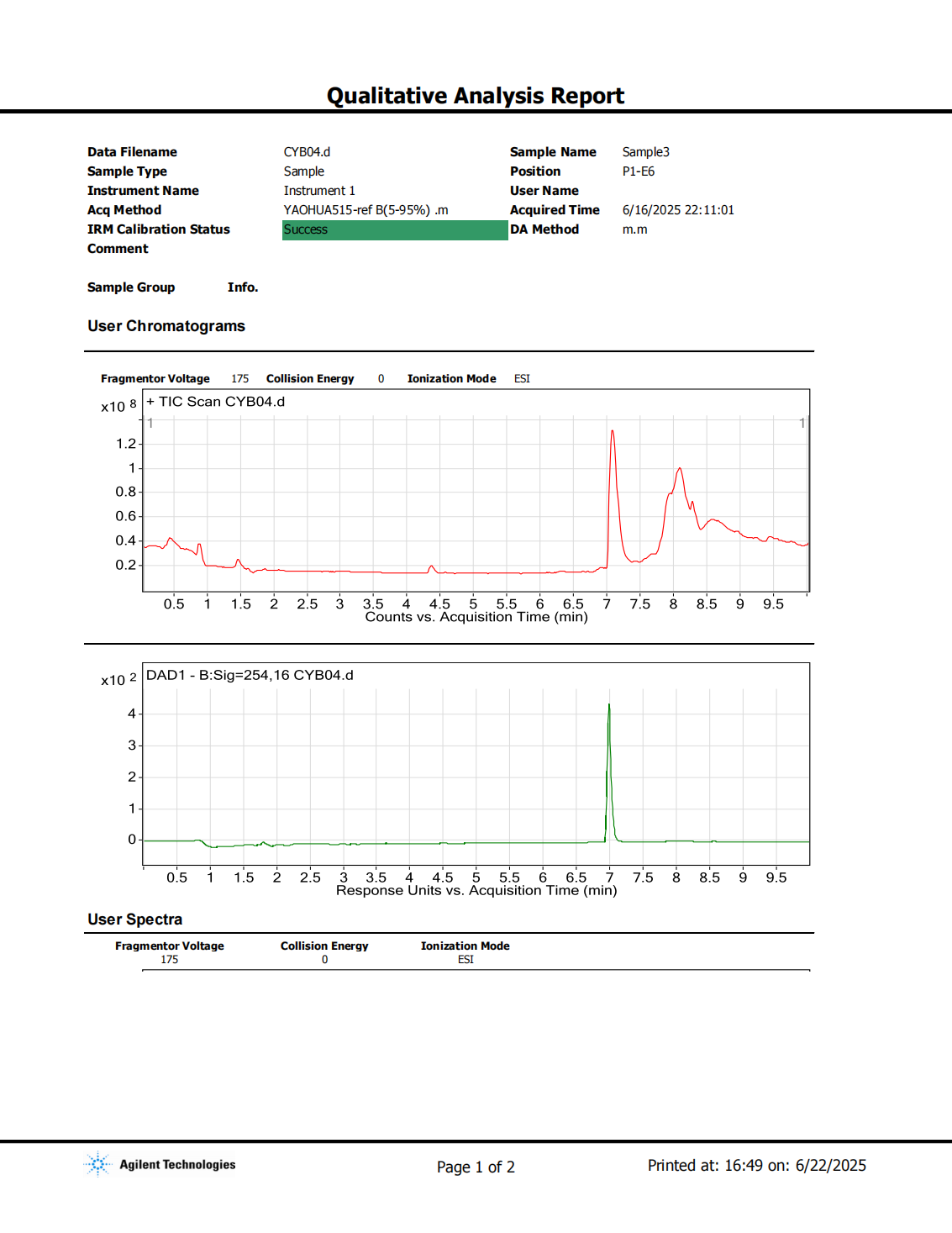


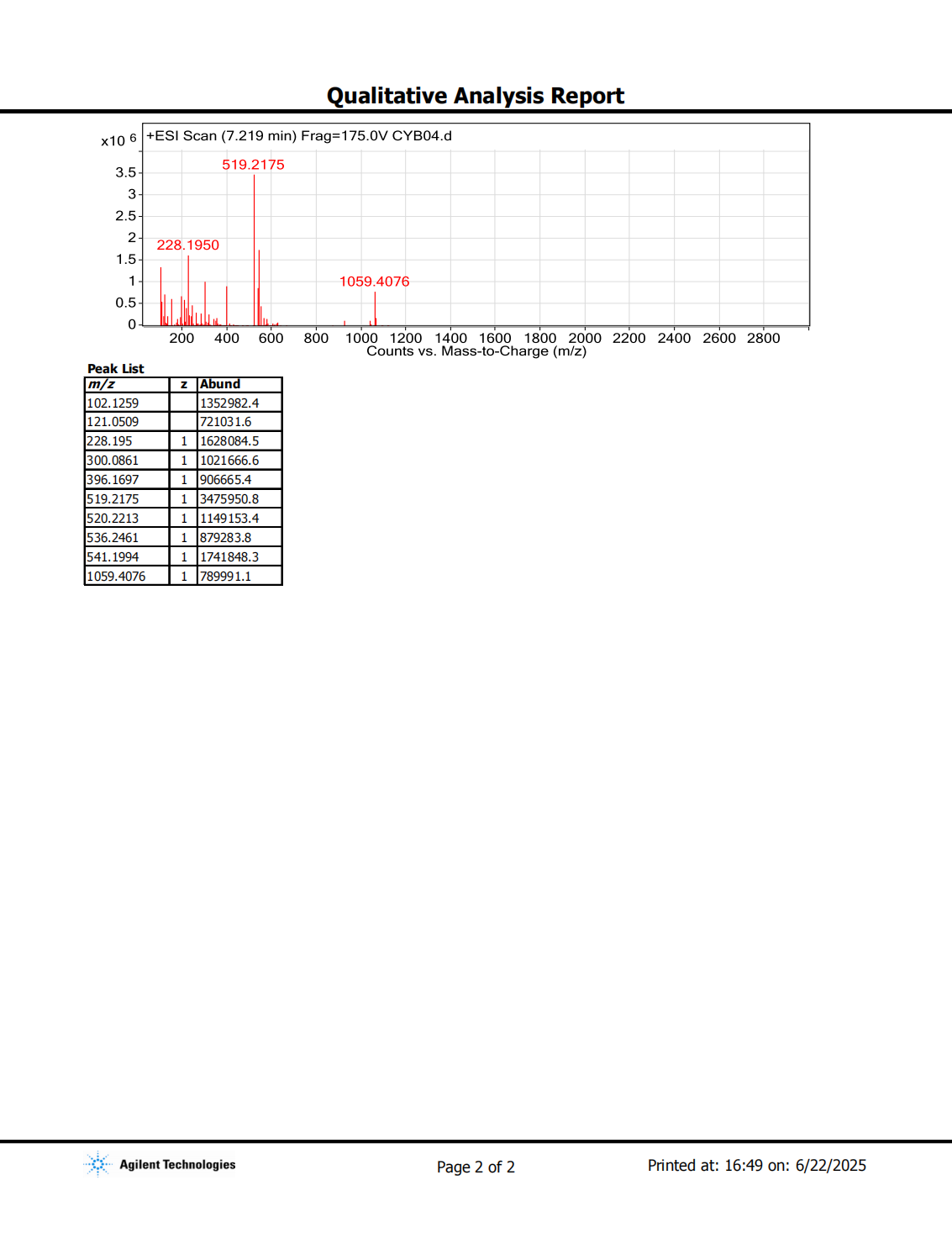


**Figure S31**. HRMS spectrum of compound **71**

**
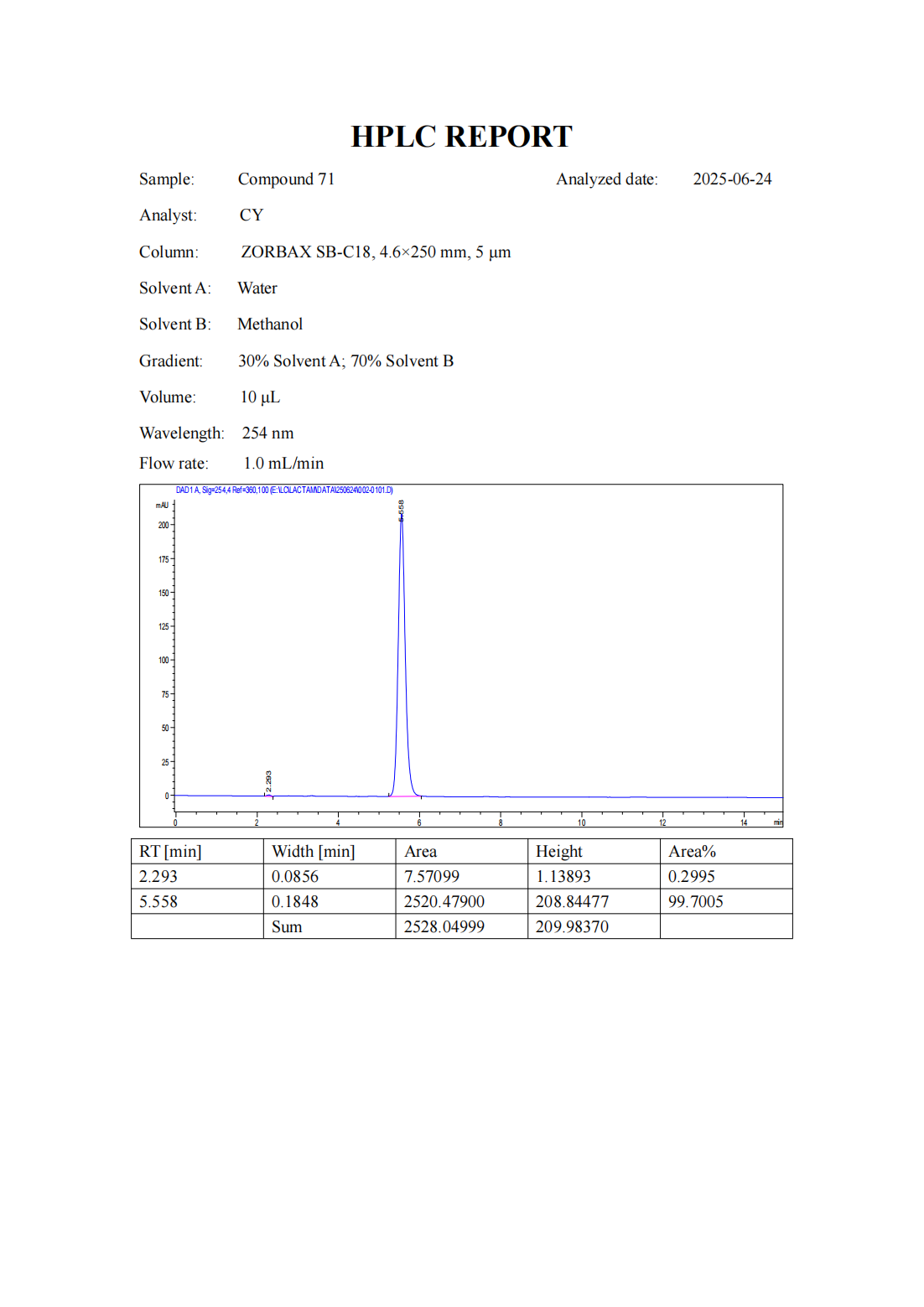
**

**Figure S32**. Chromatogram of compound **71**


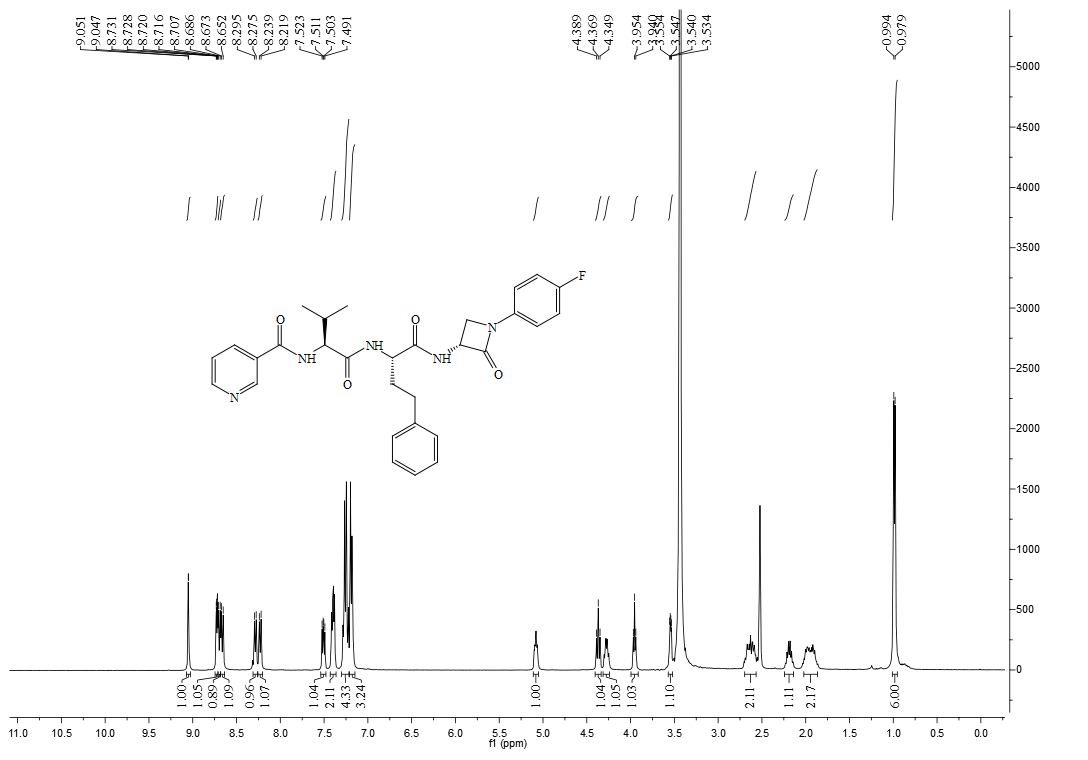


**Figure S33**. ^1^H NMR spectrum of compound **72** in DMSO-*d*_6_


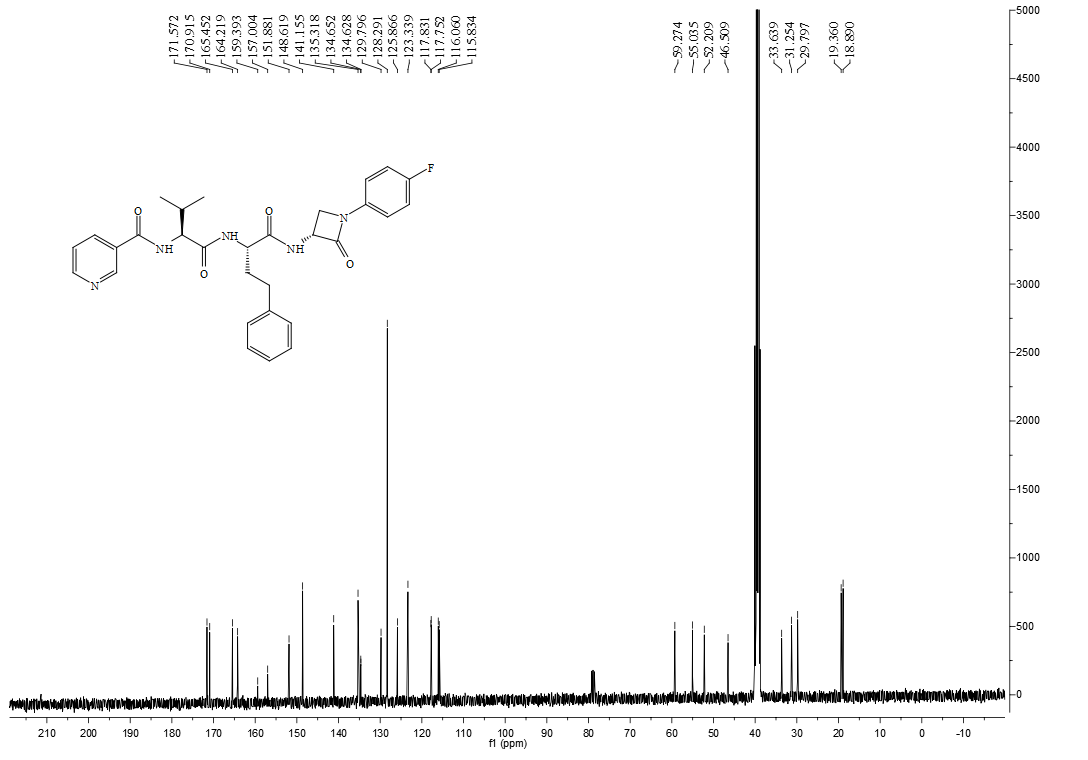


**Figure S34**. ^13^C NMR spectrum of compound **72** in DMSO-*d*_6_


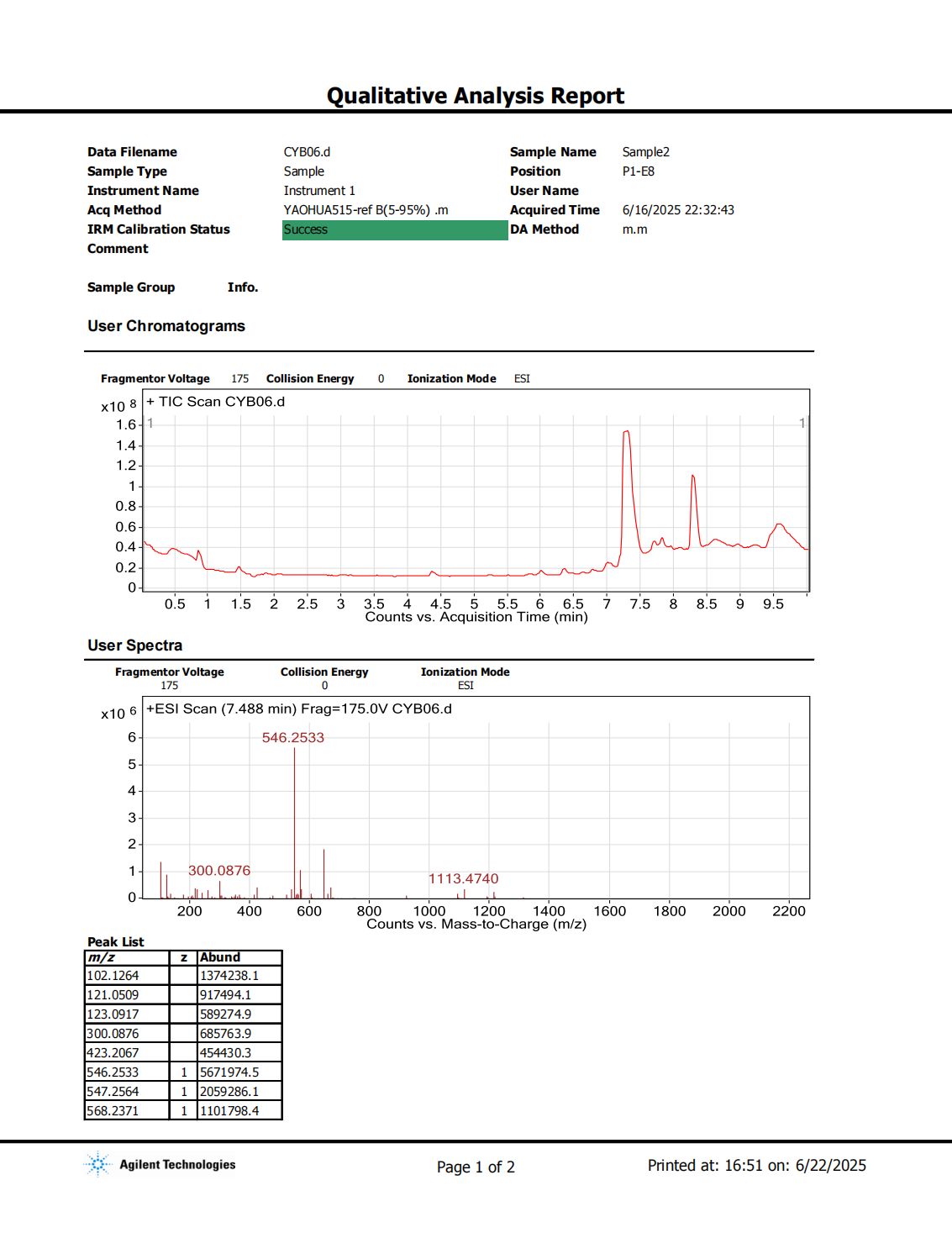


**Figure S35**. HRMS spectrum of compound **72**

**
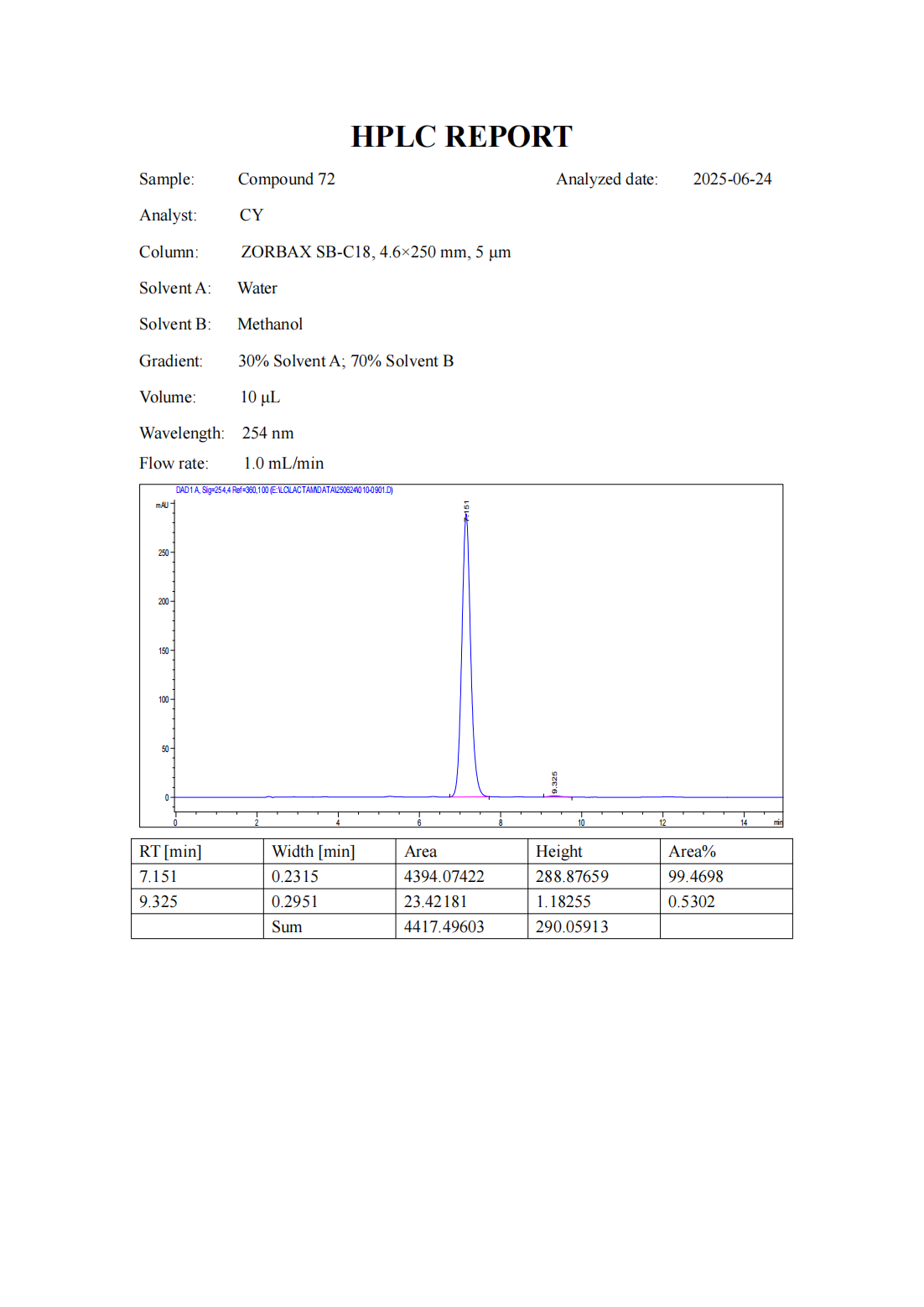
**

**Figure S36**. Chromatogram of compound **72**


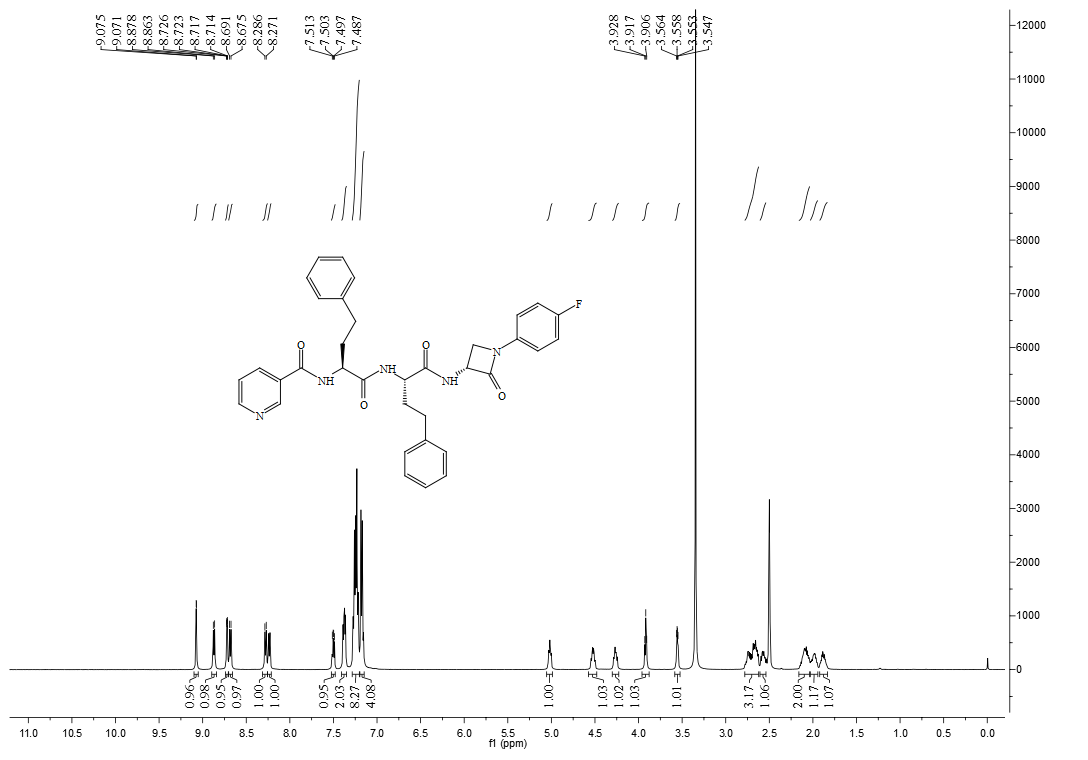


**Figure S37**. ^1^H NMR spectrum of compound **73** in DMSO-*d*_6_


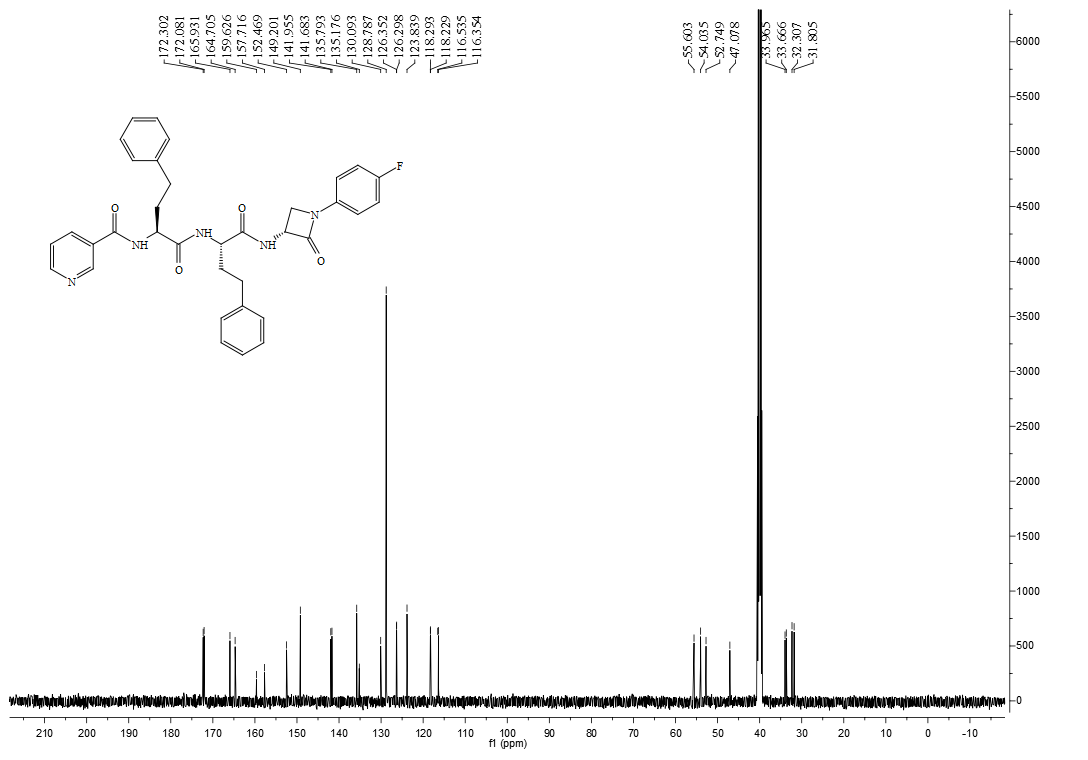


**Figure S38**. ^13^C NMR spectrum of compound **73** in DMSO-*d*_6_


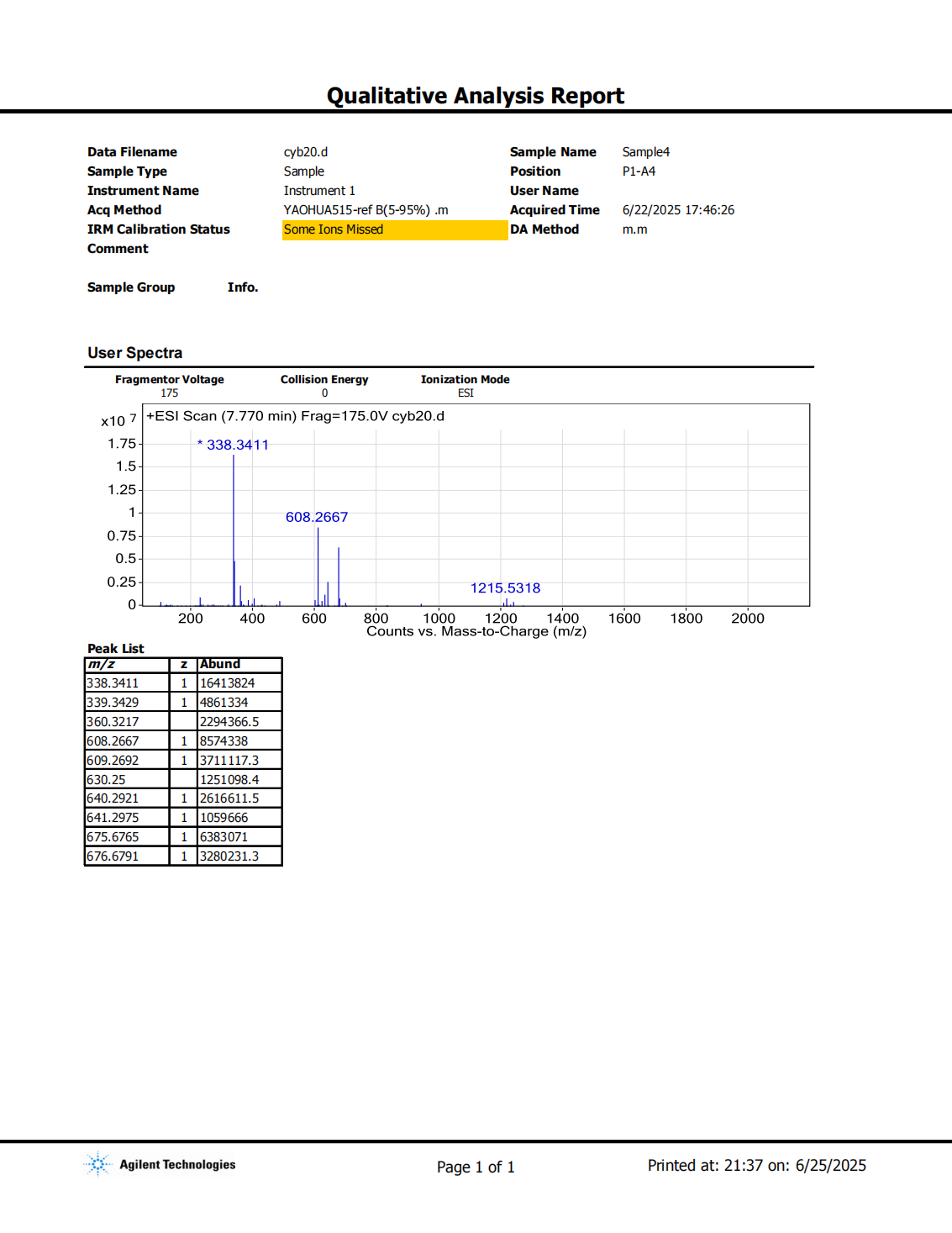


**Figure S39**. HRMS spectrum of compound **73**

**
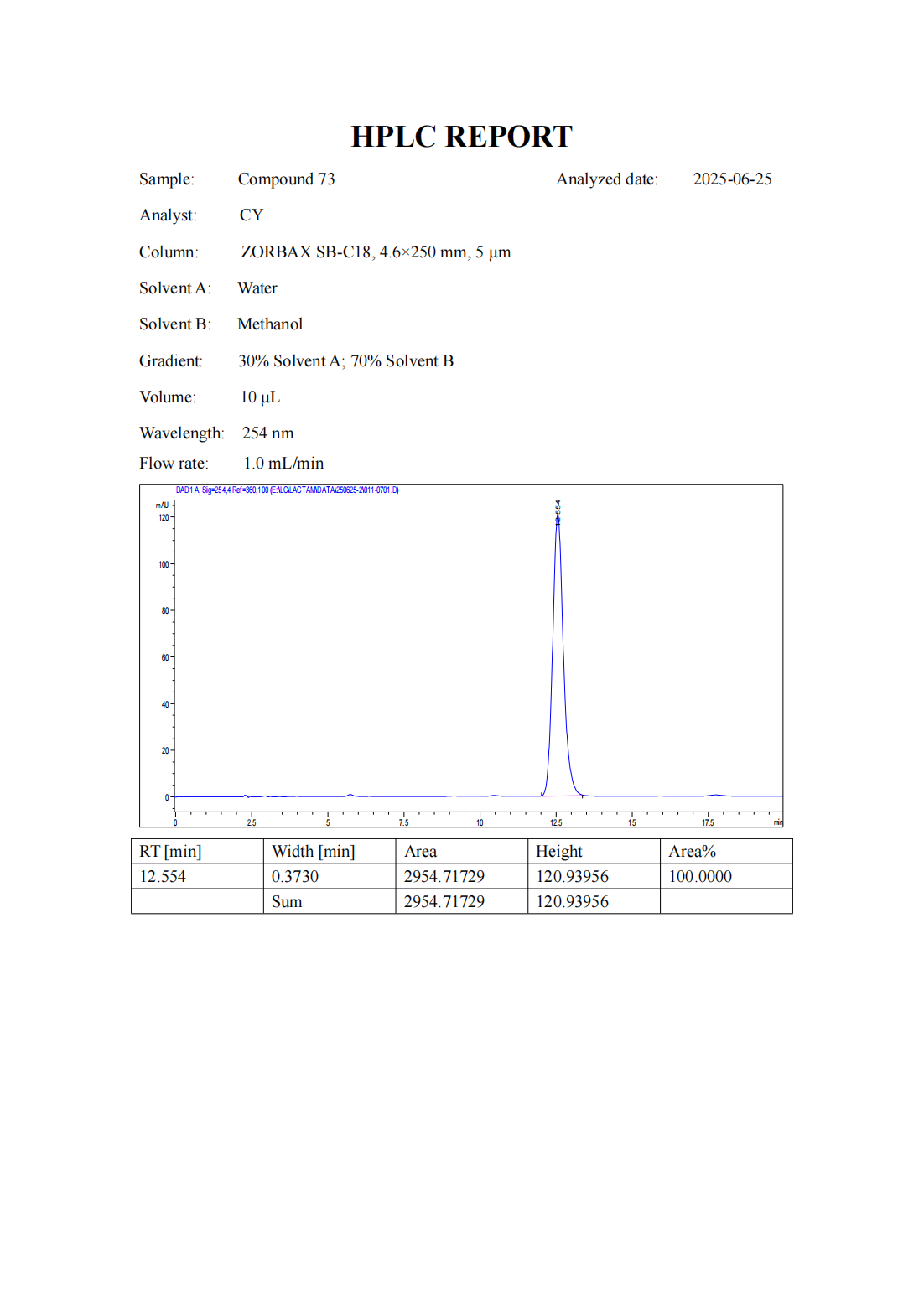
**

**Figure S40**. Chromatogram of compound **73**


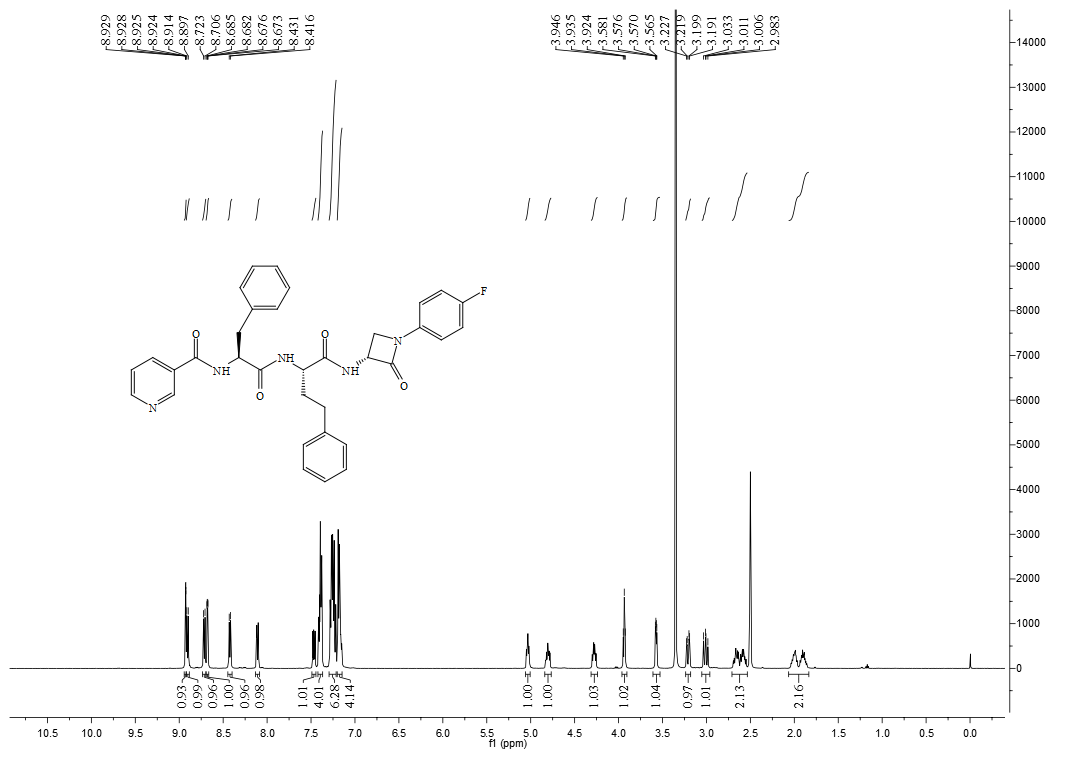


**Figure S41**. ^1^H NMR spectrum of compound **74** in DMSO-*d*_6_


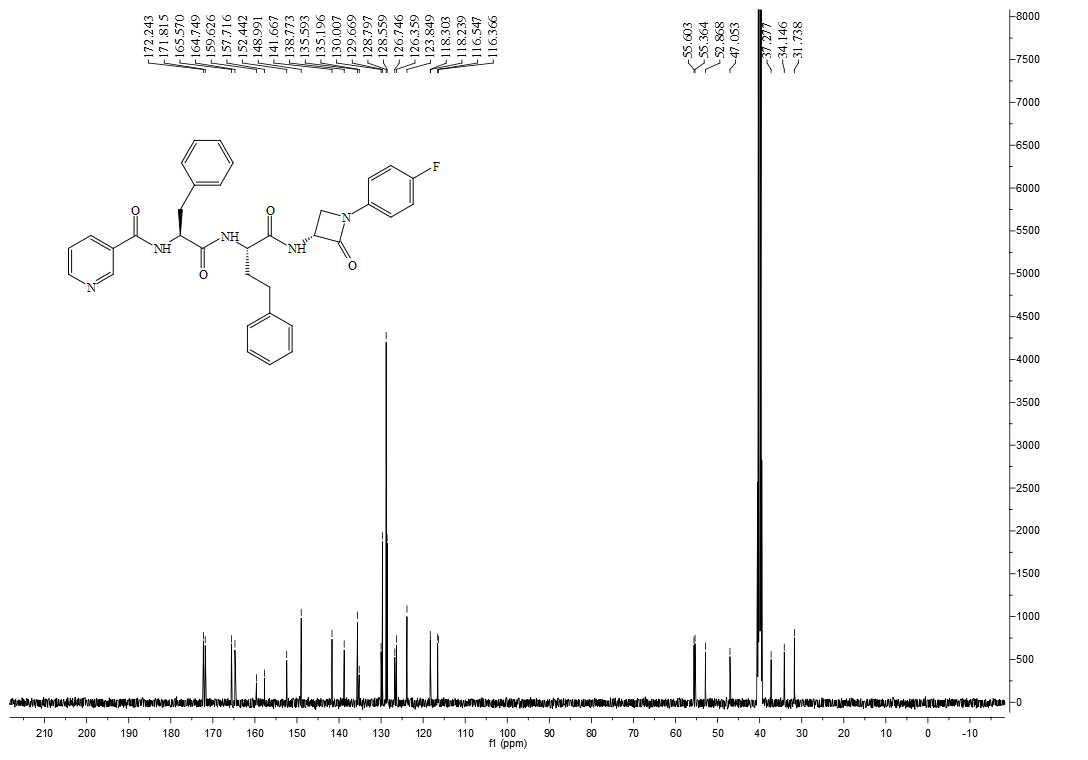


**Figure S42**. ^1^H NMR spectrum of compound **74** in DMSO-*d*_6_


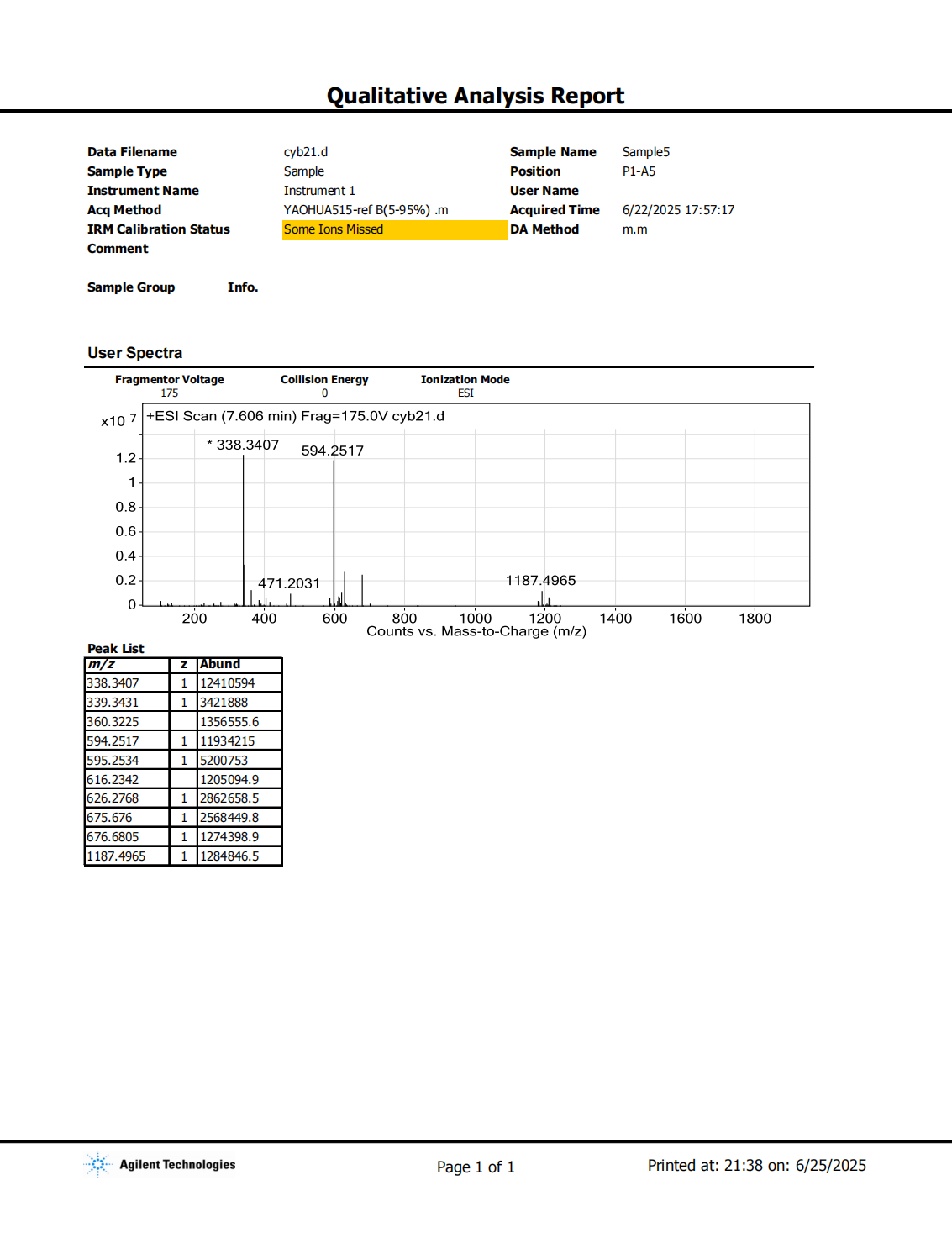


**Figure S43**. HRMS spectrum of compound **74**

**
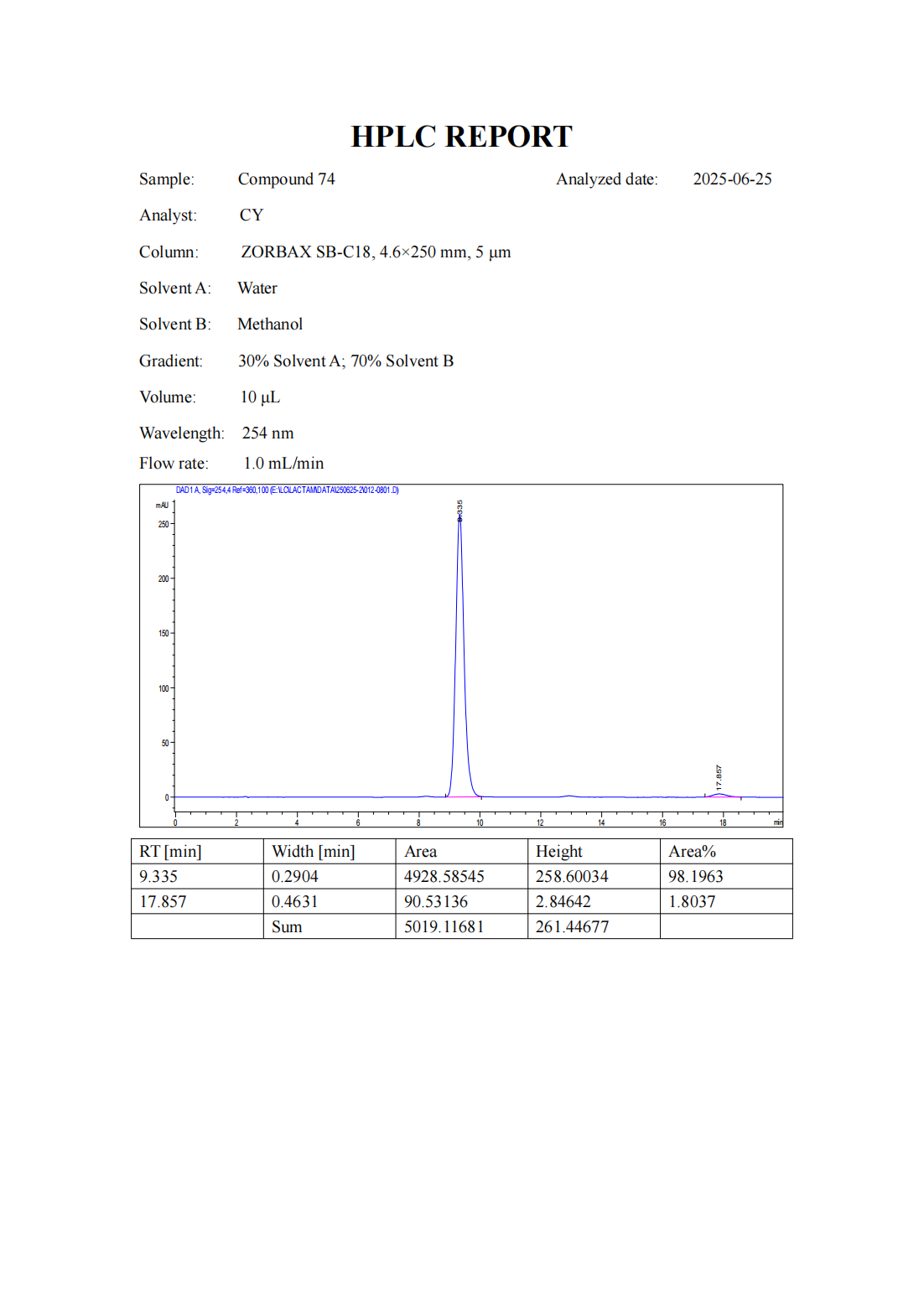
**

**Figure S44**. Chromatogram of compound **74**


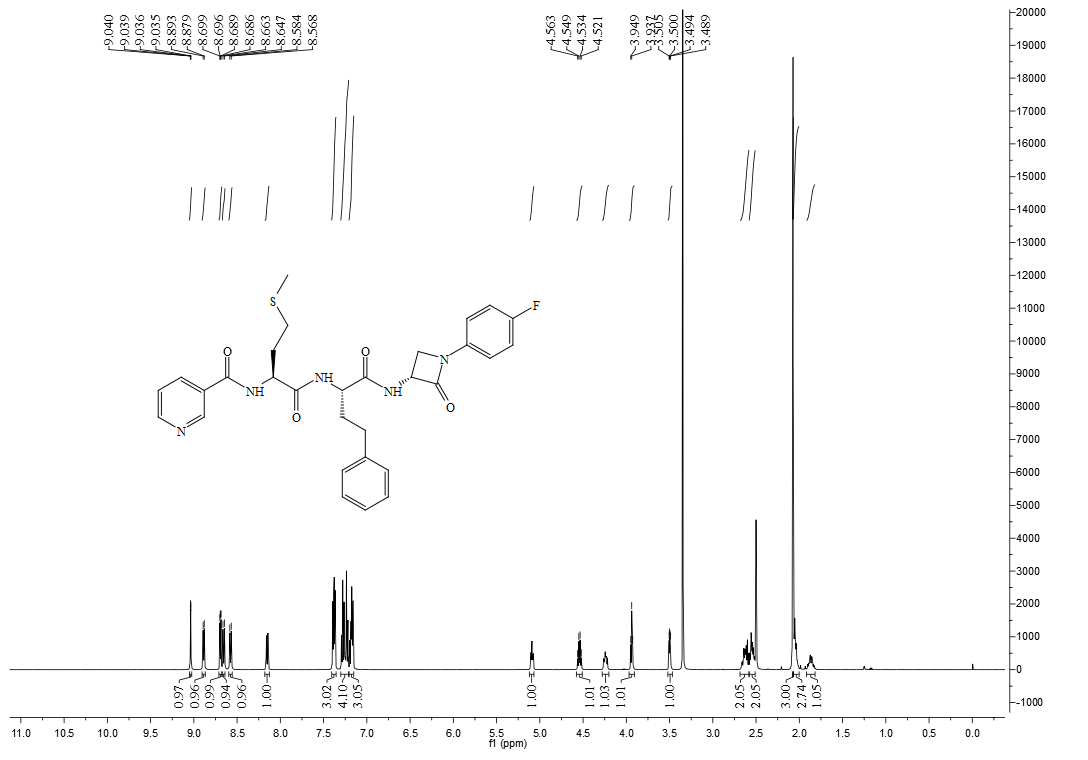


**Figure S45**. ^1^H NMR spectrum of compound **75** in DMSO-*d*_6_


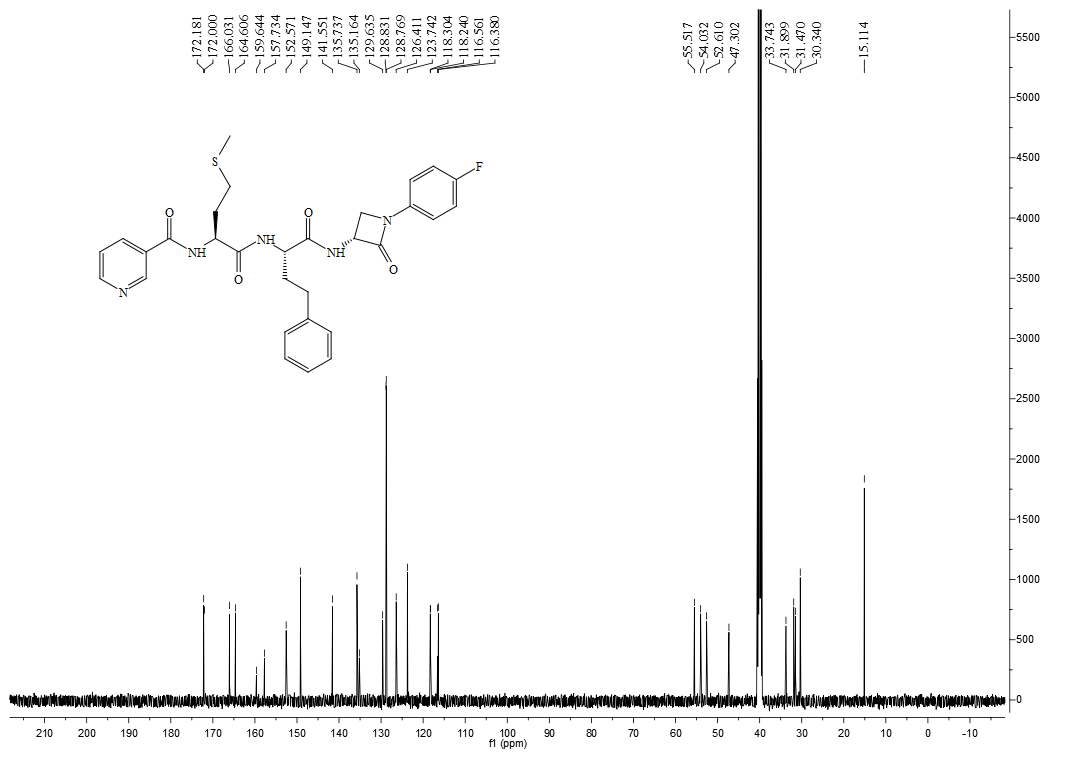


**Figure S46**. ^13^C NMR spectrum of compound **75** in DMSO-*d*_6_


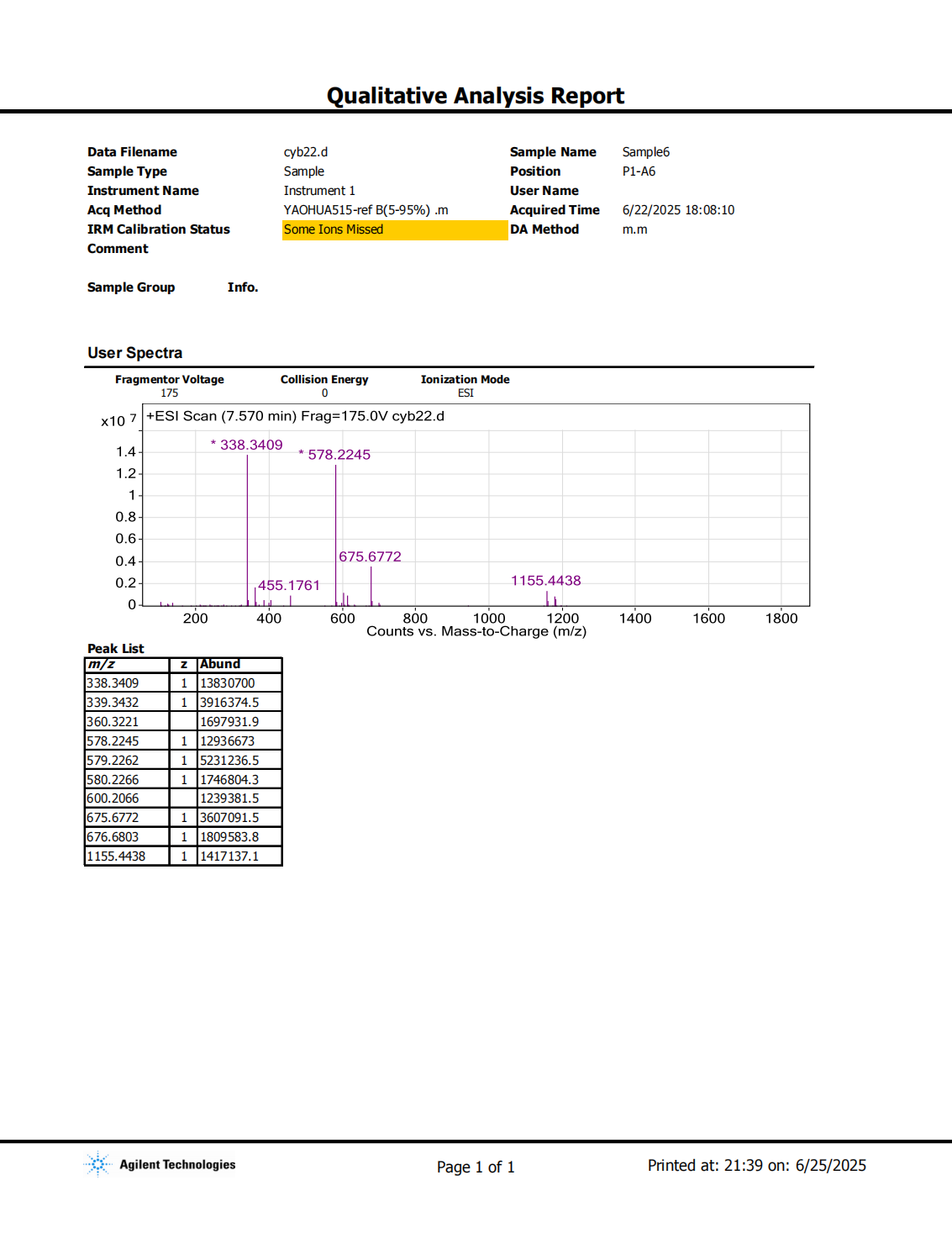


**Figure S47**. HRMS spectrum of compound **75**


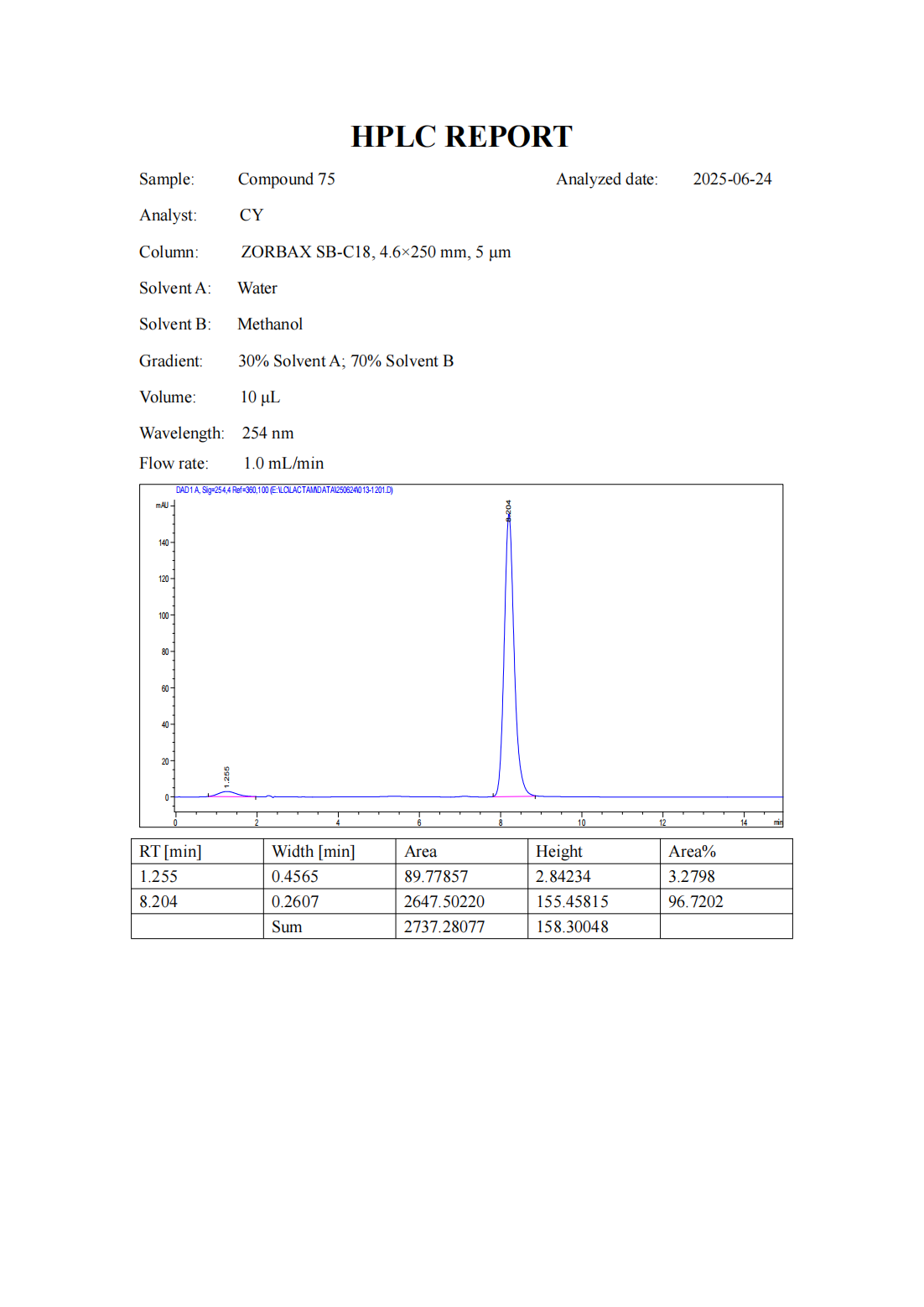


**Figure S48**. Chromatogram of compound **75**


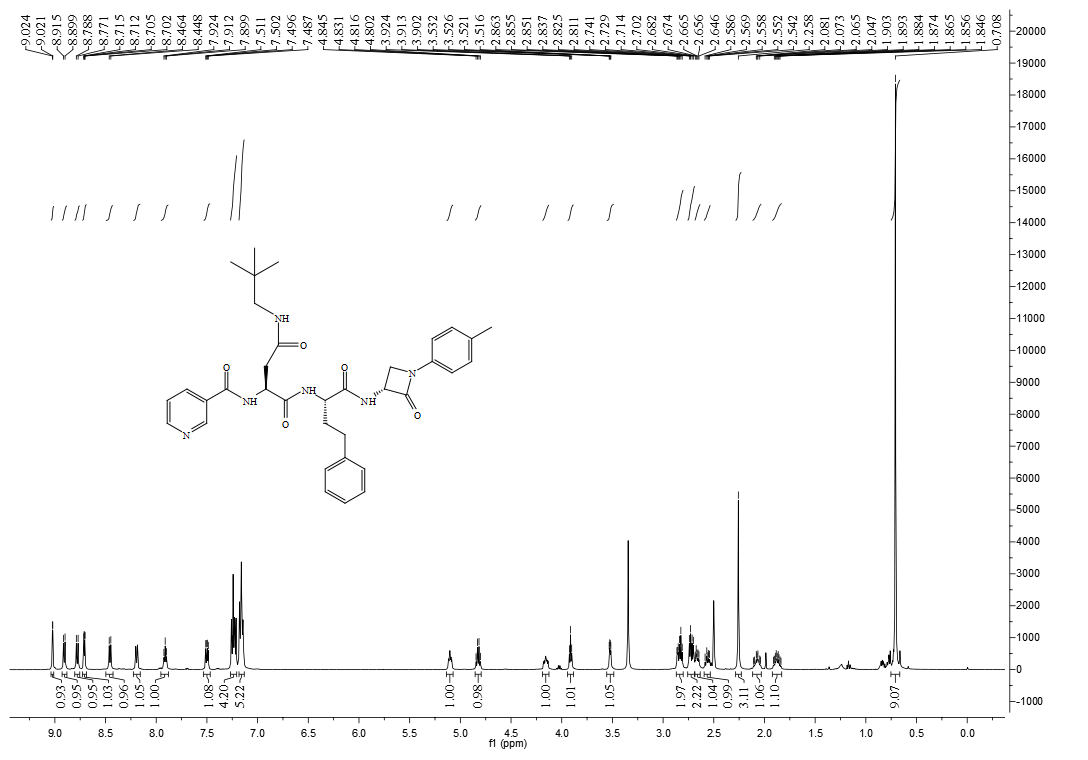


**Figure S49**. ^1^H NMR spectrum of compound **76** in DMSO-*d*_6_


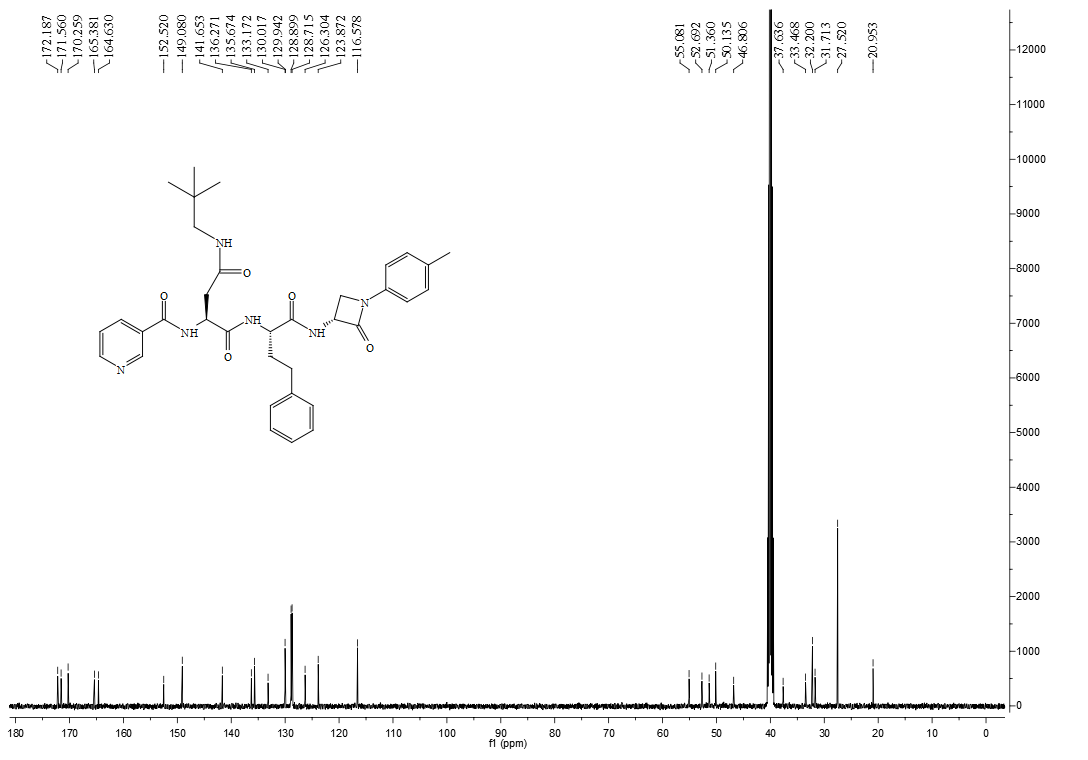


**Figure S50**. ^13^C NMR spectrum of compound **76** in DMSO-*d*_6_

**
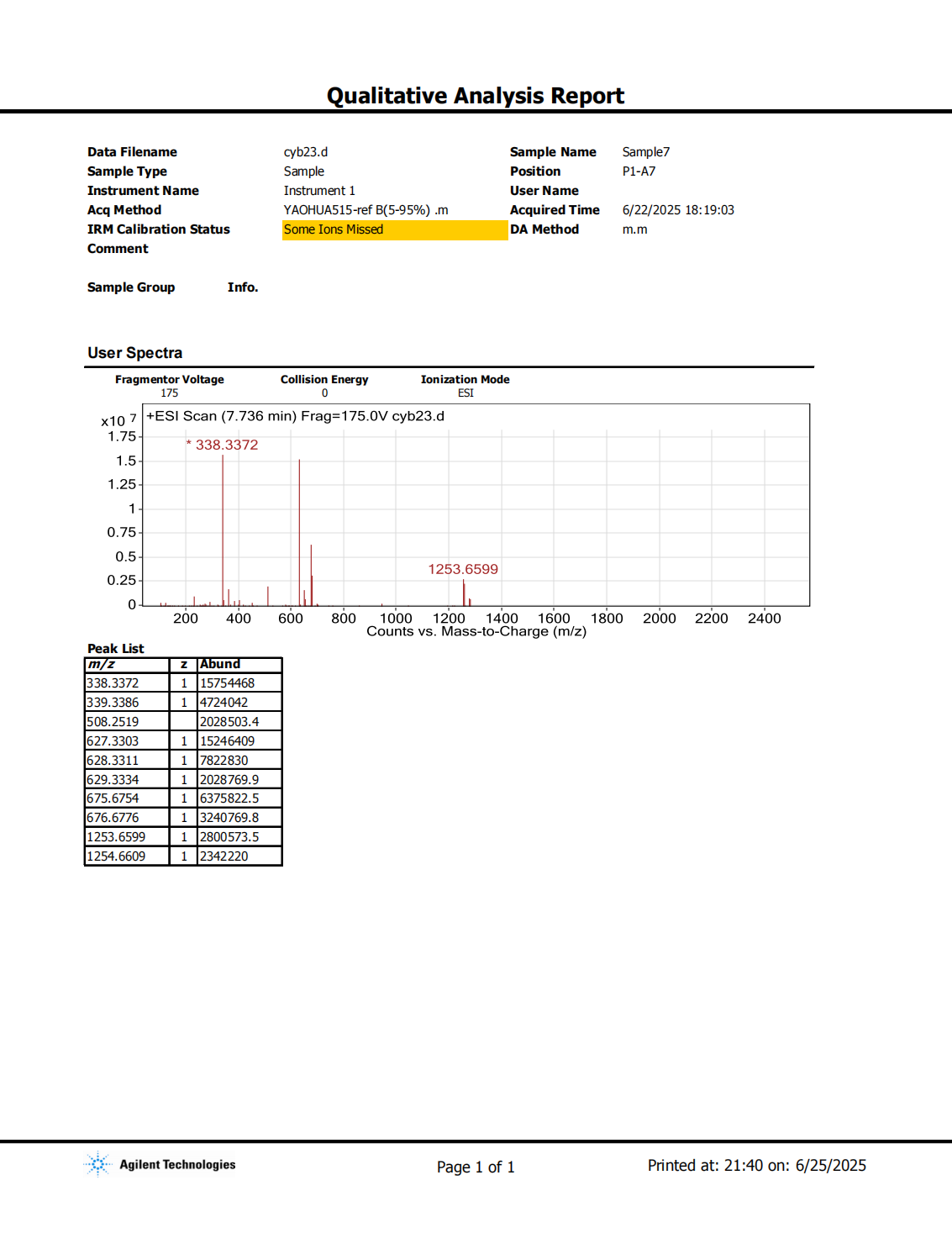
**

**Figure S51**. HRMS spectrum of compound **76**

**
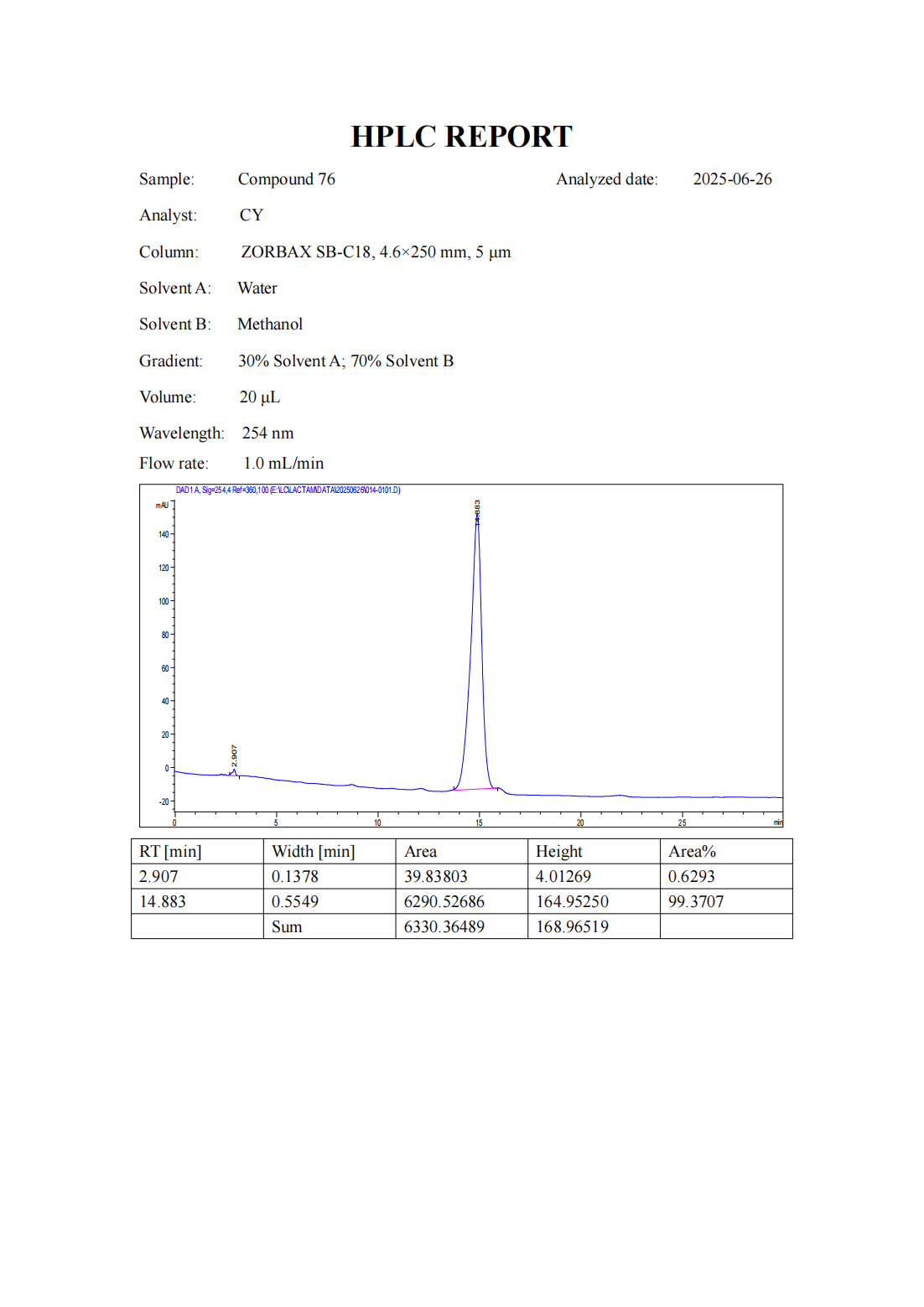
**

**Figure S52**. Chromatogram of compound **76**


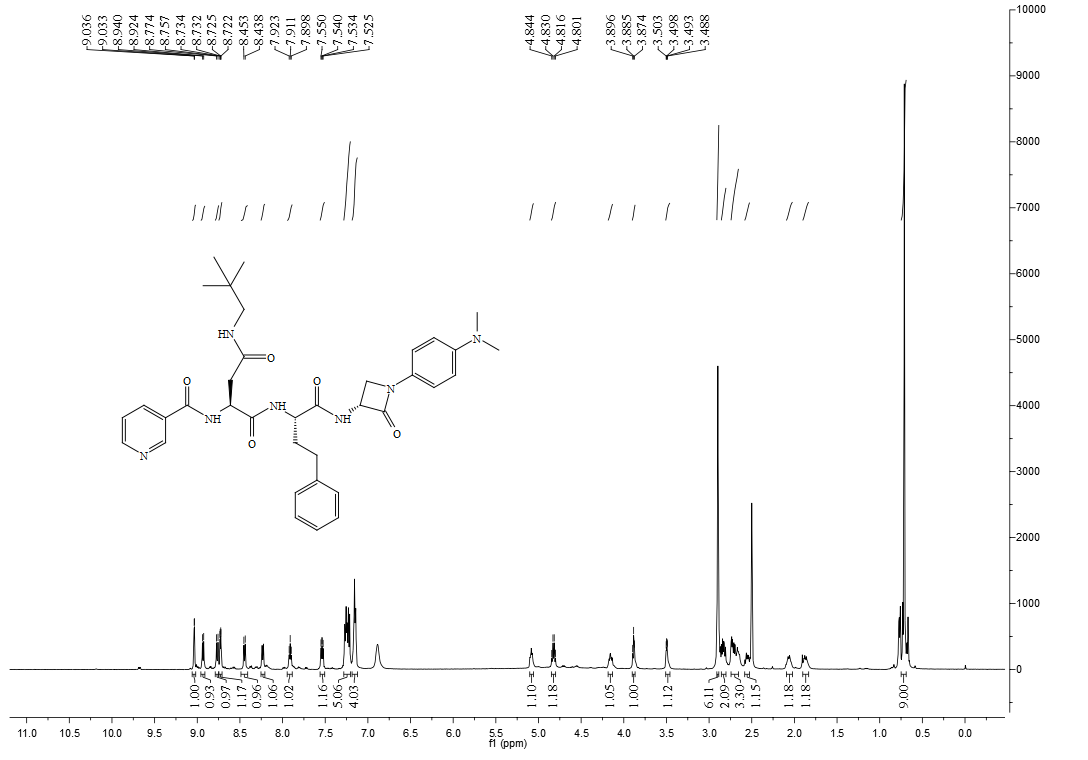


**Figure S53**. ^1^H NMR spectrum of compound **77** in DMSO-*d*_6_


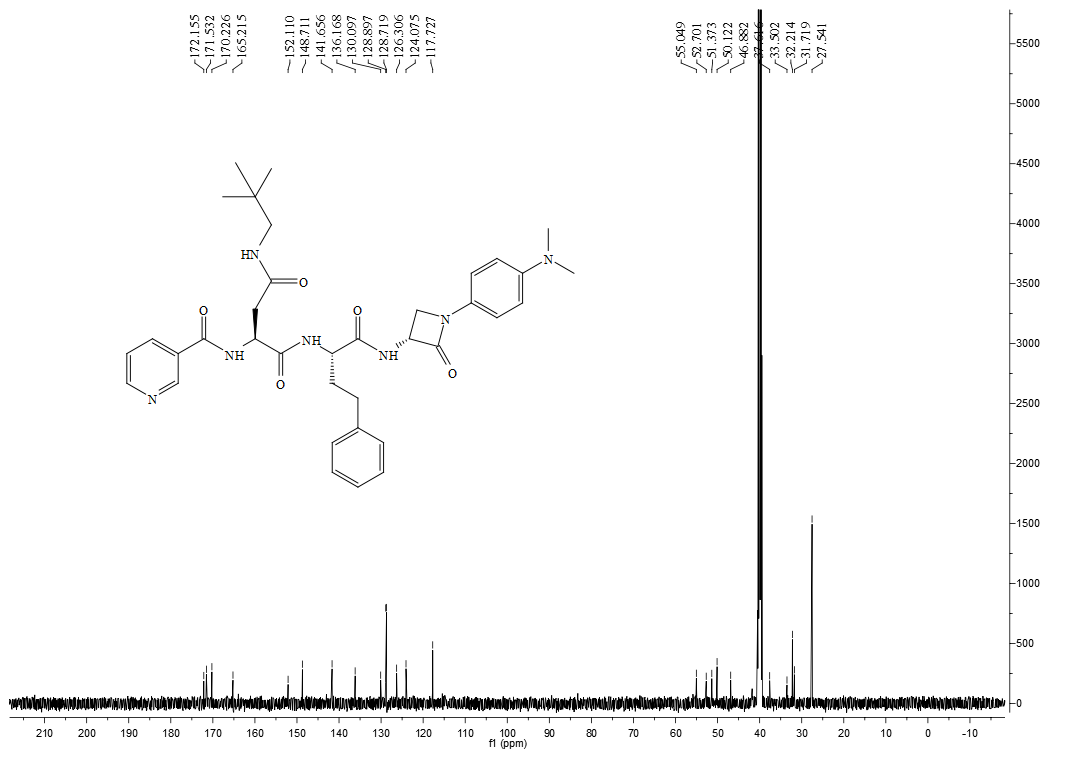


**Figure S54**. ^13^C NMR spectrum of compound **77** in DMSO-*d*_6_


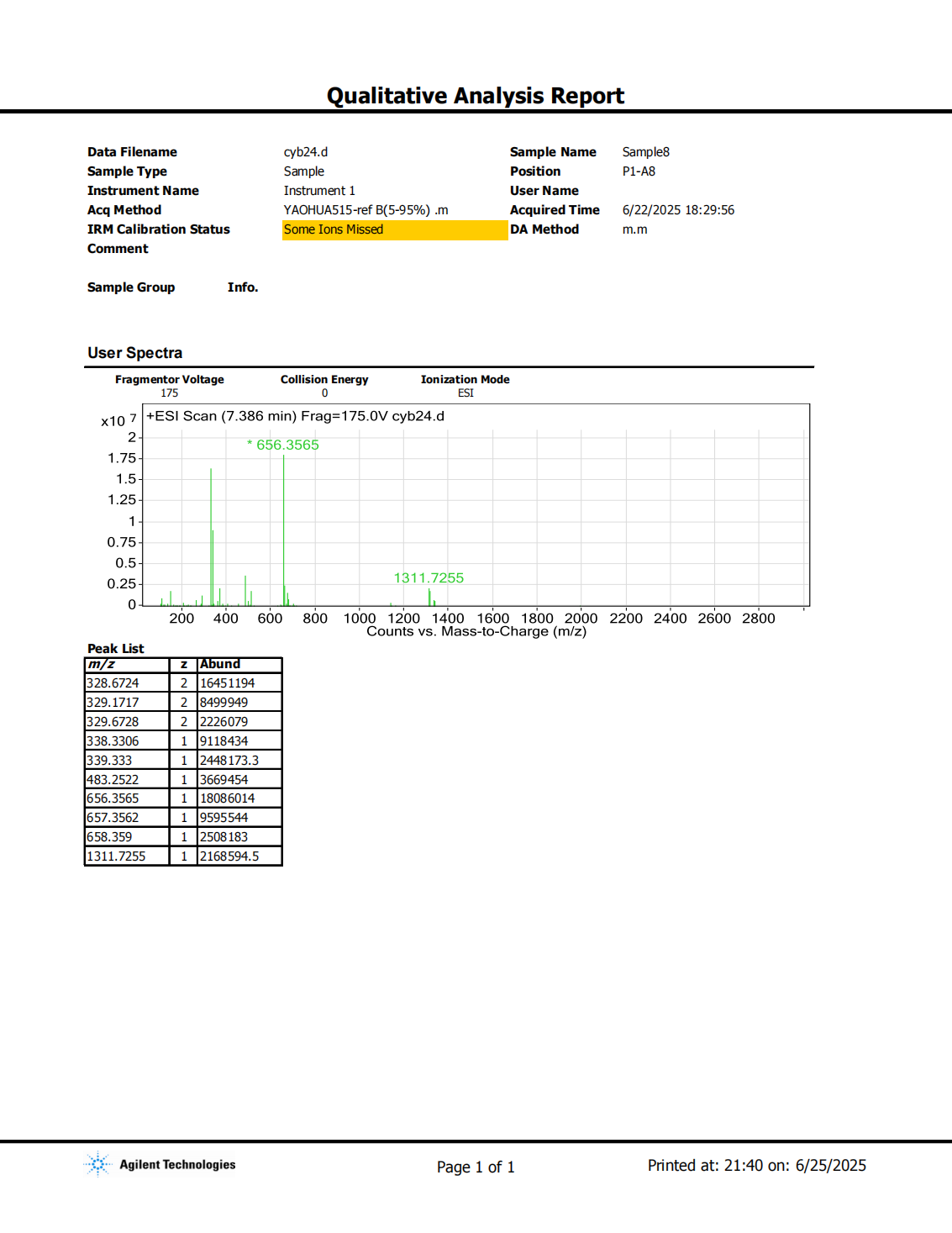


**Figure S55**. HRMS spectrum of compound **77**


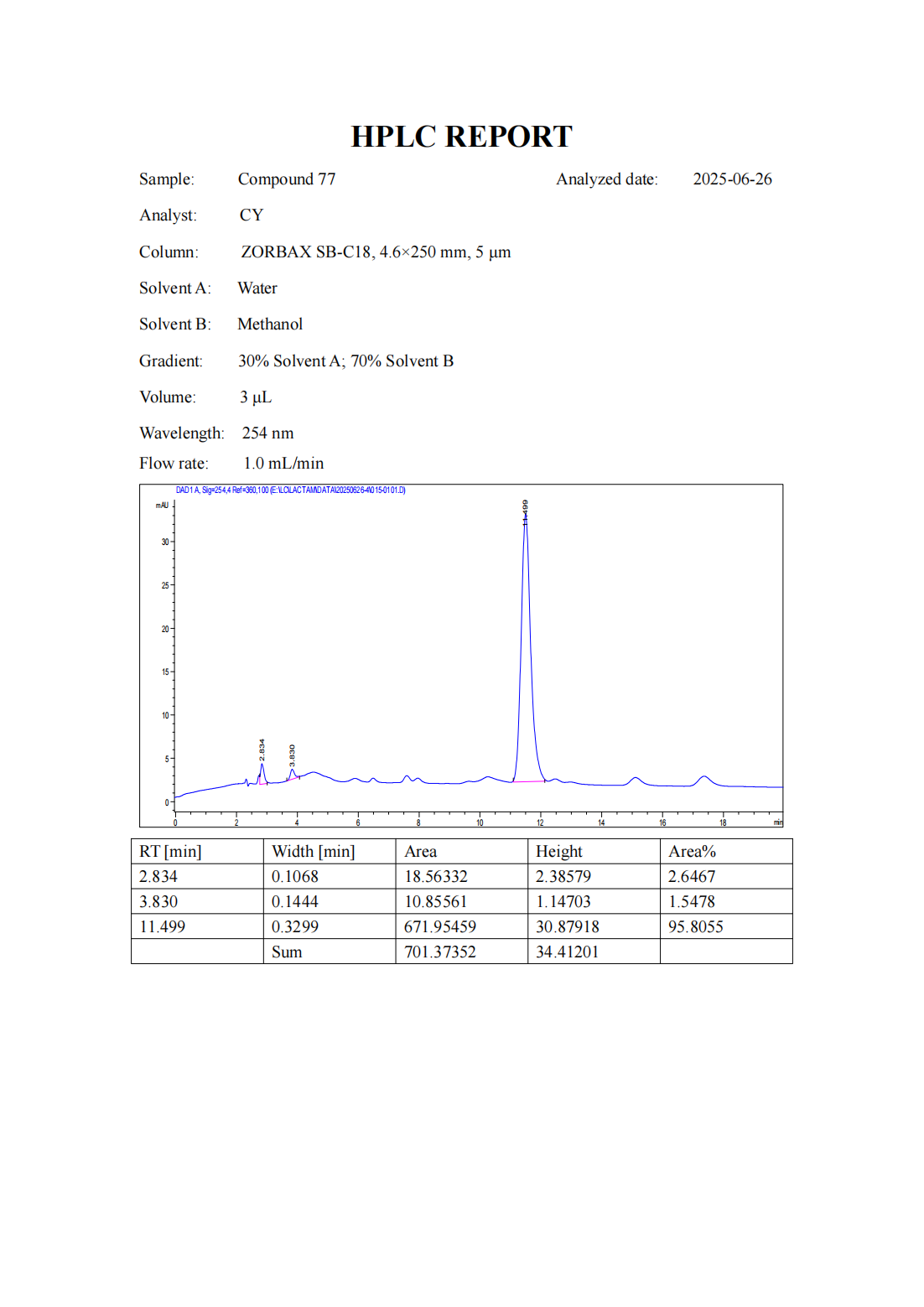


**Figure S56**. Chromatogram of compound **77**


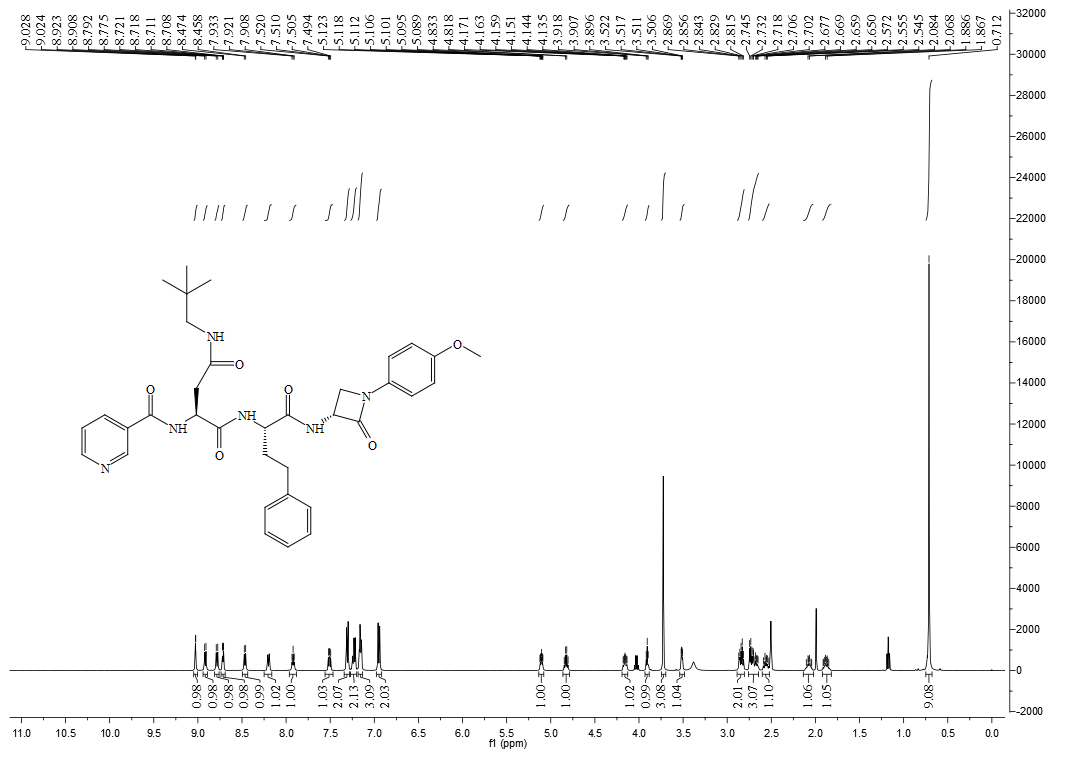


**Figure S57**. ^1^H NMR spectrum of compound **78** in DMSO-*d*_6_


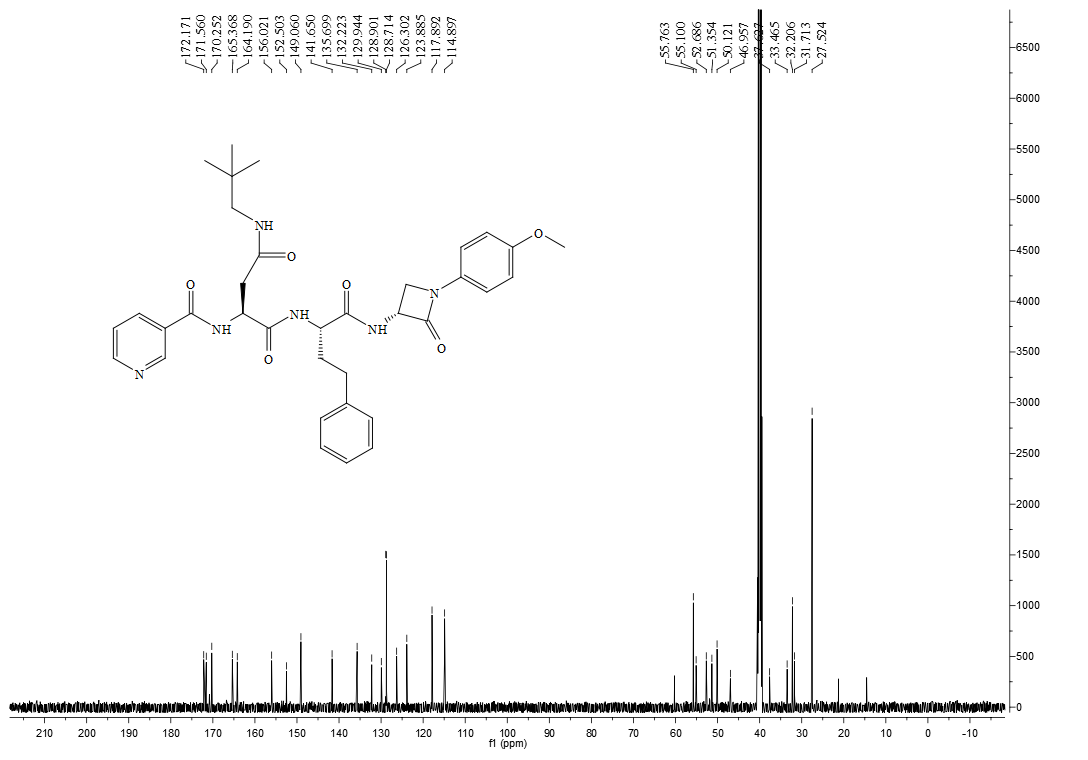


**Figure S58**. ^1^H NMR spectrum of compound **78** in DMSO-*d*_6_

_
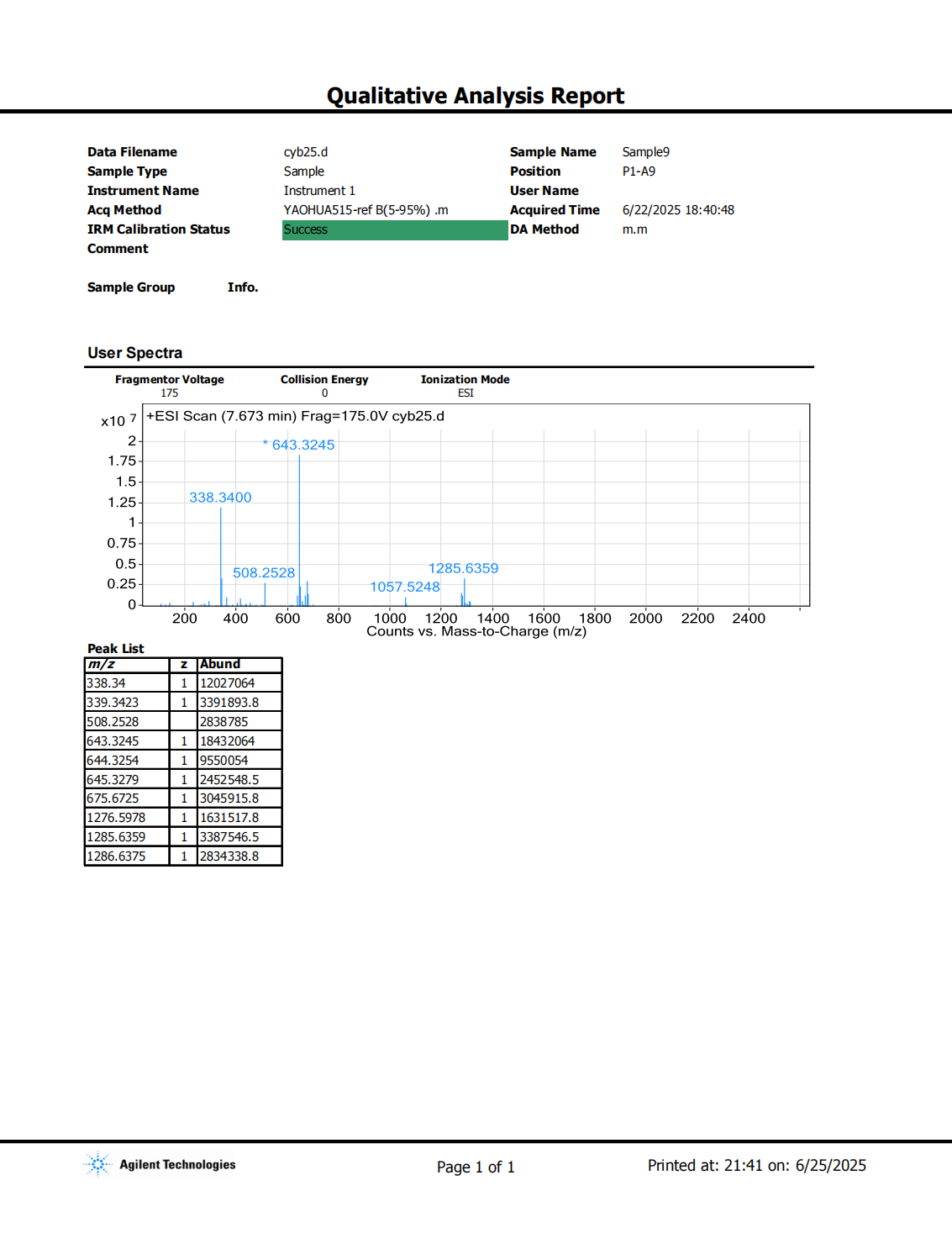
_

**Figure S59**. HRMS spectrum of compound **78**

**
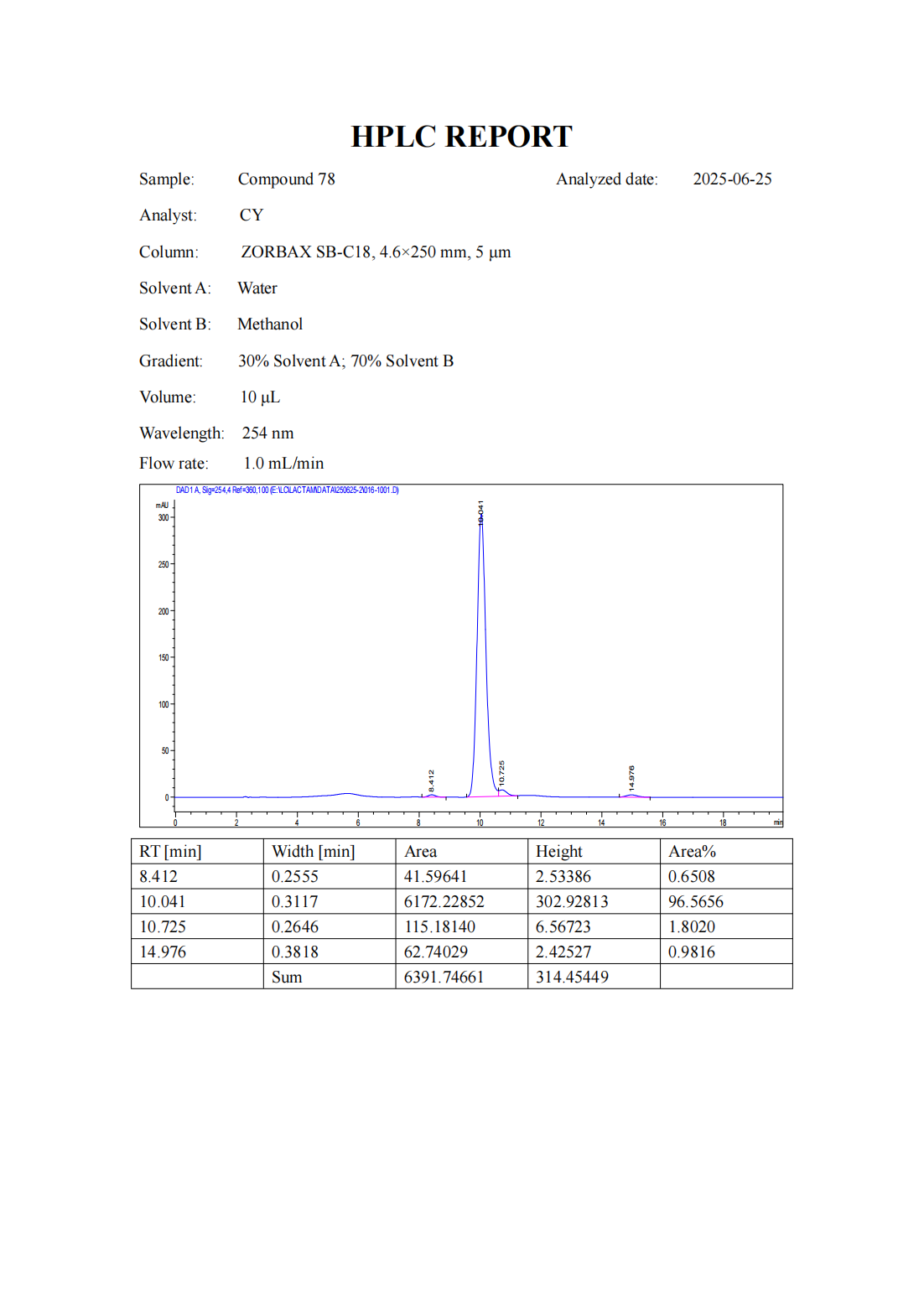
**

**Figure S60**. Chromatogram of compound **78**

**
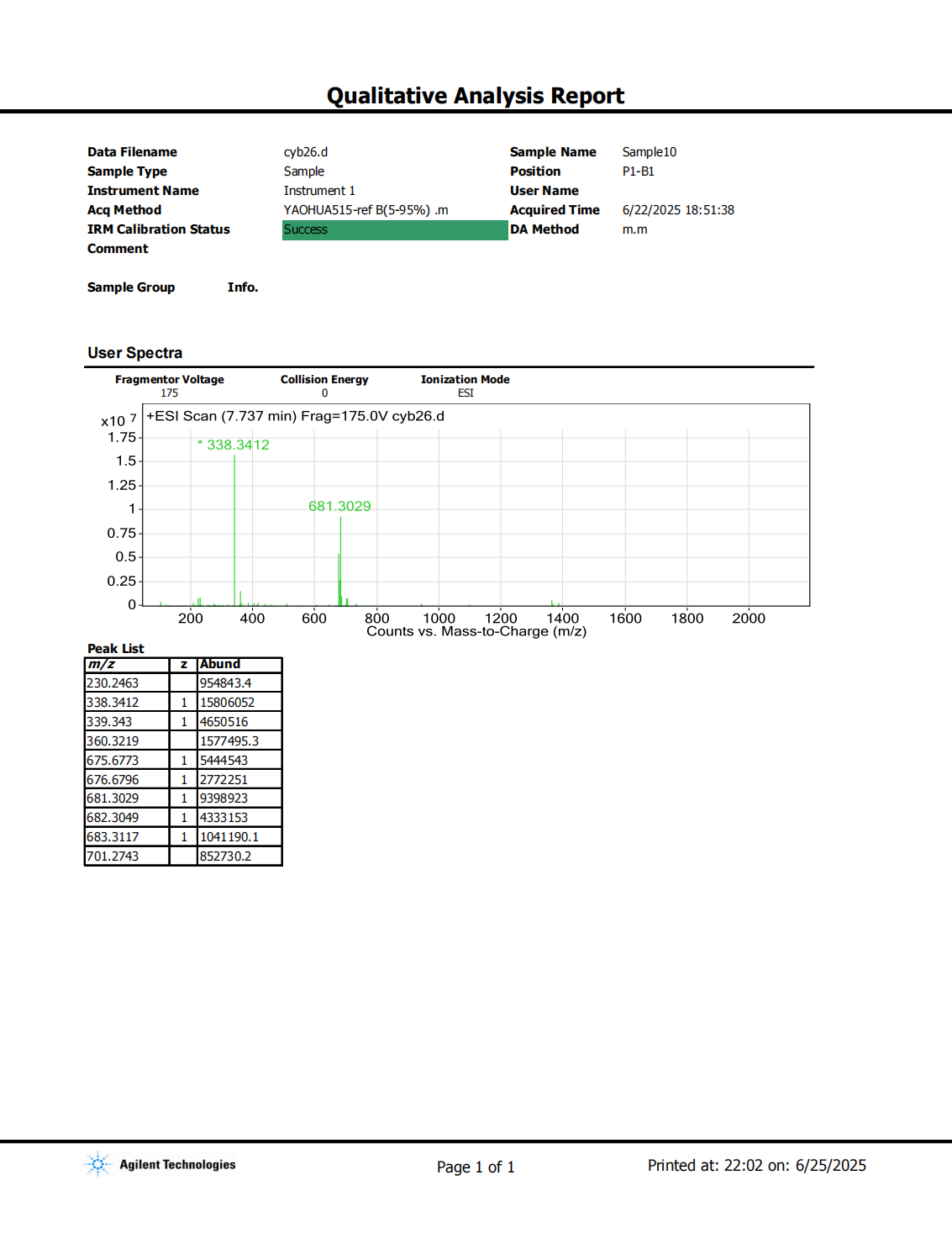
**

**Figure S61**. HRMS spectrum of compound **79**

**
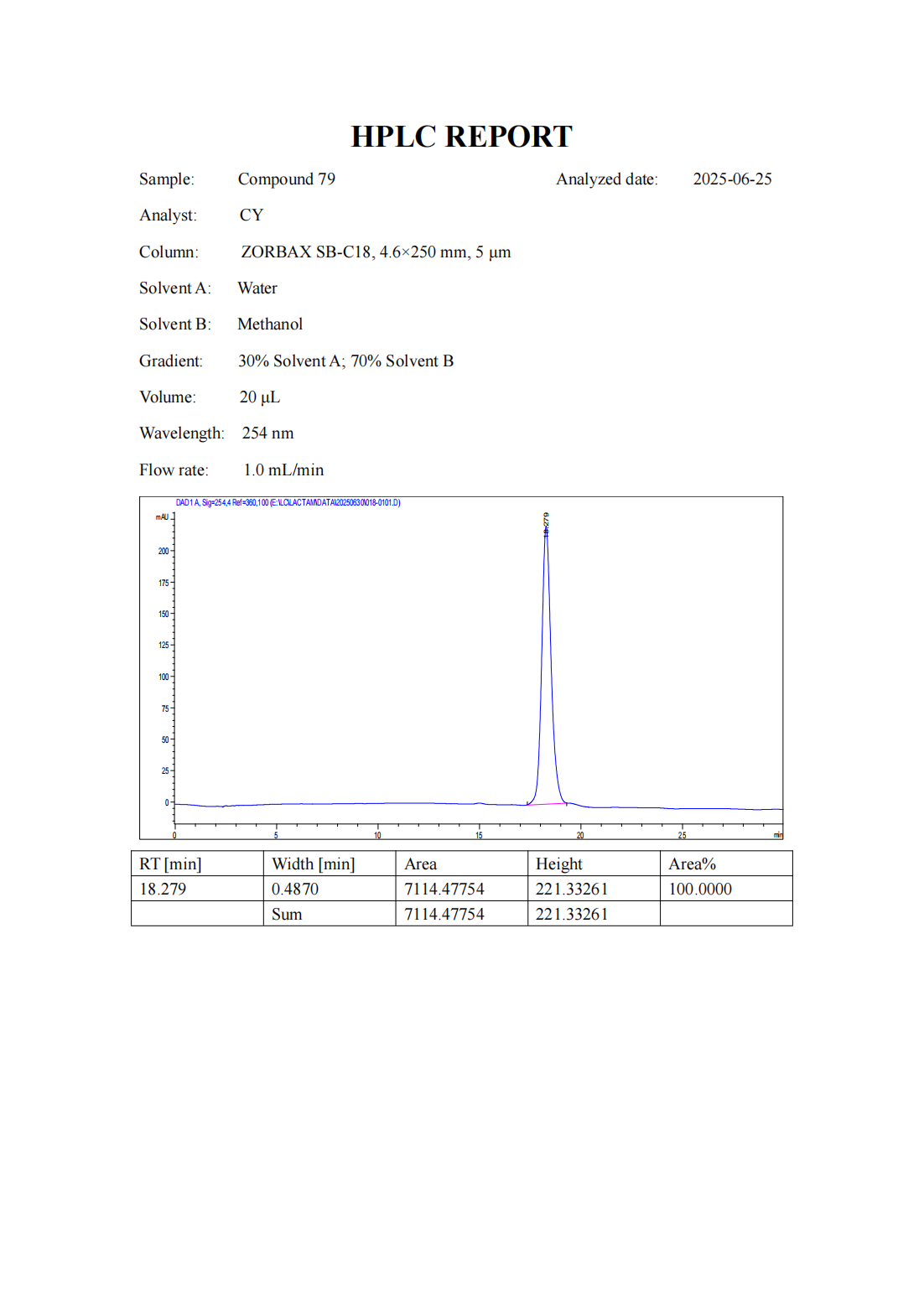
**

**Figure S62**. Chromatogram of compound **79**


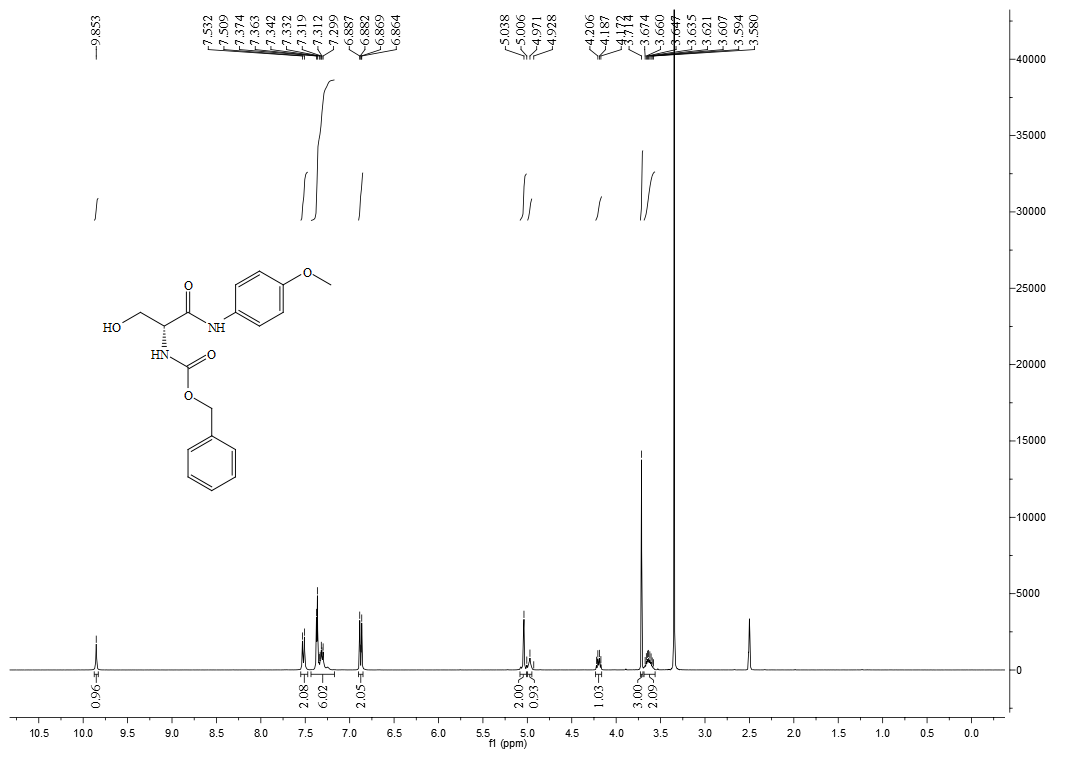


**Figure S63**. ^1^H NMR spectrum of compound **7** in DMSO-*d*_6_


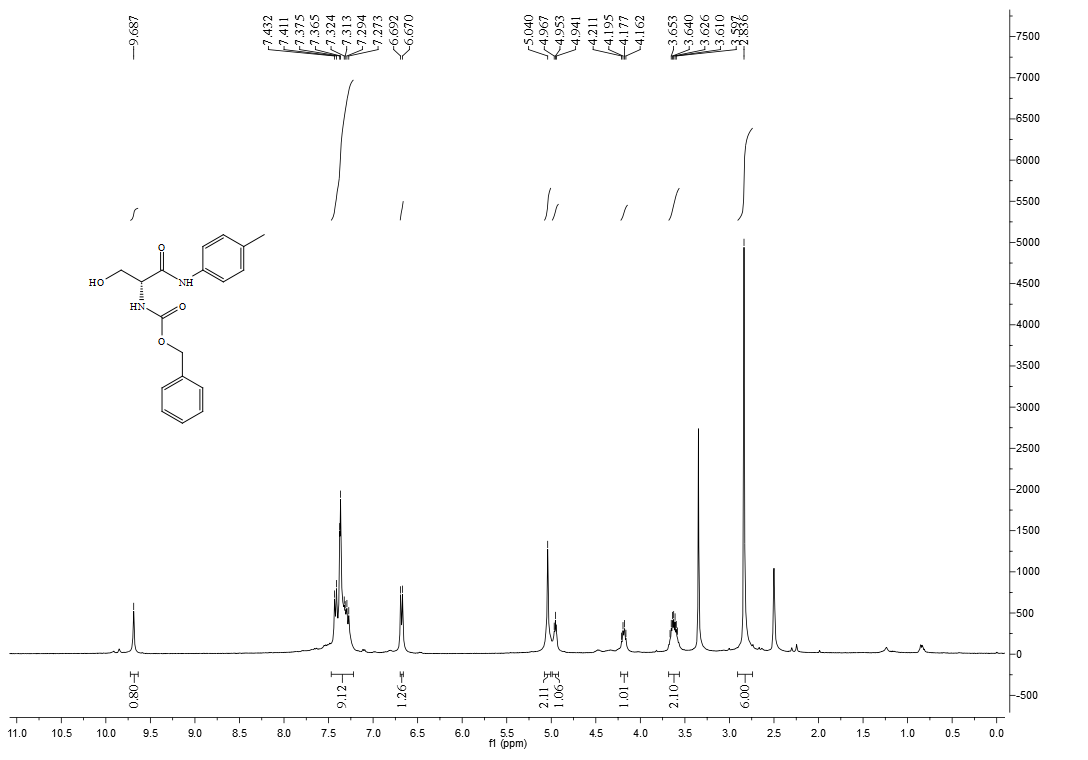


**Figure S64**. ^1^H NMR spectrum of compound **8** in DMSO-*d*_6_


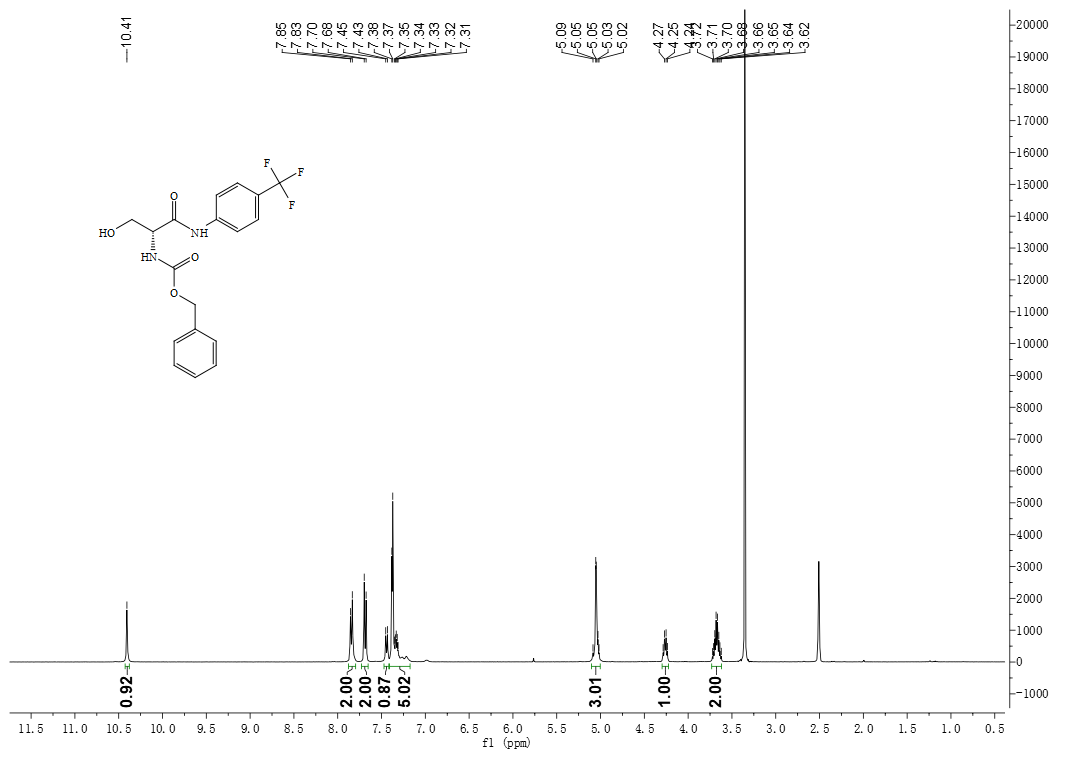


**Figure S65**. ^1^H NMR spectrum of compound **9** in DMSO-*d*_6_


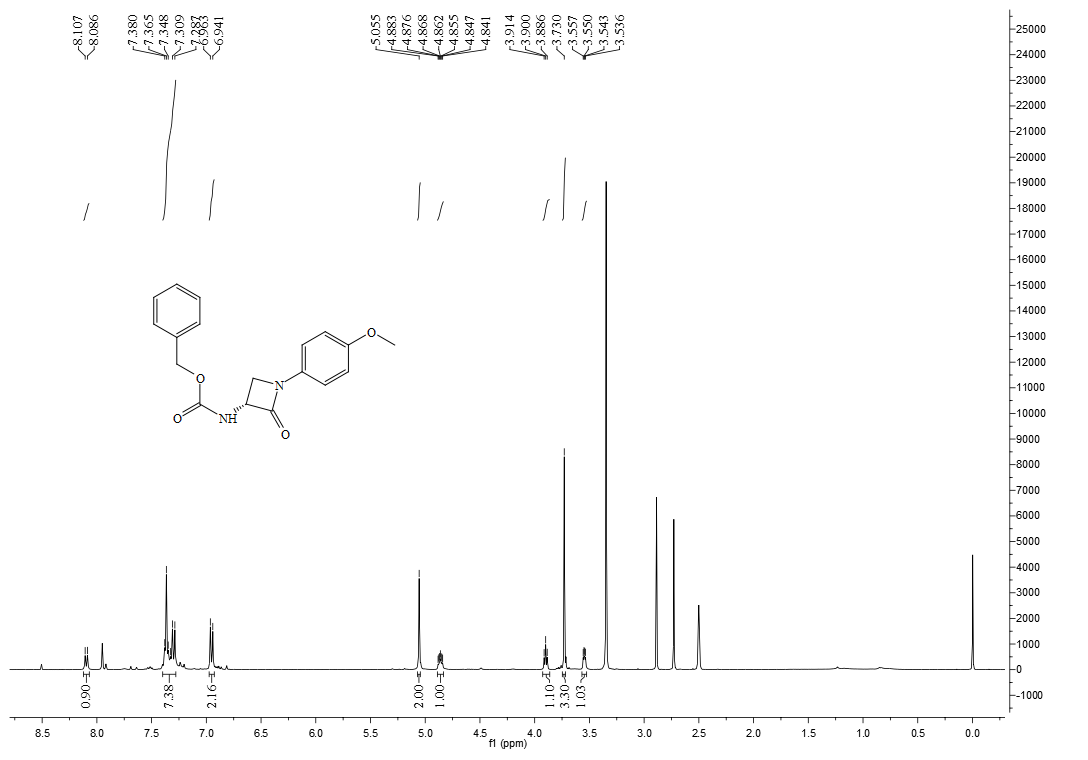


**Figure S66**. ^1^H NMR spectrum of compound **13** in DMSO-*d*_6_


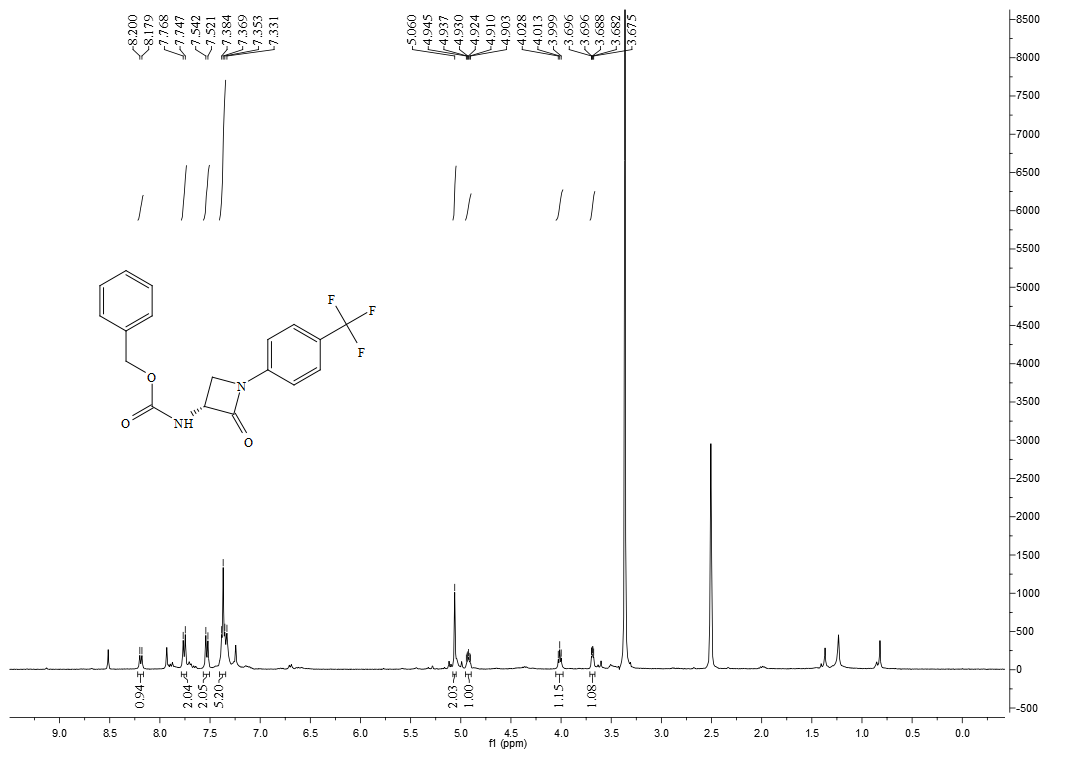


**Figure S67**. ^1^H NMR spectrum of compound **15** in DMSO-*d*_6_


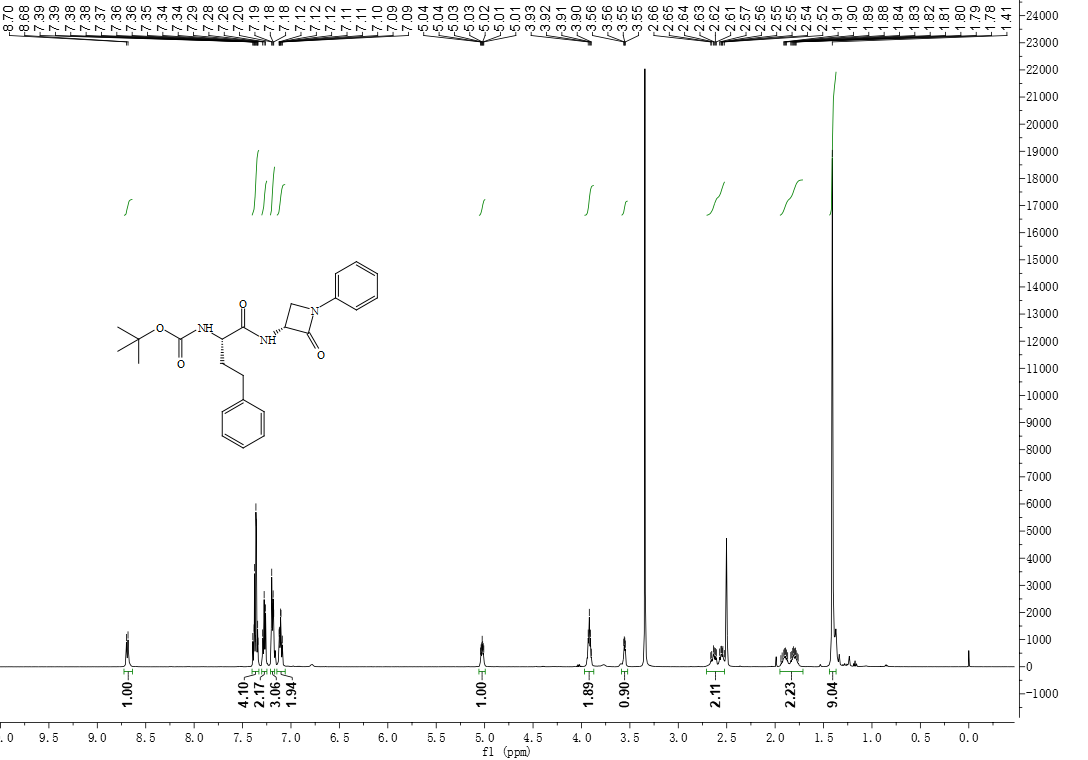


**Figure S68**. ^1^H NMR spectrum of compound **22** in DMSO-*d*_6_


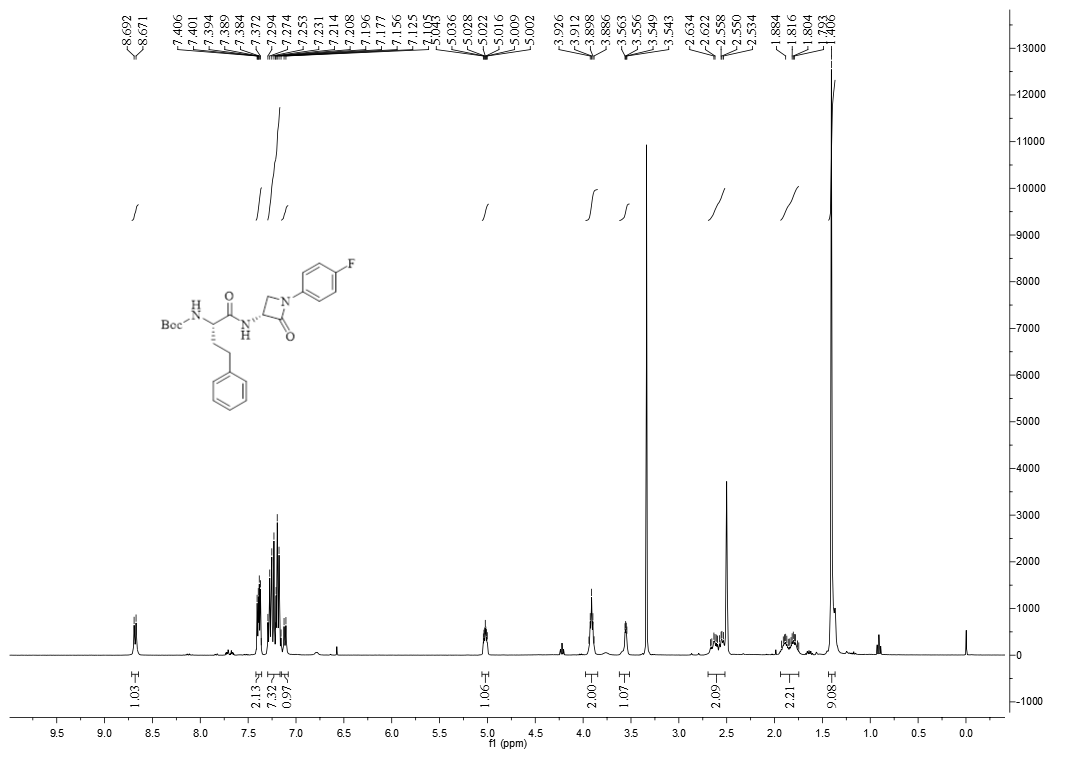


**Figure S69**. ^1^H NMR spectrum of compound **24** in DMSO-*d*_6_


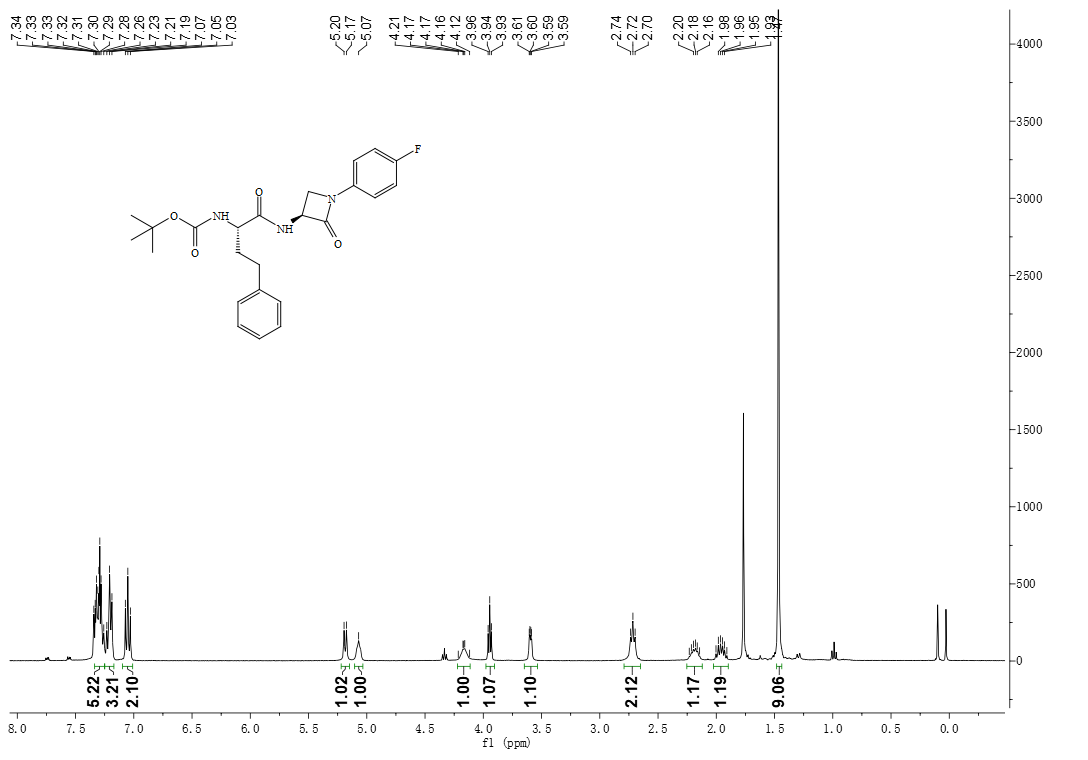


**Figure S70**. ^1^H NMR spectrum of compound **25** in CDCl_3_


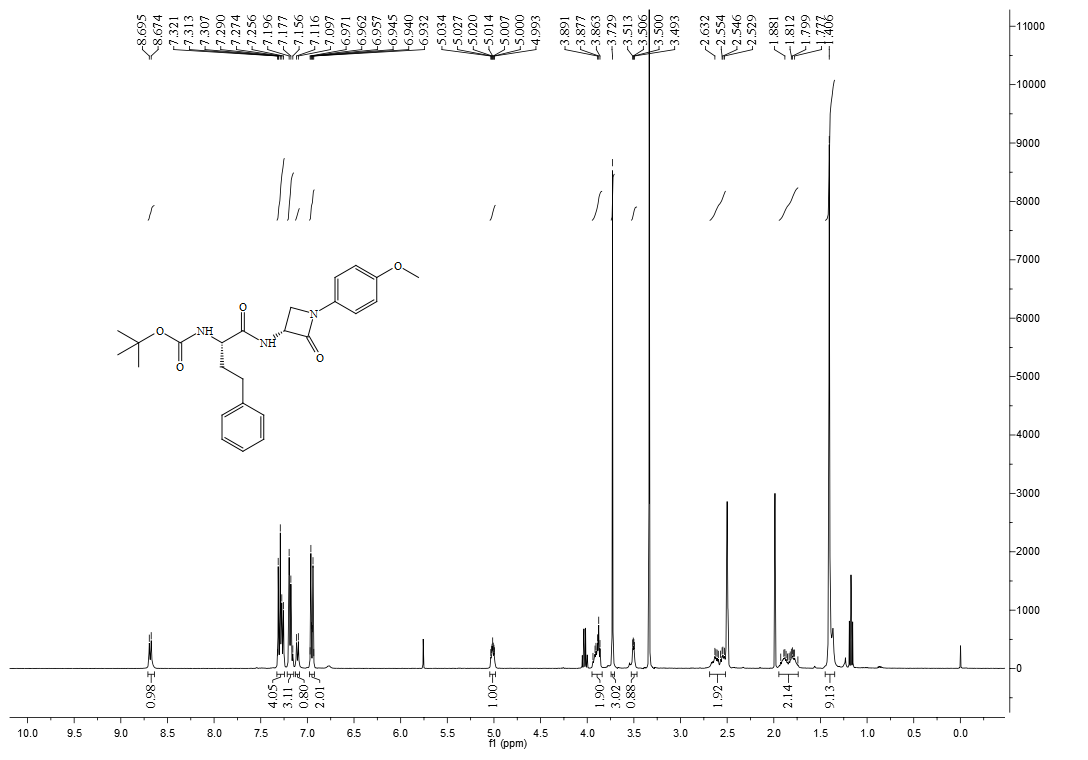


**Figure S71**. ^1^H NMR spectrum of compound **27** in DMSO-*d*_6_


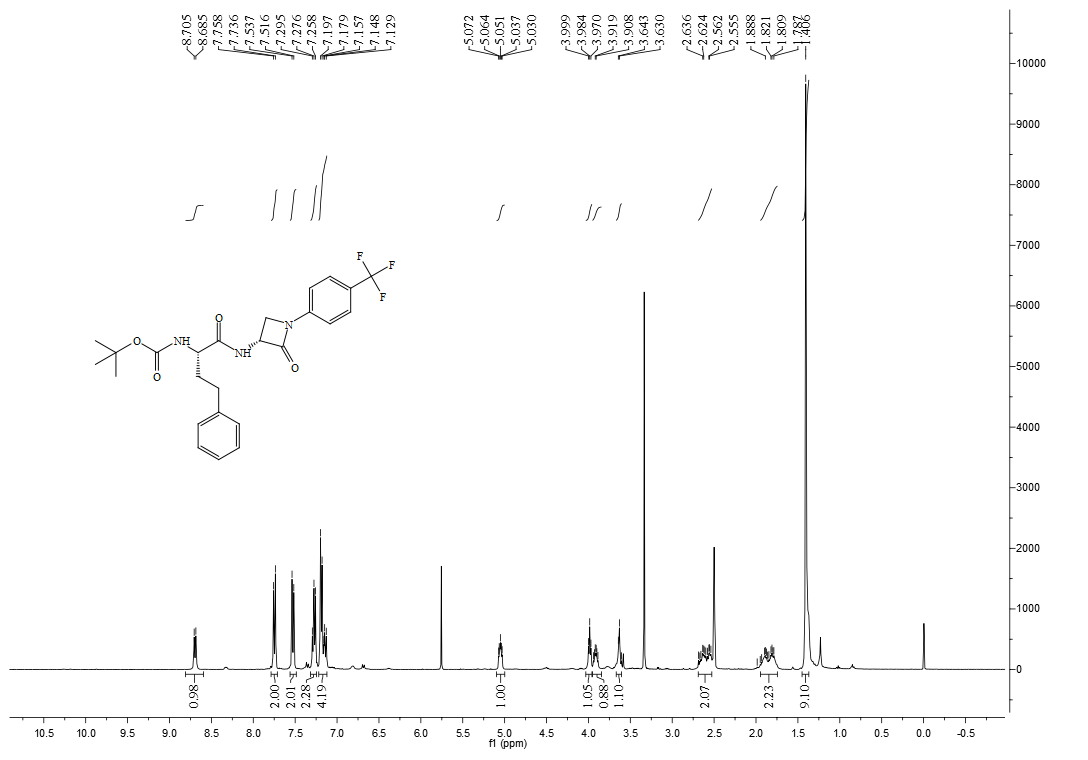


**Figure S72**. ^1^H NMR spectrum of compound **29** in DMSO-*d*_6_


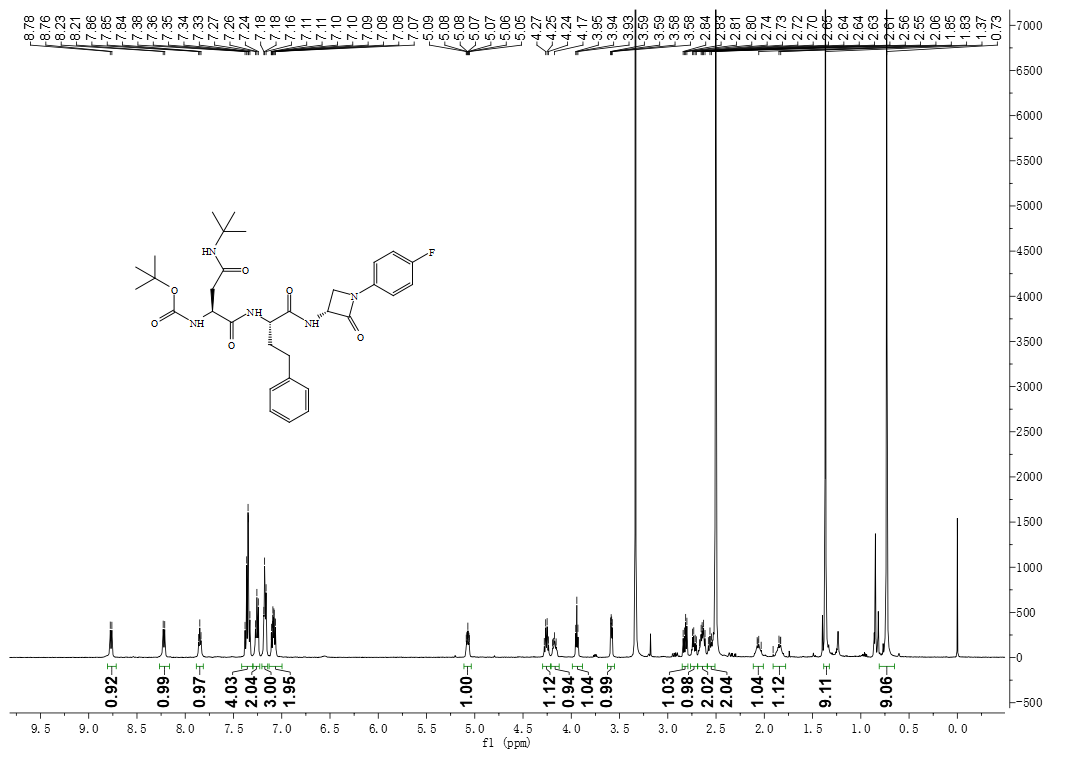


**Figure S73**. ^1^H NMR spectrum of compound **38** in DMSO-*d*_6_


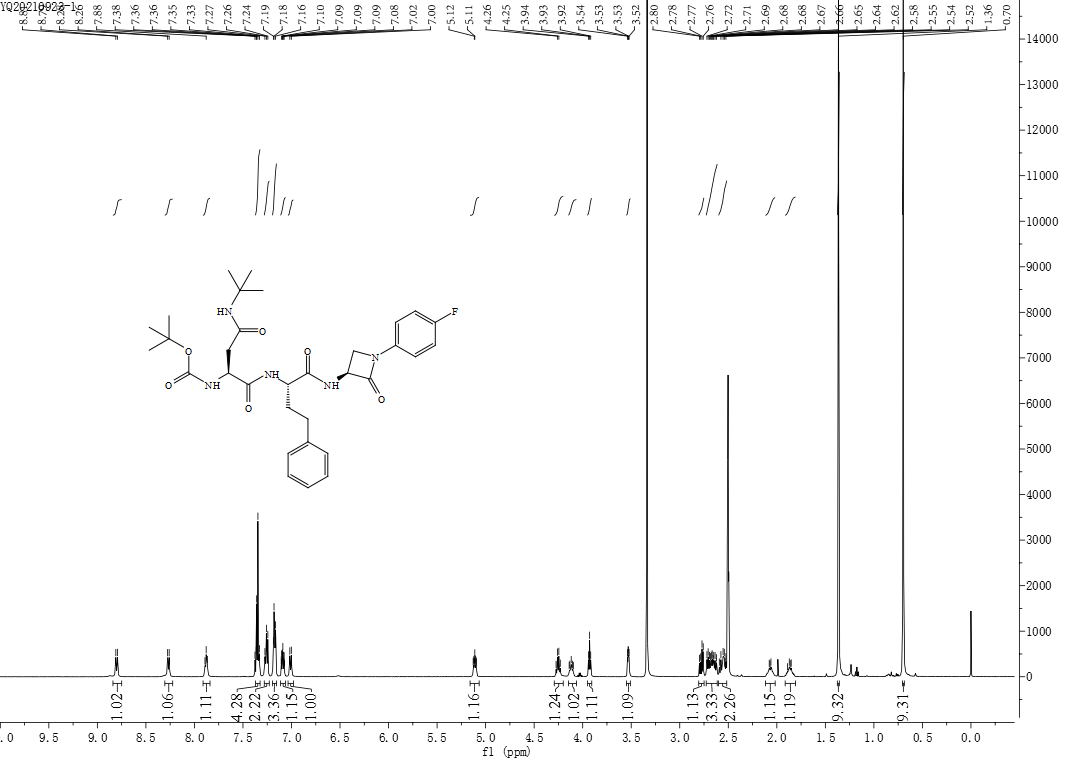


**Figure S74**. ^1^H NMR spectrum of compound **39** in DMSO-*d*_6_


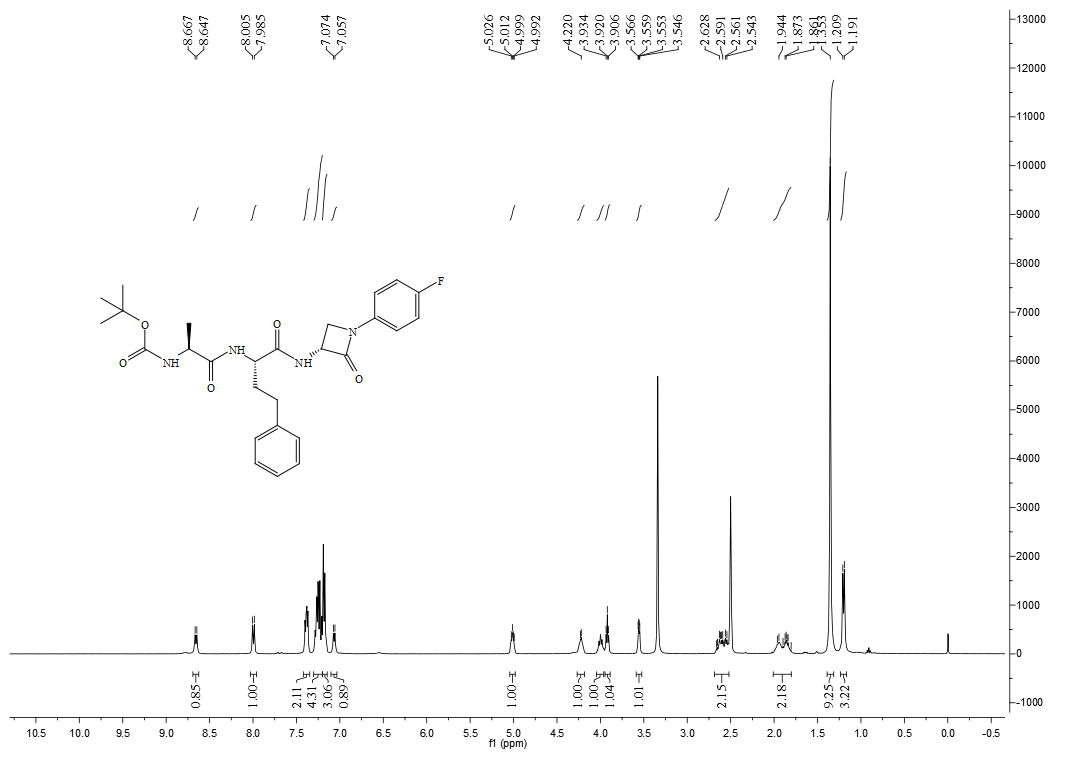


**Figure S75**. ^1^H NMR spectrum of compound **42** in DMSO-*d*_6_


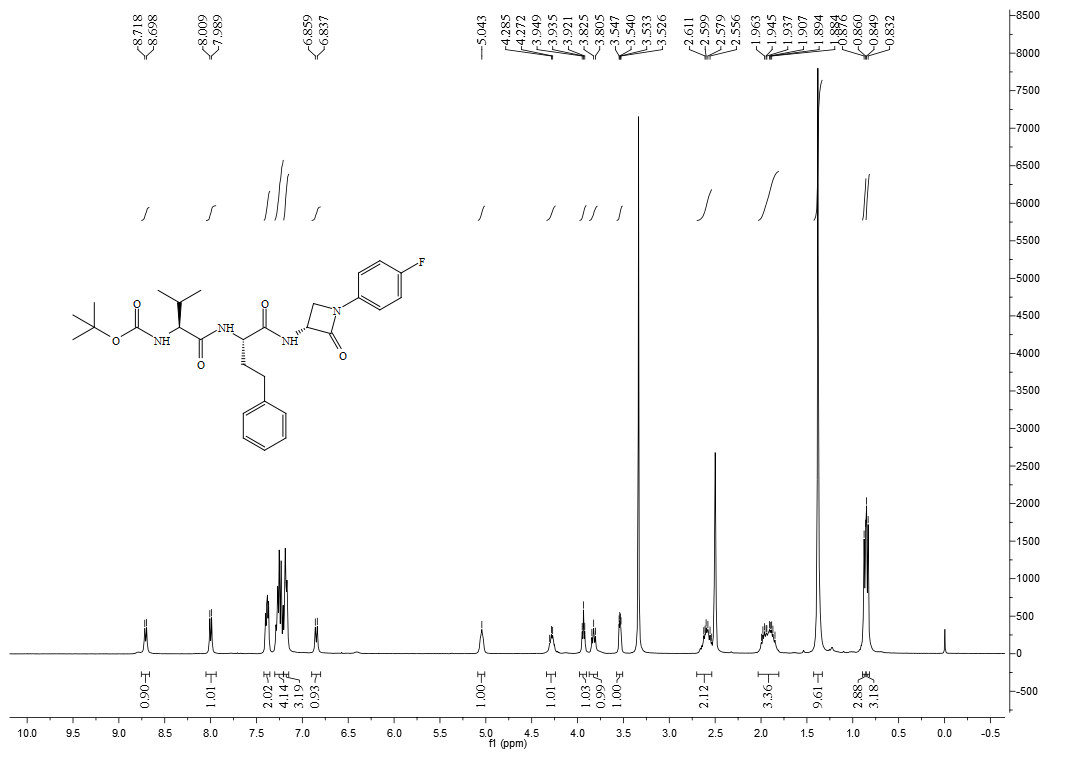


**Figure S76**. ^1^H NMR spectrum of compound **43** in DMSO-*d*_6_


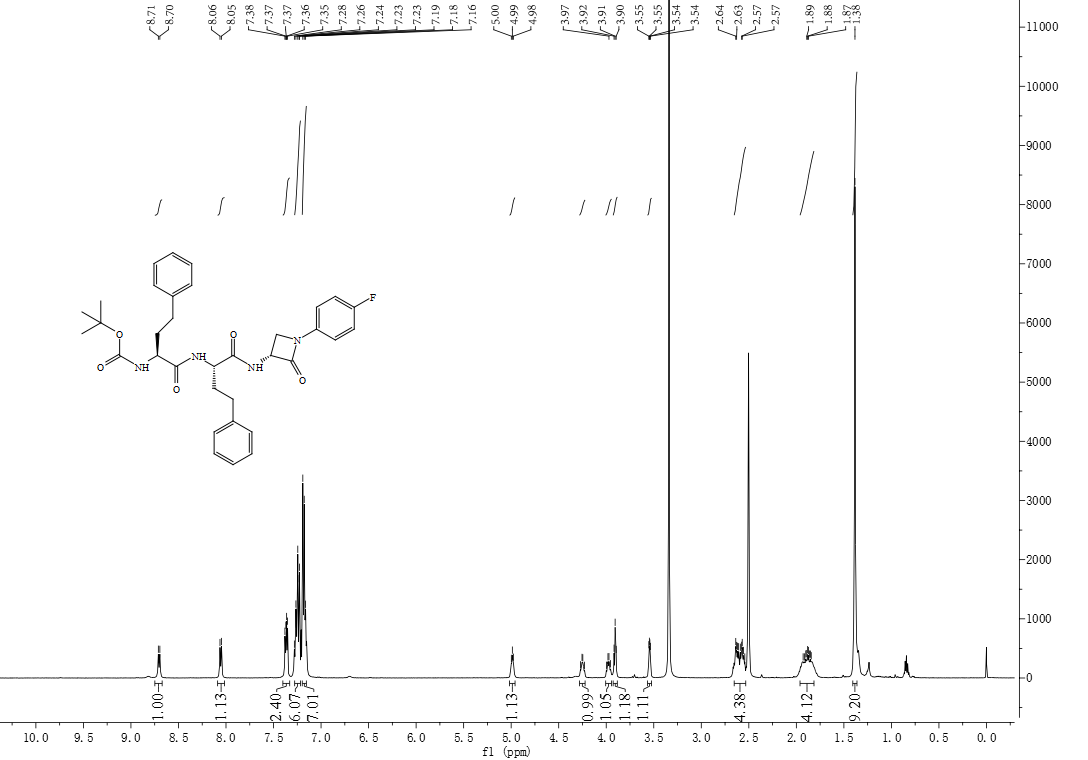


**Figure S77**. ^1^H NMR spectrum of compound **44** in DMSO-*d*_6_


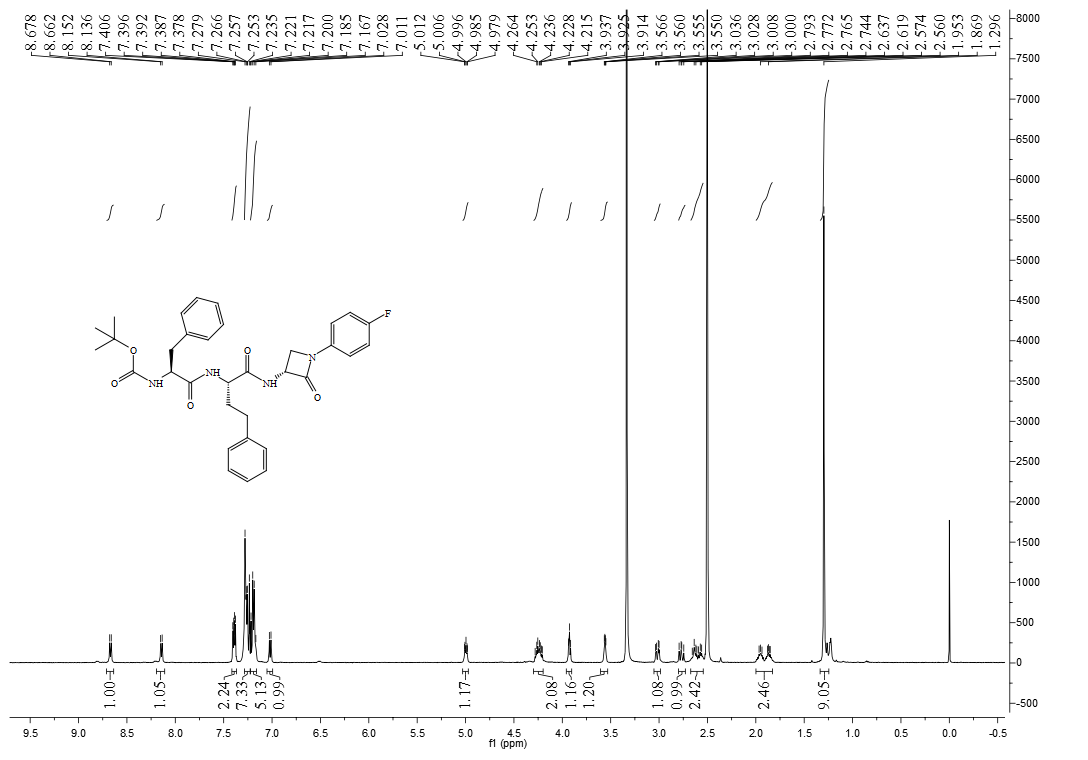


**Figure S78**. ^1^H NMR spectrum of compound **45** in DMSO-*d*_6_


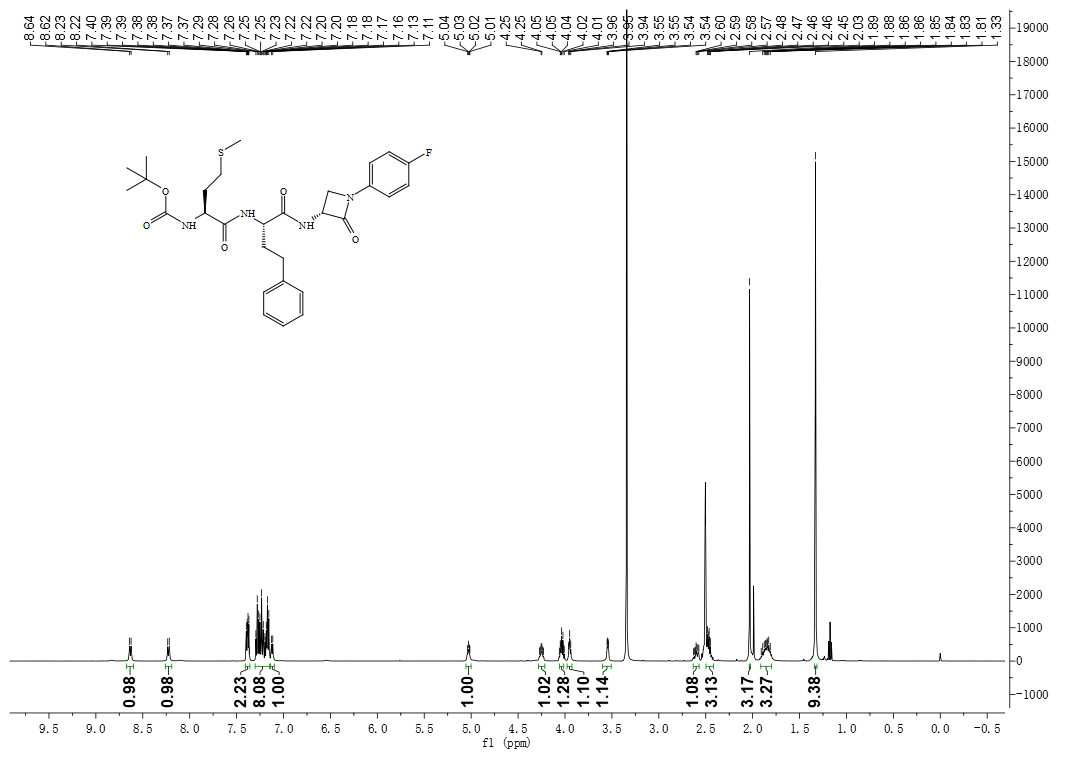


**Figure S79**. ^1^H NMR spectrum of compound **46** in DMSO-*d*_6_
